# Steric Coordination Modulated Iodine Chemistry With Four‐Electron Conversion for Zinc‐Iodine Batteries

**DOI:** 10.1002/adma.73681

**Published:** 2026-06-11

**Authors:** Shuai Wang, Haoran Wang, Yujue Yang, Yuanyuan Gao, Yaopeng Wu, Junze Zhang, Jingxin Zhao, Yuejiao Chen, Bingang Xu

**Affiliations:** ^1^ Research Institute for Intelligent Wearable Systems The Hong Kong Polytechnic University Hong Kong Hung Hom China; ^2^ Nanotechnology Center School of Fashion and Textiles The Hong Kong Polytechnic University Hong Kong Hung Hom China; ^3^ School of Ocean Information Engineering Fujian Provincial Key Laboratory of Oceanic Information Perception and Intelligent Processing Jimei University Xiamen China; ^4^ State Key Laboratory For Powder Metallurgy Central South University Changsha China

**Keywords:** enhanced performance, four‐electron conversion, halogen chemistry, hydrolysis reaction, steric‐hindrance effect

## Abstract

The advancement of high‐voltage aqueous zinc‐iodine batteries is impeded by the instability of I^+^ intermediates during the conversion process, which suffers from hydrolysis and poor reversibility in conventional electrolytes. To overcome these challenges, we propose a steric coordination strategy employing Cl^−^ and sulfonate‐rich TES^−^ ions to modulate the coordination environment of I^+^ ions. Cl^−^ ions activate I^+^ ions through halide coordination, while the steric‐hindrance effect of TES^−^ within the TES‐I‐Cl coordination structure effectively shields I^+^ ions from nucleophilic attacks by water‐derived hydroxyl groups, collectively facilitating the reversible I^−^/I^0^/I^+^ four‐electron conversion. Concurrently, adsorbed ions (TES^−^, Ch^+^, Cl^−^) establish an electrostatic shielding layer to homogenize zinc deposition and form a dehydrated electric double layer to mitigate zinc corrosion. Moreover, the disruption of H‐bond networks between the water molecules induced by the additives reduces the water activity, further suppressing I^+^ hydrolysis and water dissociation. Benefiting from these synergetic effects, the zinc‐iodine battery achieves highly reversible and stable iodine chemistry, including a high‐rate capability and long‐term cycling stability over 42 000 cycles (capacity retention: ∼70%). This work provides fundamental insights into ion coordination chemistry for designing high‐energy‐density aqueous iodine storage.

## Graphical abstract

A dual‐additive electrolyte strategy is developed to address the hydrolysis of I^+^ in the aqueous electrolytes. The steric‐hindrance effect of TES^−^ effectively shields I^+^ from nucleophilic attacks by hydroxyl groups, facilitating the reversible I^−^/I^0^/I^+^ conversion with four‐electron transfer. As a result, the assembled zinc‐iodine battery achieves highly reversible and stable iodine chemistry, particularly for operation in extreme environments.

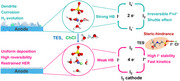

## 1 Introduction

Rechargeable aqueous zinc‐based batteries are considered as one of the most promising next‐generation energy storage systems owing to their intrinsic safety, low cost, and environmental compatibility [1, 2, 3, 4, 5, 6]. Nevertheless, their practical energy density remains constrained by the limited operating voltage and capacity of conventional cathode materials [7, 8, 9, 10, 11]. Recent research has focused on developing high‐voltage and high‐capacity cathode materials, including vanadium/manganese‐based oxides [12, 13, 14, 15, 16, 17, 18], redox‐active organic compounds [19, 20, 21, 22, 23], and conversion‐type halogen‐based materials [24, 25, 26, 27, 28, 29, 30, 31, 32, 33]. Among these, iodine‐based cathodes have garnered considerable interest because of the tunable valence states of iodine [34]. Traditional zinc‐iodine batteries primarily rely on the reversible I^−^/I° conversion with a two‐electron transfer process (I^−^/I^0^, 0.54 V *vs*. SHE, 211 mAh g^−1^), while the successive I^−^/I^0^/I^+^ conversion with a four‐electron transfer process could offer a higher redox potential and double theoretical capacity (I^−^/I^0^/I^+^, 0.54/1.07 V *vs*. SHE, 422 mAh g^−1^) [35, 36, 37]. However, unlike I_2_ or I^−^ ions, positive I^+^ ions face thermodynamic instability and high susceptibility to hydrolysis in conventional aqueous electrolytes, posing a significant challenge in activating the I^0^/I^+^ conversion, which leads to poor stability and reversibility of I^0^/I^+^ redox chemistry [38, 39, 40].

The electro‐oxidation of iodine generates thermodynamically favorable I^+^ intermediates through nucleophilic interactions with species such as halides, cyanide, and amines [41, 42, 43, 44]. However, these intermediates remain vulnerable to hydrolysis via nucleophilic attacks from the hydroxyl groups in water molecules [45]. This instability results in challenges for practical applications, including low Coulombic efficiency and compromised cycling life, necessitating advanced strategies to suppress hydrolysis reactions in zinc‐iodine batteries. Simultaneously, zinc anodes often face challenges such as water‐induced side reactions and dendrite formation [46, 47, 48]. These critical issues collectively result in the poor lifespan of four‐electron zinc‐iodine batteries [49]. To address the above issues, it is necessary to promote the reversible I^0^/I^+^ conversion by regulating the electrolyte composition or solvation structure. Approaches such as concentrated electrolytes [38, 50], organic‐aqueous hybrid electrolytes [51], and eutectic electrolytes [43], have been developed to suppress the hydrolysis of I^+^ by decreasing the activity of water molecules in the electrolytes. In addition, the introduction of halide anions, such as Cl^−^ and Br^−^ ions, into conventional electrolytes can promote the formation and stabilization of the I^+^ intermediates through halide coordination, and has been demonstrated as an effective route to activate high‐valence iodine chemistry for four‐electron zinc‐iodine batteries [42, 44, 52, 53]. Nevertheless, the stability of I^+^ in these systems is predominantly governed by the availability of free halide ions in the electrolytes, while halide coordination alone remains insufficient to fully suppress I^+^ hydrolysis in aqueous electrolytes. Concurrently, cation coordination/complexation effects or adsorption‐catalysis processes [36, 54, 55, 56], which can modulate the solvation or interfacial environment of I^+^, have also been demonstrated to effectively facilitate reversible I^0^/I^+^ conversion. Most of these strategies mainly focus on cathodic iodine regulation and are limited to addressing a single issue in four‐electron zinc‐iodine batteries, making it highly challenging to develop approaches that can simultaneously optimize I^+^ stability, zinc anode reversibility, and rate performance at low temperatures.

**FIGURE 1:**
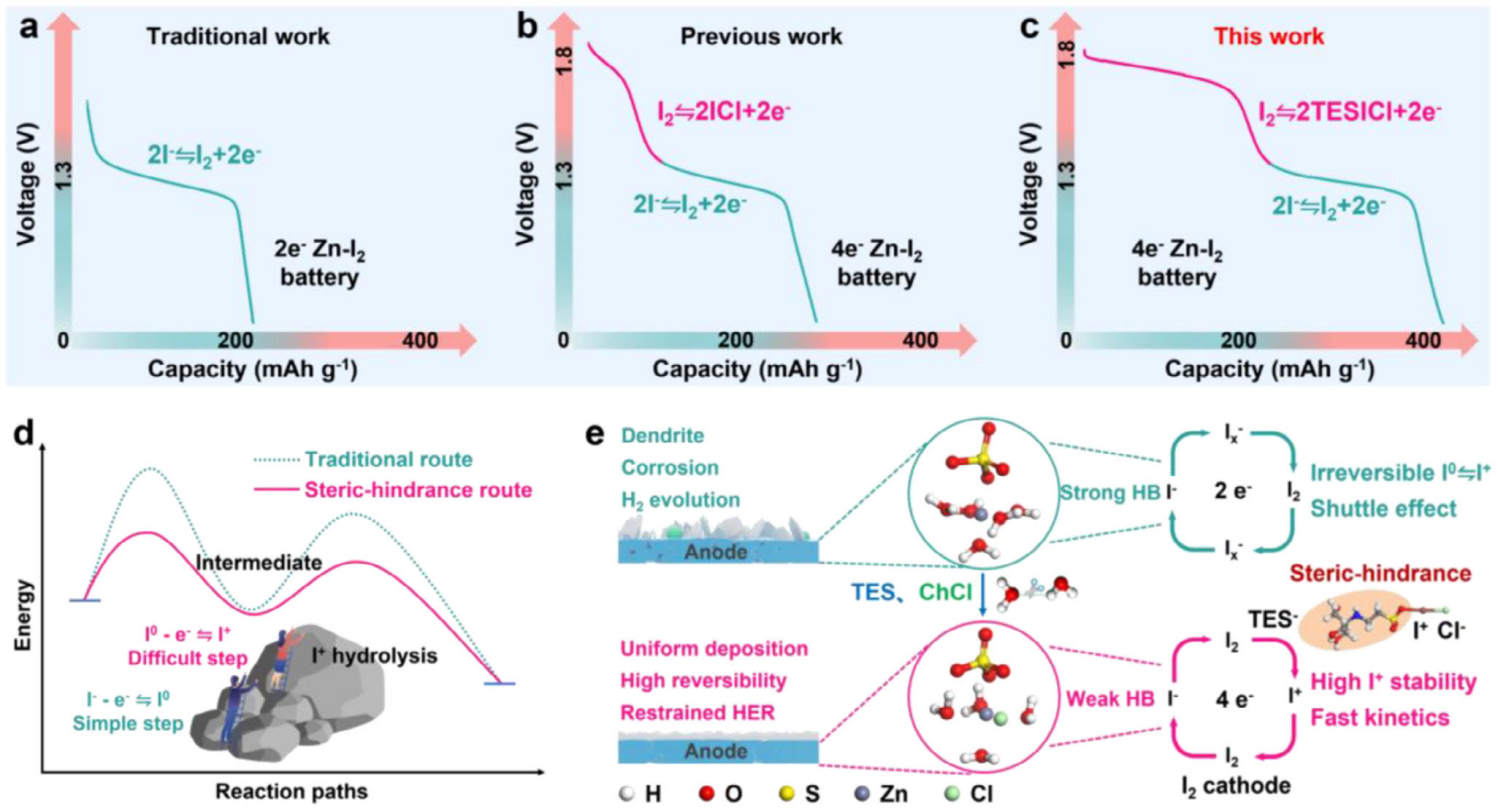
Schematic illustration of different zinc‐iodine batteries and their influences on iodine redox. (a) Traditional two‐electron‐transfer zinc‐iodine battery. (b) Halogen coordination‐based four‐electron transfer zinc‐iodine battery. (c) Steric‐hindrance effect‐based four‐electron transfer zinc‐iodine battery. (d) Schematic illustration of the functions and properties with traditional and steric‐hindrance routes; Inset shows the illustration of the reactions of iodine batteries, reversible I^−^/I° conversion at low potential can be easily achieved, but the desired reversible I^0^/I^+^ conversion at high potential is difficult due to the unstable I^+^ in the traditional route. (e) Schematic illustrations of traditional zinc‐iodine batteries with critical problems and the mechanism of electrolyte additives in regularizing the anode and cathode interface simultaneously.

Herein, the steric‐hindrance effect on the coordination structure of I^+^ was investigated using dual‐electrolyte additives, choline chloride and 2‐[2‐Hydroxy‐1,1‐bis (hydroxymethyl) ethylamino] ethanesulfonic acid sodium salt (TES‐Na), which contain Cl^−^ and sulfonate groups (R‐SO_3_^−^) ions, respectively, to achieve advanced zinc‐iodine batteries with four‐electron conversion (Figure 1a–c). In such an optimized electrolyte, the presence of Cl^−^ ions can activate the I^0^/I^+^ redox chemistry and strengthen the coordination structure. Meanwhile, the steric‐hindrance effect of sulfonate‐containing TES^−^ groups within the TES‐I‐Cl coordination structure effectively protects the Cl^−^‐activated I^+^ species from nucleophilic attack by water‐derived hydroxyl groups and significantly increases the hydrolysis energy barrier, thereby boosting the successive I^−^/I^0^/I^+^ conversion and reducing the reaction barrier with four‐electron transfer in the aqueous zinc‐iodine batteries (Figure 1d). Furthermore, the adsorption of bulky ions (TES^−^, Ch^+^, and Cl^−^) not only creates an electrostatic shielding layer to homogenize zinc deposition but also provides a water‐poor electric double layer to suppress water‐induced side reactions on the zinc anode. In addition, the introduction of additives can disrupt the H‐bond networks between the water molecules, which weakens the water activity, further preventing the I^+^ hydrolysis and suppressing the water ionization (Figure 1e). As a result, the zinc‐iodine battery in such an aqueous electrolyte exhibits enhanced I^−^/I^0^/I^+^ conversion and prolonged cycling stability.

## 2 Results and Discussion

### 2.1 Electrolyte Structures and H‐Bond Characterizations

In aqueous batteries, the H‐bond networks between water molecules facilitate the freezing of water at sub‐zero temperatures and promote the ionization of water at high voltages. Therefore, reconstructing these H‐bond networks through electrolyte engineering is an effective strategy to overcome these problems under extreme conditions. In this work, various electrolytes were fabricated by introducing dual additives with different molar concentrations (choline chloride and TES‐Na) into the 2 M ZnSO_4_ solution, labeled as 2 M ZS, ZSCC, and ZSCCT‐x, respectively (see Experimental section for details). The ionic conductivities of the various electrolytes were analyzed by electrochemical impedance spectroscopy (EIS). The results show that the ionic conductivity gradually increases with the addition of ChCl and TES‐Na (Figure S1), owing to both the increased ionic concentration from additive dissociation and the bridging effect of the additive ions [57, 58], which restrains the polarization of water molecules and enhances the conduction of Zn^2+^ ions [59]. From the 2 M ZS to the ZSCCT‐3 electrolyte, the viscosity increases progressively from 3.63 to 6.06 mPa∙s due to the addition of ChCl and TES‐Na (Figure S2). Despite the viscosity increases, the ionic conductivity also increases continuously from 16.33 to 23.34 mS cm^−1^ (Figure S1). This suggests that within the concentration range studied, the beneficial effect of increased carrier concentration dominates over the detrimental effect of increased viscosity.

**FIGURE 2:**
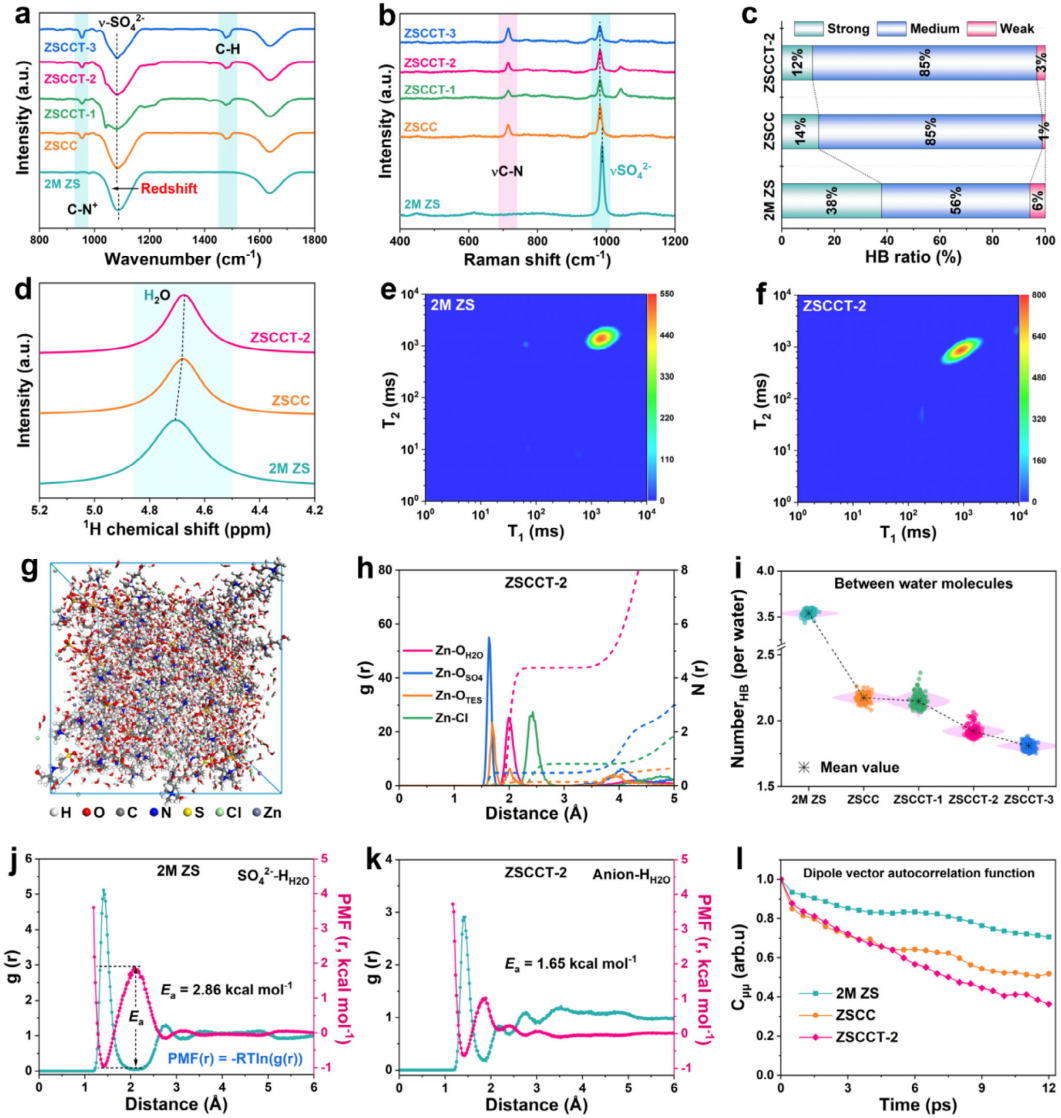
Electrolyte structures and H‐bond characterizations of the various electrolytes. (a) FTIR spectra. (b) Raman spectra. (c) Proportions of strong, medium, and weak H‐bonds. (d) ^1^H NMR spectra. 2D LF‐NMR T_1_‐T_2_ relaxation spectra of the (e) 2 M ZS and (f) ZSCCT‐2 electrolytes. (g) MD simulation snapshot and (h) RDFs and the corresponding average coordination number of the ZSCCT‐2 electrolyte. (i) Mean values of the H‐bond number between the water molecules. RDFs and PMFs of anion‐H_H2O_ atom pairs in the (j) 2 M ZS and (k) ZSCCT‐2 electrolytes. (l) Dipole vector autocorrelation function (DACF) curves of the various electrolytes.

Fourier transform infrared (FTIR) spectra were carried out to verify the influence of additives on the evolution of H‐bond networks and solvation structures in the various electrolytes (Figure 2a and Figure S3). The peaks of O─H stretching vibration (3000–3600 cm^−1^) in water exhibit a blue‐shift with the addition of ChCl and the increase of molar concentration of TES‐Na, indicating an abatement of initial H‐bond networks between the water molecules [60], while the H‐bond networks between the additives and water molecules are strengthened. Moreover, the *v*‐SO_4_2^−^ stretching located at ∼1085 cm^−1^ exhibits a red‐shift, confirming changes in solvation structure following the introduction of additives [61]. These variations are assigned to the bond interactions between the additive ions (such as Ch^+^, Cl^−^, and TES^−^) and water molecules, which are further supported by Raman and nuclear magnetic resonance (NMR) spectra. In the Raman spectra (Figure 2b and Figure S4), the *v*‐SO_4_2^−^ and O─H stretching vibrations display the same offset direction as the FTIR results, confirming the changes in solvation structures and H‐bond networks. Raman spectra reveal a noticeable blue shift in the O─H stretching peaks with the addition of ChCl and TES‐Na, consistent with the FTIR results. This shift confirms the weakening of intrinsic H‐bonds between water molecules due to the disruption of the original H‐bond networks. In addition, owing to the different H‐bond environments, the O─H stretching vibrations reflecting H‐bond environments are fitted into strong, medium, and weak H‐bonds (Figure 2c). The proportion of strong H‐bonds decreases from 38% in the 2 M ZS electrolyte to 12% in the ZSCCT‐2 electrolyte due to the reconstruction of H‐bond networks under the influence of additive ions. In the NMR spectra, the addition of ChCl and TES‐Na induces a shift in the ^1^H peak toward a lower chemical shift, demonstrating an increase in the electron density of protons in water and a weakening of H‐bonds caused by the charge transfer between the additive ions and water molecules (Figure 2d). The relaxation dynamics evolution of water molecules was elucidated by the 2D low‐field NMR spectra (Figure 2e,f, Figures S5 and S6). The time regions (T_1_) in the range of <1,000 and > 1,000 ms are attributed to the immobilized and free water molecules, respectively. Obviously, excess free water is observed in the 2 M ZS electrolyte, while the proportion of immobilized water molecules can be neglected. After adding the additives, the T_1_ peaks experience a downshift in the ZSCC and ZSCCT‐2 electrolytes, suggesting that the H‐bond number among water molecules is effectively reduced by the reconstruction of H‐bond networks, further reducing the water activity in the electrolytes. Through the contact angle tests of the various electrolytes on cathode surfaces, we found that the electrolyte wettability is improved with the addition of ChCl and TES‐Na (Figure S7). This phenomenon may be attributed to the disruption of H‐bond networks by additives, which reduces the interfacial tension (IFT) values of the electrolytes (Table S1). Simultaneously, lowered water activity suppresses the hydrogen and oxygen evolution reactions, thereby expanding the electrochemical window (Figure S8).

The evolution of solvation structures and H‐bond networks in the various electrolytes was identified by the molecular dynamics (MD) simulations (Figure 2g, Figure S9, and Table S2). Radial distribution functions (RDFs) simulated the distribution of coordination molecule/ion around Zn^2+^. The two sharp peaks around 1.6 and 2.0 Å represent Zn^2+^‐O(SO_4_2^−^) and Zn^2+^‐O(H_2_O) coordination structures in the 2 M ZS electrolyte, with coordination numbers *N*(r) of H_2_O and SO_4_2^−^ in the Zn^2+^‐solvation shell being 5.3 and 0.7, respectively (Figure S10a). After adding ChCl (ZSCC electrolyte), a new Zn‐Cl coordination structure appears at 2.4 Å due to the strong binding ability between the Zn^2+^ and Cl^−^ ions [60]. As a result, the *N*(r) of Zn^2+^‐O(H_2_O) decreases significantly from 5.3 to 4.4, while the *N*(r) of Zn^2+^‐Cl^−^ reaches 0.9, indicating that a water molecule in the Zn^2+^‐solvation shell is replaced by a Cl^−^ ion (Figure S10b). With the addition of TES‐Na, the coordination structure hardly changes in the ZSCCT‐1 and ZSCCT‐2 electrolytes (Figure 2h and Figure S10c). Notably, owing to the continuous increase in TES concentration, the *N*(r) of TES^−^ in the Zn^2+^‐solvation shell increases by 0.3 in the ZSCCT‐3 electrolyte (Figure S10d), meaning that a small number of TES^−^ ions participate in the Zn^2+^ coordination at high TES‐Na concentrations. These findings are consistent with the above‐mentioned FTIR and Raman results. Figure S11 records the most probable Zn^2+^‐solvation structures from the MD simulations. The MD simulations were also carried out to obtain the average number of H‐bonds in the various electrolytes. The results demonstrate that the additives inhibit the formation of continuous H‐bond networks between the water molecules, thereby reducing the mean values of H‐bonds in water along with the addition of additives (Figure 2i). In contrast, the mean values of H‐bonds between the additive ions and water molecules increase with the introduction of additives due to the reconstruction of H‐bond networks (Figure S12).

RDF and potential of mean force (PMF) were conducted to reveal the local structures and diffusion barriers of water molecules in these systems involving various anions (Figure 2j,k and Figure S13). In the 2 M ZS electrolyte, the RDF of SO_4_2^−^‐H(H_2_O) displays a distinct peak at 1.41 Å, indicating an ordered distribution of water molecules around SO_4_2^−^. Similar RDFs with peaks at 1.42 Å were observed in the ZSCC and ZSCCT‐2 electrolytes. However, the anion‐H(H_2_O) pair in the ZSCCT‐2 electrolyte shows a weaker peak, suggesting some disorganization within the anion solvation shell [62]. The diffusion barriers of water molecules in the first hydration shells are calculated by the PMF values. After the introduction of dual additives, the PMF values (2.20 and 1.65 kcal mol^−1^) for the ZSCC and ZSCCT‐2 electrolytes are significantly lower than that of the 2 M ZS electrolyte (2.86 kcal mol^−1^). The relatively weak interaction between the additive anions and water molecules leads to a more disordered distribution and accelerates the diffusion kinetics of water molecules. The dipole vector autocorrelation function (DACF) further quantifies the dynamic relaxation behaviors of water molecules (Figure 2l) [63]. The trends in relaxation rates indicate that the ZSCCT‐2 electrolyte exhibits a faster relaxation rate, suggesting more energetic molecule kinetics, which is associated with the disruption of H‐bond networks among water molecules. These results demonstrate that the addition of additives can facilitate the mass transfer and uniform deposition of Zn^2+^.

### 2.2 Zn Plating/Stripping Behaviors in Various Electrolytes

**FIGURE 3:**
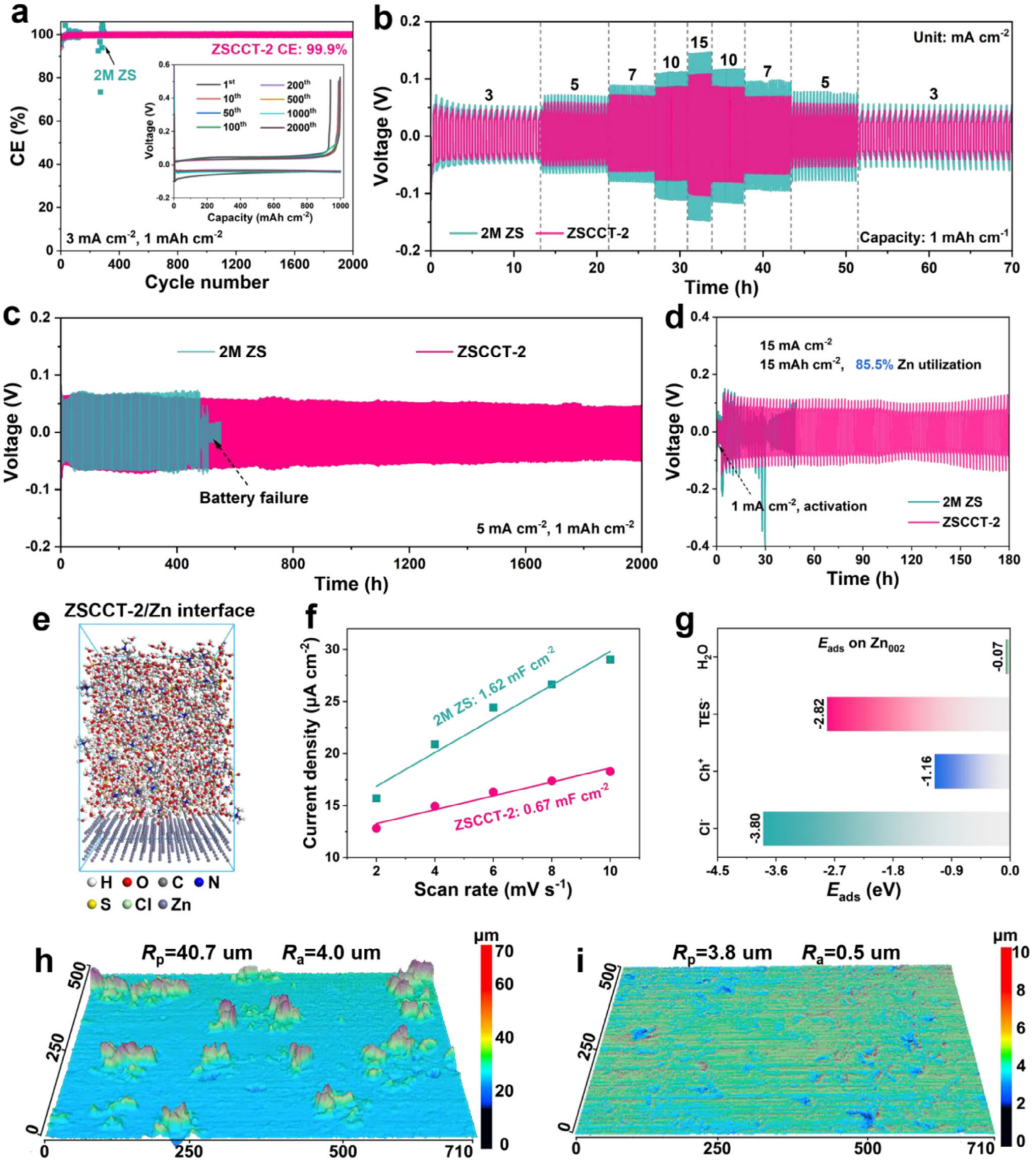
Zn plating/stripping behaviors in the various electrolytes. (a) CE measurements at 3 mA cm^−2^, 1 mAh cm^−2^. (b) Rate capability. (c) Cycling performance at 5 mA cm^−2^, 1 mAh cm^−2^. (d) Cycling performance at 15 mA cm^−2^, 15 mAh cm^−2^ with 85.5% Zn utilization. (e) Surface energy for metallic Zn when exposed in the ZSCCT‐2 electrolyte. (f) Plots of capacitive currents versus scan rates in the various electrolytes. (g) Values of surface adsorption energy of Cl^−^, Ch^+^, TES^−^, and H_2_O on Zn_[002]_ plane. 3D LSCM images of Zn electrodes after 20 cycles in the (h) 2 M ZS and (i) ZSCCT‐2 electrolytes.

The Zn||Cu and Zn||Zn cells were employed to assess the reversibility of Zn in the various electrolytes. As evidenced in Figures S14 and S15, the cell with the 2 M ZS electrolyte displays fluctuations after hundreds of cycles due to the side reactions and dendritic growth, while the cells with ZSCC, ZSCCT‐1, and ZSCCT‐2 electrolytes maintain stable Coulombic efficiencies (CEs) of 99.03%, 99.25%, and 99.36%, respectively, at 0.5 mA cm^−2^, 0.5 mAh cm^−2^ over 500 cycles. By contrast, the cell with ZSCCT‐3 electrolyte exhibits inferior reversibility of the Zn plating/stripping process. It may be ascribed to the participation of TES^−^ in the Zn^2+^‐solvation shell, where TES^−^ possesses a relatively small energy gap between the highest occupied molecular orbital (HOMO) and the lowest unoccupied molecular orbital (LUMO) (Figures S16 and S17), resulting in the high reaction activity of TES^−^. The poor reversibility of Zn in the ZSCCT‐3 electrolyte is further confirmed through the cyclic voltammetry (CV) test of a zinc‐iodine full battery and Zn||Zn cell (Figure S18). Even at a high current density of 3 mA cm^−2^, a high average CE of 99.9% is achieved with the ZSCCT‐2 electrolyte (Figure 3a and Figure S19). As discussed above, the ZSCCT‐2 electrolyte is considered the optimized electrolyte and has been employed to assess the electrochemical performance of the zinc‐iodine batteries.

The CV curves of Zn||Cu asymmetric cells are performed to evaluate the Zn^2+^ deposition/dissolution behavior in the various electrolytes. As illustrated in Figure S20a, the CV curves clearly display the characteristic Zn^2+^ deposition and dissolution peaks for both electrolytes. Notably, the ZSCCT‐2 electrolyte exhibits higher peak current densities compared to 2 M ZS, indicating enhanced electrochemical activity and improved Zn^2+^ kinetics. Furthermore, the voltage gap (ΔV) between the deposition and dissolution peaks is reduced in the ZSCCT‐2 electrolyte (ΔV_2_ = 0.449 V) compared to 2 M ZS (ΔV_1_ = 0.472 V), as shown in Figure S20b. This smaller voltage gap suggests lower polarization and improved reversibility of the Zn plating/stripping process in the ZSCCT‐2 electrolyte. The fast ion and charge transfer kinetics endow the Zn||Zn cell with enhanced rate capability in the ZSCCT‐2 electrolyte (Figure S21). The battery with the ZSCCT‐2 electrolyte exhibits smaller voltage hysteresis compared to the cell with the 2 M ZS electrolyte as the current density increases from 3 to 15 mA cm^−2^ (Figure 3b), which is due to the increased Zn^2+^ transference number in the ZSCCT‐2 electrolyte (Figures S22 and S23). Furthermore, the Zn||Zn cell with the ZSCCT‐2 electrolyte exhibits a stable cycling life over 2,000 h at 5 mA cm^−2^, 1 mAh cm^−2^ (Figure 3c and Figure S24). More importantly, the cell with the ZSCCT‐2 electrolyte can still demonstrate good cycling stability over 180 h at a high zinc utilization rate of ∼85.5% (Current density: 15 mA cm^−2^), while its counterpart in the 2 M ZS electrolyte can barely work (Figure 3d). The electrochemical behaviors of Zn during the continuous plating/stripping process are predominantly determined by the atomic configuration of the reaction surface, as evidenced by the corresponding surface energy characteristics [64]. The ZSCCT‐2/Zn interface shows a higher surface energy of 2.26 kcal mol^−1^ Å^−2^ than the case (1.84 kcal mol^−1^ Å^−2^) of the 2 M ZS/Zn interface (Figure 3e, Figures S25 and S26), explicating that the addition of additives guides the zinc growth and inhibits zinc dendrite formation [61]. To evaluate the adsorption phenomenon of multiple additive ions on the zinc surface, the electric double layer (EDL) capacitance was investigated by the CV curves of the Zn||Zn cells (Figure S27). The Zn||Zn cell exhibits a lower EDL capacitance of 0.67 mF cm^−2^ in the ZSCCT‐2 electrolyte than the case (1.62 mF cm^−2^) of the 2 M ZS electrolyte (Figure 3f). The decrease of EDL capacitance is attributed to the formation of an ion adsorption layer near zinc due to the strong adsorption ability between the additive ions and zinc plane (Figure 3g and Figure S28), thus establishing a water‐poor EDL, which is beneficial to inhibit side reactions on the zinc surface [65]. Scanning electron microscopy (SEM) and laser scanning confocal microscopy (LSCM) were conducted to elucidate the morphology of zinc deposition. The inhomogeneous distribution of petal‐like zinc dendrites is formed on the zinc anode in the 2 M ZS electrolyte, but no discernible zinc dendrites are observed on the zinc anode in the ZSCCT‐2 electrolyte (Figure S29). In addition, the roughness values (R_p_ = 3.8 µm, R_a_ = 0.5 µm) of deposited zinc in the ZSCCT‐2 electrolyte are smaller than those (R_p_ = 40.7 µm, R_a_ = 4.0 µm) in the 2 M ZS electrolyte (Figure 3h,i and Figure S30). All these results visually show the promotion of uniform growth of zinc in the optimized electrolyte, resulting in excellent cycling stability.

### 2.3 Hydrolysis Inhibition and Conversion Mechanism

**FIGURE 4:**
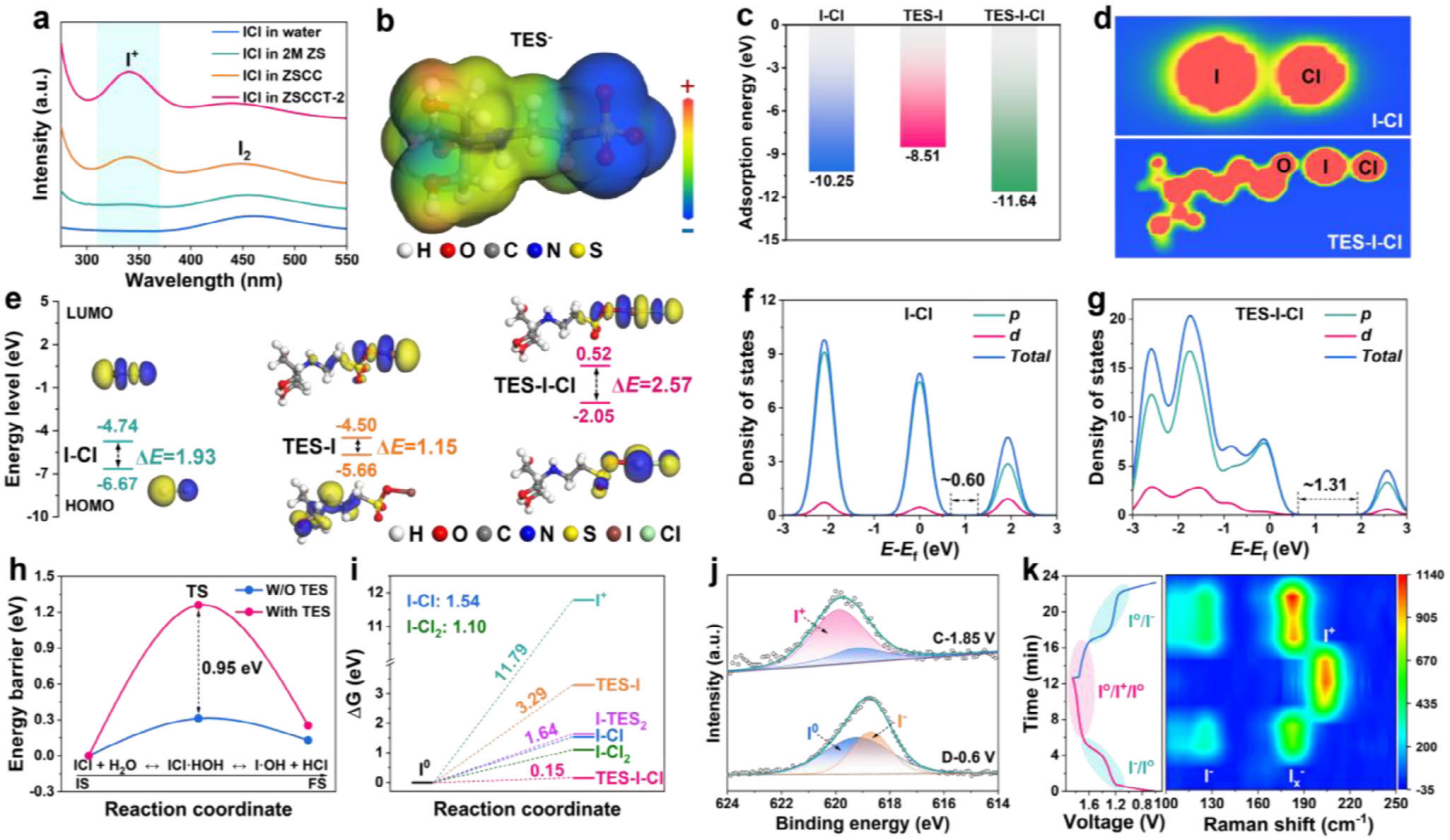
Hydrolysis inhibition and conversion mechanism of high‐voltage zinc‐iodine batteries. (a) UV‐vis spectra of ICl in the different solutions. (b) MESP mapping of TES^−^. (c) Adsorption energies, (d) ELF patterns, (e) frontier molecular orbital energy levels, and (f,g) density of states of I^+^ with different species. (h) The energy barriers of ICl hydrolysis reaction W/ or W/O TES^−^. (i) Calculated Gibbs free energies of I^0^, I^+^, I‐Cl, I‐Cl_2_, TES‐I, I‐TES_2_, and TES‐I‐Cl coordination complexes. (j) I 3d XPS spectra of the fully discharged and fully charged cathodes. (k) Typical galvanostatic voltage profiles and Raman spectra of zinc‐iodine battery with the ZSCCT‐2 electrolyte during the charge/discharge process.

The effect of the additives in inhibiting ICl hydrolysis was investigated through ultraviolet‐visible (UV‐vis) spectroscopy. Figure 4a compares the UV‐vis spectra of ICl as an I^+^ source in water, 2 M ZS, ZSCC, and ZSCCT‐2 solutions. The spectra show the band at ∼465 nm corresponding to I_2_ in water and 2 M ZS solutions, resulting from the hydrolysis of I^+^. This band persists in the ZSCC solution, along with a small band of I^+^ at ∼345 nm due to the coordination chelation of I^+^‐Cl^−^(ChCl). Upon the addition of sulfonate‐containing TES^−^ (ZSCCT‐2 solution), the large ionic radius of TES^−^ enables the formation of I^+^ coordination structures containing TES^−^ ions, which creates a sterically crowded environment around the I^+^ center [66, 67, 68, 69]. This physical barrier effectively limits the access of nucleophilic groups in water molecules to I^+^. As a result, a distinct characteristic adsorption band of I^+^ is observed in the ZSCCT‐2 solution, indicating that the hydrolysis of I^+^ is effectively inhibited in the ZSCCT‐2 solution. The interactions between I^+^ and Cl^−^/TES^−^ ions were further explored by the density functional theory (DFT) calculations. According to the molecular electrostatic potential (MESP) and Fukui function (f^−^) of TES^−^ (Figure 4b and Figure S31), it displays more negative charges and a greater ability to donate electrons around the R‐SO_3_^−^ group than the other sites in TES^−^, meaning that TES^−^ can participate in the coordination of I^+^. To further reveal the coordination structures of I^+^ with different species, the adsorption energies of various I^+^ coordination structures with Cl^−^ and TES^−^ ions were calculated (Figure 4c, Figures S32 and S33). The results indicate that both Cl^−^ and TES^−^ ions preferentially coordinate with I^+^ to form a nearly linear structure. When attempting to construct a tridentate (three‐coordinate) complex, we observed that the third ligand is consistently repelled to a much greater distance from the iodine center (Figure S34), resulting in a loss of effective coordination. This suggests that, from a theoretical perspective, stable three‐coordinate structures of I^+^ with Cl^−^ and TES^−^ are not favored. Therefore, the formation of I‐Cl, I‐Cl_2_, I‐TES, I‐TES_2_, and TES‐I‐Cl coordination structures is both energetically and geometrically preferred, and higher coordination numbers (three‐coordinate) are unlikely under the studied conditions. Besides, these coordination structures possess uniform electron cloud distributions (Figures S35 and S36), suggesting their high stability and would help stabilize the I^+^ ion [70]. Furthermore, the analysis of atomic charge and electron localization function (ELF) results suggests that these coordination structures provide obvious electron transfer between I^+^ and Cl^−^ or TES^−^ (Figure 4d and Figures S37–S40), thereby leading to strong bonds among them. These results indicate that Cl^−^ and TES^−^ can simultaneously anchor I^+^ and form highly stable coordination structures, which helps stabilize I^+^ and further suppress its hydrolysis.

The frontier molecular orbital energy levels and density of states of coordination structures of I^+^ were also calculated by DFT calculations. Notably, among all the I^+^ coordination structures, those involving TES^−^ ion, such as I‐TES_2_ and TES‐I‐Cl, exhibit a wider energy band gap compared to other configurations (Figure 4e and Figure S41), suggesting that TES^−^‐containing complexes possess higher resistivity against hydrolysis in the aqueous solutions [71]. The result is aligned with the density of states calculations (Figure 4f,g, and Figures S42 and S43). Furthermore, we calculated the sizes of the I‐Cl, I‐Cl_2_, TES‐I, I‐TES_2_, and TES‐I‐Cl coordination structures, as shown in Figure S32. The sizes of I‐Cl and I‐Cl_2_ are only 2.387 and 5.261 Å, respectively. In comparison, the coordination structure formed by TES^−^ and I^+^ grows dramatically to 10.309 Å. Notably, TES^−^ occupies a significantly larger spatial region within the I‐TES_2_ and TES‐I‐Cl coordination structures, expanding the coordination structures to 21.139 and 13.274 Å, respectively. The introduction of TES^−^ amplifies the steric‐hindrance effect of the TES‐I‐Cl coordination structure, effectively impeding nucleophilic attack by water molecules on the I^+^ ion [69]. We also calculated the energy barriers of the ICl hydrolysis reaction with/without TES^−^. The hydrolysis process of ICl proceeds through an initial step characterized by the formation of HIO as an intermediate [45]. In this step, the dissociation of a proton from the ICl hydrate is considered the rate‐determining step, governing the overall reaction kinetics. Thus, the reaction kinetics can be evaluated through analysis of the thermodynamic free energy changes associated with the process (Figures S44 and S45). Remarkably, the energy barrier for the hydrolysis reaction with TES^−^ is calculated to be 1.26 eV, significantly higher than the 0.31 eV observed in the model without TES^−^ (Figure 4h). This can be attributed to the strong anchoring effects of Cl^−^ and TES^−^ on I^+^, along with the pronounced steric‐hindrance effect exerted by TES^−^ due to its large ionic radius [72]. To directly exhibit the steric‐hindrance effect of TES^−^, we selected methanesulfonate (Ms^−^, CH_3_SO_3_^−^) as a control anion. The rationale for this choice is that Ms^−^ possesses the same functional group (R‐SO_3_^−^) as TES^−^, and therefore exhibits a similar electronic coordination ability with I^+^ via the oxygen atoms of the sulfonate group. However, Ms^−^ anion lacks the bulky bis(hydroxymethyl)ethylamino substituent that endows TES^−^ with its large ionic radius and steric bulk. This allows us to isolate the steric contribution while keeping the electronic coordination chemistry with I^+^ nearly identical. The additional DFT calculations and electrochemical experiments were performed to compare the behavior of I^+^ in the presence of TES^−^ versus Ms^−^ (Figures S46–S54). The combined data unequivocally demonstrate that TES^−^ is far more effective than Ms^−^ in suppressing I^+^ hydrolysis and enabling stable four‐electron conversion in aqueous batteries. While both anions contain the same R‐SO_3_^−^ functional group and can coordinate with I^+^, the bulky molecular size of TES^−^ provides a critical steric‐hindrance effect that physically shields I^+^ from approaching water molecules. In contrast, the small Ms^−^ anion offers limited steric‐hindrance effect, leading to partial I^+^ hydrolysis.

More importantly, we substantiated the existence of the proposed coordination structures through Raman and FTIR analysis. The ICl solution exhibits a distinct I‐Cl stretching vibration peak at ∼268.6 cm^−1^ in the Raman spectra (Figure S55a) [73, 74, 75]. Upon the addition of ChCl (ICl+ChCl solution), the intensity of this I‐Cl peak increases significantly, indicating enhanced coordination between the Cl^−^ and I^+^ ions. When TES^−^ ions are further introduced (ICl+ChCl+TES solution), the I─Cl stretching vibration peak becomes even more pronounced, and a new characteristic peak emerges at ∼197.7 cm^−1^, which can be attributed to the I─O stretching vibration peak [76, 77]. The appearance of this I─O peak suggests that TES^−^ (containing sulfonate groups) is also participating in the coordination with I^+^, forming a TES‐I‐Cl structure. The progressive increase in the I‐Cl peak intensity with the sequential addition of ChCl and TES can be rationalized by the formation of more stable and abundant I^+^ coordination structures. The presence of both Cl^−^ and TES^−^ ions facilitates the formation of the TES‐I‐Cl structure, which is thermodynamically favored according to DFT calculations. Similarly, in the FTIR spectra (Figure S55b), the I─O stretching vibration peak is observed in the ICl+ChCl+TES solution [78], further supporting the involvement of TES^−^ in the coordination environment of I^+^. These results indicate that the I^+^ complexes involving TES^−^ possess markedly higher resistance to hydrolysis. Therefore, although various I^+^ coordination structures may coexist under dynamic equilibrium in the aqueous electrolyte, the TES‐I‐Cl coordination structure is expected to be the predominant I^+^ coordination configuration. The activation and stabilization of I^+^ by Cl^−^ synergize with the steric protection provided by TES^−^, substantially increasing the energy required for water molecules to approach the I^+^ center and form the hydrolysis transition state, thereby significantly elevating the reaction energy barrier and effectively retarding the kinetics of the hydrolysis process.

As discussed above, Cl^−^ and TES^−^ can simultaneously anchor I^+^ and inhibit its hydrolysis reaction by the steric‐hindrance effect, promoting the reversibility of I^−^/I^0^/I^+^ conversion in aqueous electrolytes. Figure 4i shows the Gibbs free energies of I_2_, I^+^, I‐Cl, I‐Cl_2_, TES‐I, I‐TES_2_, and TES‐I‐Cl complexes, respectively. The energy change (ΔG) from I^0^ to I^+^ is calculated to be 11.79 eV, which is much higher than the others for I^0^/I^+^ complexes, indicating that the process is thermodynamically unfavorable. Upon the introduction of TES^−^ and Cl^−^, some stable coordination structures are formed, benefiting from the steric hindrance effect and synergistic anchoring interactions, which significantly lowers the energy barrier for the formation of the high‐valent iodine intermediate (I^+^), thereby markedly enhancing the thermodynamic feasibility of the I^0^/I^+^ conversion [79]. The thermodynamic stability follows the order I^+^ < TES‐I < I‐TES_2_< I‐Cl < I‐Cl_2_< TES‐I‐Cl, highlighting the superior reversibility of the TES‐I‐Cl complex for I^0^/I^+^ conversion. To gain a deeper understanding of the reversible I^−^/I^0^/I^+^ redox in the optimized electrolyte, the redox transformations of iodine were further explored by the different ex situ spectroscopy technologies, including X‐ray photoelectron spectroscopy (XPS), UV‐vis, and Raman. In the XPS I 3d fitting spectra (Figure 4j), two prominent peaks are identified at around 619.2 and 618.7 eV at fully discharged to 0.6 V, corresponding to I^0^ and I^−^ species, respectively. Upon charging to 1.85 V, a new fitting peak is observed located at ∼619.9 eV, indicating the oxidation to a higher valence state of I^+^ ion [80]. The presence of I^+^ at fully charged to 1.85 V in the optimized electrolyte can also be confirmed through the Raman and UV‐vis spectra (Figure 4k and Figure S56). In the Raman spectra, the peaks of I^−^ and I_x_^−^ (125 and 182 cm^−1^) are obviously detected in the low voltage range, indicating the conversion process of I^0^ to I^−^ during the discharging process. Moreover, the peak of I^+^ at ∼205 cm^−1^ gradually appears during the charging process, while I^−^ and I_x_^−^ were barely detected, implying the complete conversion from low valence iodine to high valence iodine. The above results provide solid evidence that the proposed coordination structures can activate the reversible redox of I^0^/I^+^ and finally realize four‐electron transfer (I^−^/I^0^/I^+^) in the aqueous zinc‐iodine batteries.

### 2.4 Analysis of Interfacial Electron/Charge Transfer

**FIGURE 5:**
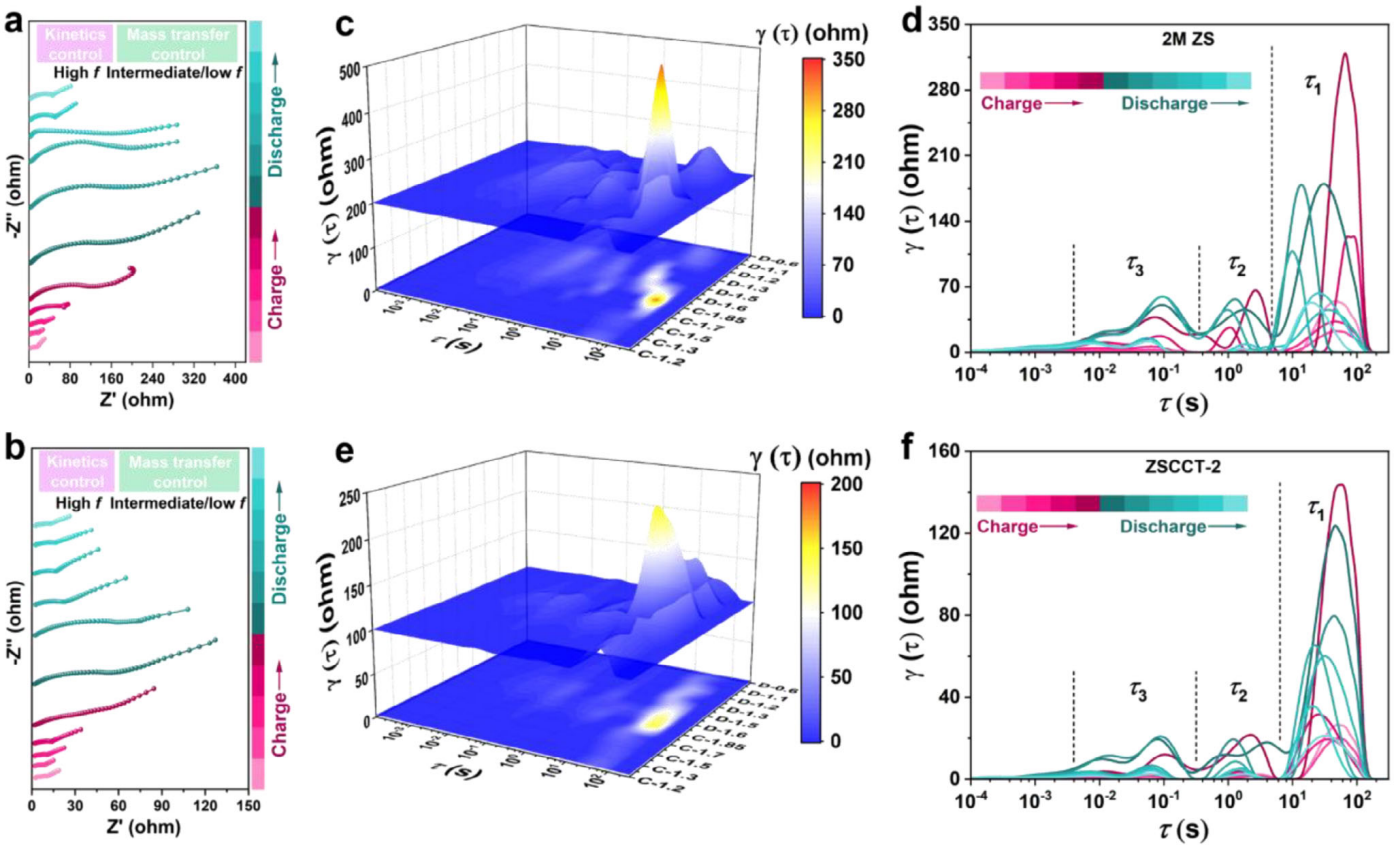
In situ EIS spectra and the corresponding DRT analysis of zinc‐iodine batteries during the charge/discharge process in the various electrolytes. EIS spectra of zinc‐iodine batteries in the (a) 2 M ZS and (b) ZSCCT‐2 electrolytes. 3D DRT patterns and 2D DRT spectra of zinc‐iodine batteries in the (c, d) 2 M ZS and (e, f) ZSCCT‐2 electrolytes.

To elucidate the effect of the proposed strategy on interfacial electron/charge transfer during the charge/discharge process, In situ EIS was conducted (Figure 5a,b). For both 2 M ZS and ZSCCT‐2 electrolytes, the overall impedance demonstrates a cyclic pattern characterized by a slow increase during the charging process, followed by a corresponding decrease during the discharge process. The distribution relaxation time (DRT) was used to probe the evolution of electron/charge transfer occurring at the interface (Figure 5c–f). In the DRT spectra, the *x*‐axis represents the relaxation time constant (*τ*), equaling resistance (*R*) times capacitance (*C*) (*τ* = *RC*), which is independent of the surface area and reflects the intrinsic property of each kinetic process [81]. Moreover, the magnitude of each peak in the DRT spectra quantitatively corresponds to the polarization resistance associated with a specific relaxation process. Consequently, the electrochemical processes characterized by distinct relaxation time constants (*τ*) manifest as distinguishable peak features in the DRT spectra, enabling their temporal separation and mechanistic identification. Typically, the DRT spectrum can be deconvoluted into three regions, denoted as *τ*_1_ (>5 s), *τ*_2_ (0.5 s < *τ* <5 s), and *τ*_3_ (<0.5 s), respectively, where *τ*_1_ is assigned to the mass transport in the bulk electrolyte, *τ*_2_ corresponds to the charge transfer process, and *τ*_3_ is ascribed to the electron transfer of the redox species. It was found that all time‐domain values (γ(*τ*)) of *τ*_1_, *τ*_2_, and *τ*_3_ of the cell with ZSCCT‐2 electrolyte are smaller than those in the 2 M ZS electrolyte, confirming that the steric‐hindrance‐optimized ZSCCT‐2 promotes faster interfacial reaction kinetics and more efficient iodine redox processes. The enhanced interfacial charge transfer and enhanced ion diffusion kinetics were also confirmed by the detailed equivalent circuit modeling, where the charge transfer resistance and diffusion impedance of the zinc‐iodine battery with the 2 M ZS electrolyte are larger than those of the ZSCCT‐2 electrolyte (Figure S57 and Table S3).

### 2.5 Electrochemical Performance of High‐Voltage Zinc‐Iodine Batteries

**FIGURE 6:**
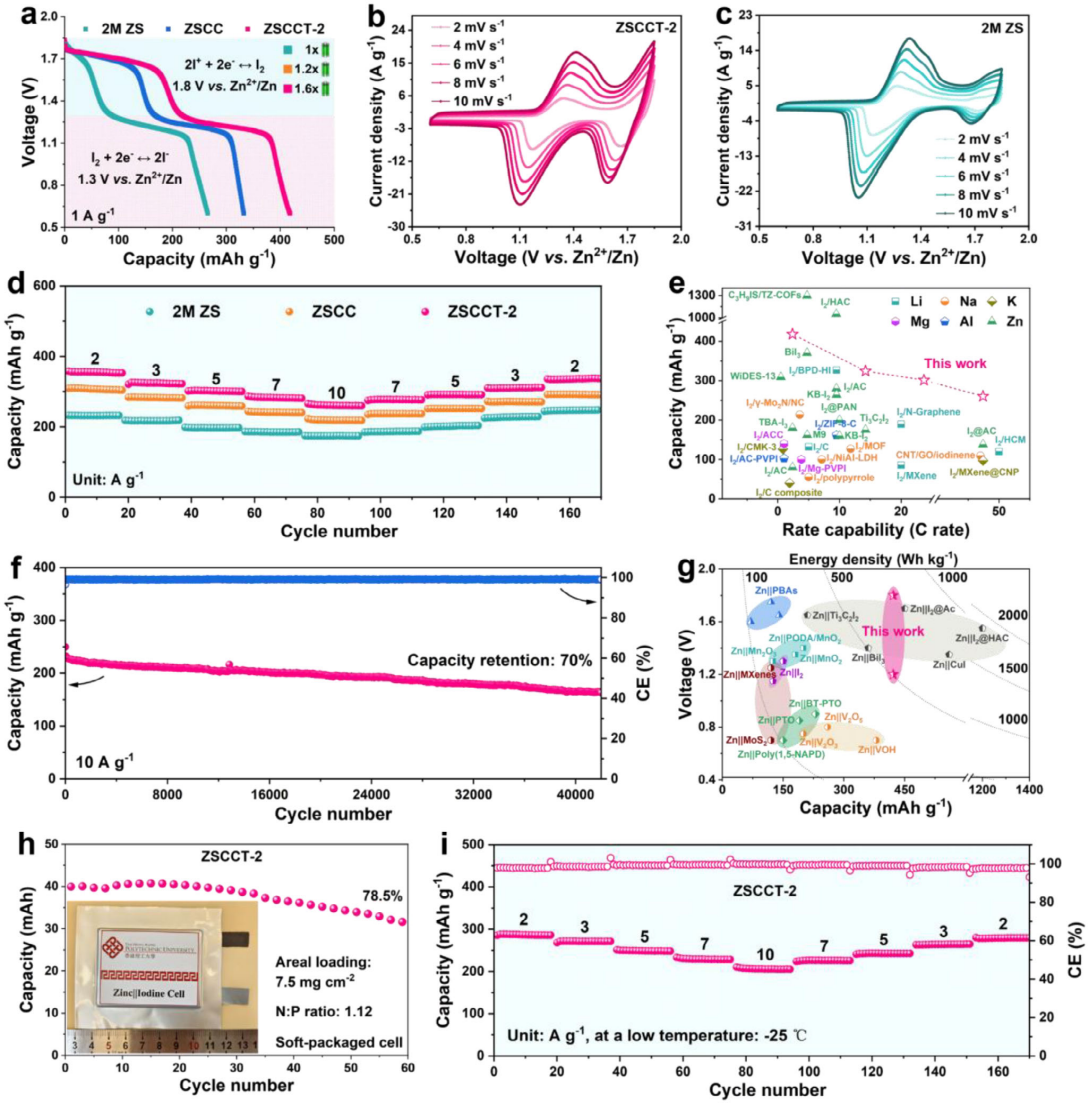
Electrochemical performance of high‐voltage zinc‐iodine batteries. (a) The discharge curves of I^0^/I^−^ reaction and I^+^/I^0^/I^−^ reaction in the various electrolytes. CV curves of zinc‐iodine batteries with the (b) ZSCCT‐2 and (c) 2 M ZS electrolytes. (d) Rate capability. (e) Comparison of rate capability of reported rechargeable metal‐iodine cells (1 C = 211 mA g^−1^ as the rate unit). The colors of dark cyan, orange, dark yellow, purple, blue, and green represent Li‐I_2_, Na‐I_2_, K‐I_2_, Mg‐I_2_, Al‐I_2_, and Zn‐I_2_ batteries, respectively. (f) Cycling stability at 10 A g^−1^ over 42,000 cycles. (g) Comparison of capacity and voltage between this work and the reported Zn‐based batteries (The specific capacity is calculated based on the iodine mass). (h) Cycling stability of the soft‐packaged zinc‐iodine battery with a high areal loading of 7.5 mg cm^−2^ at 0.2 A g^−1^ (Inset: the digital image shows a soft‐packaged zinc‐iodine cell). (i) Rate capability of zinc‐iodine battery with the ZSCCT‐2 electrolyte at a low temperature of ‐25°C.

The zinc‐iodine full batteries were fabricated to evaluate the influence of optimized electrolyte on the four‐electron transfer process of I^−^/I^0^/I^+^ (Figure S58). Single‐additive control experiments were conducted to verify the necessity of TES^−^ and Cl^−^ synergistic coordination for stabilizing I^+^ and enabling four‐electron conversion (Figures S59 and S60). The electrolyte containing only TES‐Na (ZST) delivers only a conventional two‐electron capacity of ∼240 mAh g^−1^ at 2 A g^−1^, without obvious I^+^/I^0^ redox activity, indicating that TES^−^ alone cannot effectively activate the I^+^/I° conversion. The electrolyte containing only ChCl (ZSCC) partially activates the I^+^/I° conversion, delivering ∼310 mAh g^−1^ at 2 A g^−1^ with an evident high‐voltage plateau (The capacity was calculated based on the mass of iodine in the cathode, which can be obtained from the thermogravimetric analysis (Figure S61)). However, the Cl^−^‐coordinated I^+^ species lack steric protection and remain vulnerable to hydrolysis, resulting in limited capacity and cycling stability. Only the dual‐additive ZSCCT‐2 electrolyte with coexisting Cl^−^ and TES^−^ enables near‐theoretical four‐electron capacity and stable cycling performance (Figure 6a), further confirming that the TES‐I‐Cl coordination structure plays the dominant role in stabilizing I^+^ and enabling reversible four‐electron iodine chemistry. According to the discharge profiles, the I^+^/I^0^/I^−^ conversion based on the ZSCCT‐2 electrolyte can deliver a high capacity of 420 mAh g^−1^ at 1 A g^−1^ and two distinct discharge voltage platforms (Figure 6a). Compared with the zinc‐iodine batteries with the 2 M ZS and ZSCC electrolytes, the battery with the ZSCCT‐2 electrolyte can accommodate larger energy density with notably higher capacity and voltage, which is attributed to the high activity and stability of the TES^−^‐containing coordination structures. CV measurements were carried out to examine the redox process of I^+^/I^0^/I^−^ in the various electrolytes. As shown in Figure 6b, CV curves of the zinc‐iodine battery with the ZSCCT‐2 electrolyte at various scan rates exhibit two pairs of oxidation and reduction peaks, which can be assigned to the reversible reactions of I^0^/I^−^ at low voltage and I^+^/I^0^ at high voltage, respectively. Specifically, the battery with the ZSCCT‐2 electrolyte displays a relatively larger response current than the cases of 2 M ZS and ZSCC electrolytes for all scan rates (Figure 6c and Figure S62), indicating the enhanced reactivity and redox kinetics of I^+^/I^0^/I^−^ conversion in the ZSCCT‐2 electrolyte. Moreover, the capacitive contributions from the non‐diffusion‐controlled processes at various scan rates were calculated (Figures S63 and S64). Compared with 2 M ZS and ZSCC electrolytes, the ZSCCT‐2 electrolyte consistently exhibits higher capacitive contributions across all scan rates. Notably, the proportion of the non‐diffusion‐controlled contribution increases from 32.2% to 55.5% as the scan rate increases. This enhanced capacitive behavior indicates faster kinetics and supports the superior rate capability of the battery [82].

Figure 6d compares the rate capabilities of zinc‐iodine batteries in the various electrolytes. As expected, the ZSCCT‐2 electrolyte endows the battery with high capacities at all current densities, exhibiting an excellent rate capability. Even at a high current density of 10 A g^−1^, the battery with the ZSCCT‐2 electrolyte possesses a high capacity of ∼260 mAh g^−1^. Moreover, the corresponding discharge curves of the battery with the ZSCCT‐2 electrolyte display two‐defined voltage plateaus (Figure S65), which is attributed to the advanced interfacial redox kinetics of iodine conversion (Figure 5b). For comparison, the rate capability of most previously reported works of other metal‐iodine batteries (MIBs) is summarized in Figure 6e. Our work based on the optimized electrolyte, endows the iodine chemistry with a high‐rate capability to date. Therefore, the ZSCCT‐2 electrolyte, relying on a four‐electron transfer of I^+^/I^0^/I^−^, effectively enhances the capacity compared to the MIBs. Compared with previously reported four‐electron zinc‐iodine batteries, the present work also achieves several distinct advances (Table S4). Besides, at a low current density of 0.5 A g^−1^ (Figures S66 and S67), the zinc‐iodine battery with the ZSCCT‐2 electrolyte exhibits two distinct discharge plateaus and delivers an initial discharge capacity of ∼437 mAh g^−1^ and retains 392 mAh g^−1^ after 100 cycles, demonstrating excellent reversibility and cycling stability. Moreover, the battery with the ZSCCT‐2 electrolyte exhibits a long‐term cycling life of over 12,000 cycles with a capacity retention of 78.8% at 5 A g^−1^, surpassing the values in other electrolytes (Figures S68–S70). Impressively, even at a higher current density of 10 A g^−1^, the battery with the optimized electrolyte still delivers long cycling stability with 70% capacity retention after 42,000 cycles (Figure 6f and Figure S71). The self‐discharge tests were carried out to evaluate the I^+^ hydrolysis (Figure S72). After 24 h of resting at open‐circuit, the zinc‐iodine battery using the ZSCCT‐2 electrolyte maintains a high open‐circuit voltage of ∼1.66 V, which is significantly higher than the low‐voltage plateau of the I^0^/I^−^ redox couple. This clearly indicates that the I^+^ species formed during charging remain effectively stabilized in the ZSCCT‐2 electrolyte, thereby enabling the high‐voltage I^0^/I^+^ redox reaction. In contrast, the battery with the 2 M ZS electrolyte rapidly drops to ∼1.30 V under the same conditions, confirming that only the I^0^/I^−^ reaction can be sustained, while the I^+^ species undergo severe hydrolysis or irreversible conversion. This conclusion is further corroborated by the discharged capacity after rest: the zinc‐iodine battery with the ZSCCT‐2 electrolyte delivers a high capacity of ∼377 mAh g^−1^, close to the theoretical four‐electron capacity, whereas the zinc‐iodine battery with the 2 M ZS cell only achieves a capacity of ∼178 mAh g^−1^, consistent with the limited two‐electron transfer process. These results provide direct electrochemical evidence that our steric‐hindrance strategy effectively suppresses I^+^ hydrolysis and promotes highly reversible I^0^/I^+^ conversion. To demonstrate the superiority of the ZSCCT‐2 electrolyte, we compared it with the generally applied electrode materials for zinc‐based batteries, such as multi‐electron transfer zinc‐iodine (dark gray) [42, 71, 83, 84, 85], conventional zinc‐iodine (purple) [86, 87], manganese‐based (dark cyan) [13, 88, 89], vanadium‐based (orange) [16, 90, 91], Prussian‐based (blue) [92, 93, 94], organic‐based (green) [21, 23, 95], and others (maroon) [96, 97]. Three indices of the capacity, voltage, and energy density are evaluated, as charted in Figure 6g. Overall, the zinc‐iodine battery with the optimized electrolyte combines cycling stability at high current densities, excellent rate capability, and low‐temperature stability, thereby demonstrating outstanding competitiveness among the zinc‐based batteries. More importantly, the optimizing strategy of the steric‐hindrance effect could also be extended to other zinc salt‐based aqueous electrolytes (Figures S73–S75), greatly enhancing its commercial value.

Finally, soft‐packaged zinc‐iodine batteries were assembled to further illustrate the feasibility of practical applications of the optimized electrolyte in iodine chemistry (Figure S76). Figure 6h presents the cycling performance of the soft‐packaged zinc‐iodine battery with a high areal loading of 7.5 mg cm^−2^ at 0.2 A g^−1^. The battery delivers a high capacity of 40.7 mAh and maintains a high‐capacity retention of 78.5% over 60 cycles. The relatively high‐areal loading and near‐unity N:P ratio further highlight the practical relevance of this battery. We further evaluated the low‐temperature adaptability of the optimized electrolyte. The differential scanning calorimetry (DSC) measurement shows that the ZSCCT‐2 electrolyte remains liquid down to approximately ‐39°C without freezing (Figure S77), which is attributed to the disruption of the hydrogen‐bond networks between water molecules by the dual additives. Figure 6i presents the rate performance of the zinc‐iodine battery with the ZSCCT‐2 electrolyte at ‐25°C. The battery shows superior rate performance from 2 to 10 A g^−1^. Encouragingly, the Zn||Zn cell and zinc‐iodine battery with the ZSCCT‐2 electrolyte achieve good cycling stability (Zn||Zn: 400 h; zinc‐iodine: 81.2% capacity retention over 400 cycles) (Figures S78 and S79). The excellent electrochemical performance of zinc‐iodine with the ZSCCT‐2 electrolyte can be associated with the following factors: First, the introduction of additive ions into ZnSO_4_‐based electrolyte significantly improves the ionic conductivity and reduces the interface resistance, endowing the cells with low polarization and fast redox kinetics. Second, the strong anchoring effect of Cl^−^ and TES^−^ on I^+^ can form stable coordination structures. More importantly, the steric‐hindrance effect of TES^−^ in this coordination structure effectively inhibits the I^+^ hydrolysis reaction. These features collectively contribute to the achievement of high‐voltage zinc‐iodine with four‐electron transfer, particularly for operation in extreme environments.

## 3 Conclusions

In summary, we have reported a steric coordination strategy on how the steric‐hindrance effect influences the reversibility and stability of I^+^ ions. Through a comprehensive investigation that combines advanced spectroscopic characterizations and simulations, this study addresses the critical challenge of unstable I^+^ species in the four‐electron transfer process by effectively shielding I^+^ species from nucleophilic attacks. Specifically, Cl^−^ ions promote and stabilize the I^0^/I^+^ conversion through coordination, while the steric‐hindrance effect provided by TES^−^ within the proposed coordination structure acts as a physical barrier to further protect I^+^ from hydrolysis, thereby facilitating highly reversible four‐electron iodine redox chemistry. Moreover, the introduction of dual additives disrupts the intrinsic H‐bonds of water molecules and forms a shielding layer near zinc, thereby suppressing the water‐parasitic reactions and promoting uniform zinc plating/stripping. As a result, the Zn||Zn symmetric cell using the optimized electrolyte achieves over 2 000 h of stable cycling at 5 mA cm^−2^. Meanwhile, the zinc‐iodine battery demonstrates reversible and stable four‐electron transfer iodine chemistry, including excellent durability over 42,000 cycles (capacity retention: ∼70%) and robust low‐temperature operation (∼81.2% capacity retention after 400 cycles at ‐25°C). These findings underscore the potential of tailored electrolyte engineering in overcoming intrinsic limitations of multielectron transfer in high‐voltage zinc‐iodine batteries.

## Conflicts of Interest

The authors declare no conflict of interest.

## Supporting information




**Supporting File**: adma73681‐sup‐0001‐SuppMat.docx.

## Data Availability

The data that support the findings of this study are available from the corresponding author upon reasonable request.
