# Supplementary material for: Steric Coordination Modulated Iodine Chemistry With Four‐Electron Conversion for Zinc‐Iodine Batteries
Source: Adv Mater. 2026 Jun 11;38(40):e73681. doi: 10.1002/adma.73681 (PMC13378215; doi:10.1002/adma.73681)
Supplement: Supplementary file 1 — Supporting File: adma73681‐sup‐0001‐SuppMat.docx. [file ADMA-38-e73681-s001.docx]

Supporting Information

**Experimental section**

**Materials.** Iodine (I_2_, 99.8%), iodine monochloride (ICl, 98%), zinc sulfate heptahydrate (ZnSO_4_ꞏ7H_2_O, 99.5%), zinc trifluoromethanesulfonate (Zn(OTf)_2_, 98%), and zinc chloride (ZnCl_2_, 99.95%) were purchased from Macklin. Choline chloride (ChCl, 99%), 2-[2-Hydroxy-1,1-bis(hydroxymethyl)ethylamino]ethanesulfonic acid sodium salt (C_6_H_14_NO_6_SNa, TES-Na, 99%), active carbon (Ac), N-methyl-2-pyrrolidone (NMP, 99.5%), sodium methanesulfonate (CH_3_NaO_3_S, Ms-Na, 98%), and Super P were obtained from Aladdin. Polyvinylidene fluoride (PVDF) and Zn foils (thickness: 30 μm, 99.9%) were purchased from Sigma-Aldrich.

**Preparation of electrolyte and electrode.** The blank electrolyte was composed of 2M ZnSO_4_ (labeled as 2M ZS). The optimized electrolytes composed of 2M ZnSO_4_, 5M ChCl, and different concentrations of TES-Na (0, 0.2, 0.5, and 1M) and were labeled as ZSCC, ZSCCT-1, ZSCCT-2, and ZSCCT-3, respectively. The control electrolyte contained 2M ZnSO_4_, and 0.5M TES-Na (labeled as ZST, with only TES-Na). The control electrolyte contained 2M ZnSO_4_, 5M ChCl, and 0.5M Ms-Na (labeled as ZSCCM). Zn and Cu foils (thickness: 30 μm) were cut into small disks (diameter: 12 mm) and then cleaned with deionized water and ethanol in sequence before use. The I_2_@Ac cathodes were fabricated using a melt-diffusion method. Typically, Ac and iodine (in a mass ratio of 1:2) were thoroughly mixed by hand grinding for about 20 mins. The mixture was sealed in a glass bottle and heated at 90 °C for 8 h. Then, I_2_@Ac composite, Super P, and PVDF binder in a weight ratio of 8:1:1 was mixed in NMP solvent. Subsequently, the slurry was uniformly coated on a carbon cloth (CC) and dried at 40 °C for 24 h. The areal mass loading of iodine in the cathodes was around 2.0 mg cm^-2^.

**Electrochemical measurements.** The Coulombic efficiency data were obtained from the Zn||Cu cells with a cutoff voltage of 0.5 V. The Zn plating/stripping behaviors, rate capability, electric double layer (EDL), and cycling performance were evaluated by Zn||Zn cells. All the cells were assembled into coin-type 2032 cells, and the electrolyte volume for each cell was about 100 μL. The commercial glass fiber (Whatman, GF/A) was used as a separator. The linear sweep voltammetry (LSV) curves were investigated in the three-electrode system (working electrode: Ti foil, counter electrode: Pt foil, and reference electrode: Ag/AgCl). Galvanostatic discharge/charge measurements of zinc-iodine batteries were conducted in a voltage range of 0.6-1.85 V (*vs*. Zn^2+^/Zn) on a Neware testing system. Cyclic voltammetry (CV, voltage range: 0.6-1.85 V (*vs*. Zn^2+^/Zn)) and electrochemical impedance spectroscopy (EIS, frequency range: 100 kHz to 10 mHz) curves were recorded with an amplitude of 5 mV from a CHI660E electrochemical workstation. All cells were rested for at least 24 h before electrochemical tests. Distribution of relaxation time (DRT) provides a time-domain analytical tool for electrochemical impedance spectroscopy that does not rely on prior knowledge of the research object (i.e., no equivalent circuit is required), allowing for the separation and analysis of highly overlapping physical and chemical processes in EIS data. For the DRT calculation, we used the open MATLAB code shared online by Prof. Francesco Ciucci.[^1^](#_ENREF_1) The DRT method systematically separates the electrochemical process by their distinct time constants (*τ*), thereby enabling direct characterization of timescale distributions through this temporal resolution mechanism. The DRT mathematical transformation was optimized by the Tikhonov regularization method.[^2^](#_ENREF_2)

**Material characterizations.** The scanning electron microscope (SEM, Tescan MIRA) and 3D laser scanning confocal microscopy (LSCM, KEYENCE VK-X200) were employed to investigate the morphologies and structures of the electrodes. Fourier-transformed infrared spectra (FTIR) were measured on a Perkin Spectrum 100 instrument. Raman spectra were obtained using a 532 nm excitation laser on a confocal Raman microscope (InVia Reflex). X-ray photoelectron spectroscopy (XPS) spectra were collected with a Thermo Fisher Scientific Nexsa instrument. A nuclear magnetic resonance (NMR) spectrometer (Bruker Avance NEO 600, 600 M) was applied to detect the ^1^H spectra for the various electrolytes. The 2D low-field nuclear magnetic resonance (LF-NMR, NMI20-040V-l) spectra were applied to provide relaxation dynamics information for water molecules. The UV-vis spectra were collected on an ultraviolet-visible (UV-vis, Agilent Cary 300 Conc) spectroscopy. The electrolyte contact angles were recorded from an SDC-350 contact angle measuring system. Thermogravimetric analysis (TGA) was performed on TGA 4000 with a heating rate of 5 °C min^-1^ from room temperature to 500 °C under a nitrogen atmosphere. The viscosity measurements of the various electrolytes were carried out on Brookfield DV-Ⅱ+ Pro at 25 °C. Phase and glass transitions were conducted using a differential scanning calorimetry (DSC, TA DSC25).

**I^+^ coordination structure characterizations.** To experimentally validate the coordination environment of I^+^ ion, a series of 1M aqueous solutions was prepared with ICl as the I^+^ source. The I^+^ source solution was prepared by slowly dissolving 324 mg of solid ICl in 2 mL of ultrapure water to obtain a 1M ICl solution. 278 mg of ChCl or 278 mg of ChCl and 458 mg of TES-Na were added to the above ICl solution, and the mixture was stirred thoroughly to afford the ICl+ChCl and ternary ICl+ChCl+TES systems for evaluating the I⁺ coordination structures. As a control, a 1M TES-Na solution was prepared by dissolving 458 mg of TES-Na in 2 mL of ultrapure water. The evolution of the I^+^ coordination environment across these solutions was interrogated by the FTIR and Raman spectroscopies.

**Computational methods.** All first-principles calculations based on density functional theory (DFT) were performed using the DMol3 module.[^3^](#_ENREF_3) The exchange-correlation energy was represented within the generalized gradient approximation (GGA) using the Perdew-Burke-Ernzerhof (PBE) functional, which provides an accurate description of electronic structure and bonding interactions in the studied systems. A global orbital cut-off radius of 4.4 Å was applied to ensure a balanced trade-off between computational efficiency and accuracy. Double numerical plus polarization (DNP) basis sets were employed for all atoms to account for polarization effects in the electronic structure calculations. The convergence criteria for all geometry optimizations were set as follows: a self-consistent field (SCF) energy threshold of 1.0×10^-5^ Ha, a maximum displacement of 5.0×10^-3^ Å, and a maximum force of 2.0×10^-3^ Ha Å^-1^. For surface calculations, a five-layer unit cell was adopted with the fixed bottom two layers. The DFT models employ periodic boundary conditions to approximate the extended Zn_[002]_ structure of the various ions or molecules on its surface. All DFT calculations employed periodic boundary conditions with a Monkhorst-Pack k-point grid of [2×2×1] for Brillouin zone integration. A 15 Å vacuum layer in the *z*-direction of the structures was applied to avoid the interactions between the periodic structures. All transition state search calculations were performed using the DMol3 module using the Complete LST/QST protocol. To account for the influence of the aqueous environment, an implicit solvation model was applied during the transition state search calculations. The conductor-like screening model (COSMO) was employed with a dielectric constant of 78.54 to represent bulk water.[^4^](#_ENREF_4)

The structural and dynamical properties of the electrolyte systems were analyzed through molecular dynamics (MD) simulations using the Forcite package with COMPASS force field (The detailed model systems are listed in Table S2). Electrolyte compositions (e.g., 2M ZS, ZSCC, ZSCCT-2) were modeled in cubic simulation boxes with >1500 atoms to ensure statistical reliability. Initial configurations were generated using the Amorphous Cell module with randomized molecular packing. The Coulombic and van der Waals interactions were calculated utilizing both the Ewald method and atom-based method with an accuracy of 1.0×10^-3^ kcal mol^-1^ and employing a cut-off distance of 12.5 Å. The constant temperature and pressure (NPT) process was initially employed at 300 K and 1 atm for 100 ps to equilibrate the electrolyte structures. Later, the simulation data were collected by running the equilibrated simulations with a constant volume and temperature (NVT) process at 300 K for 100 ps. H-bond networks were quantified using radial distribution functions (RDFs) and angular criteria (donor-acceptor distance < 3.5 Å, angle > 120°).


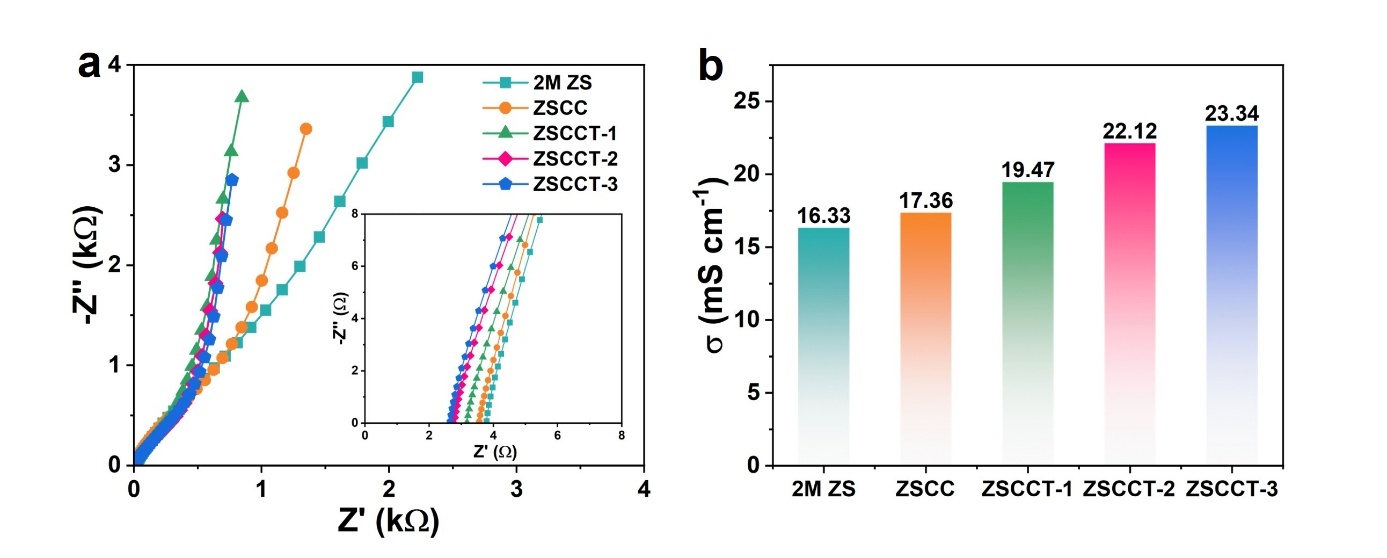


**Figure S1.** (a) Nyquist impedance plots and (b) ion conductivities of the various electrolytes.

**
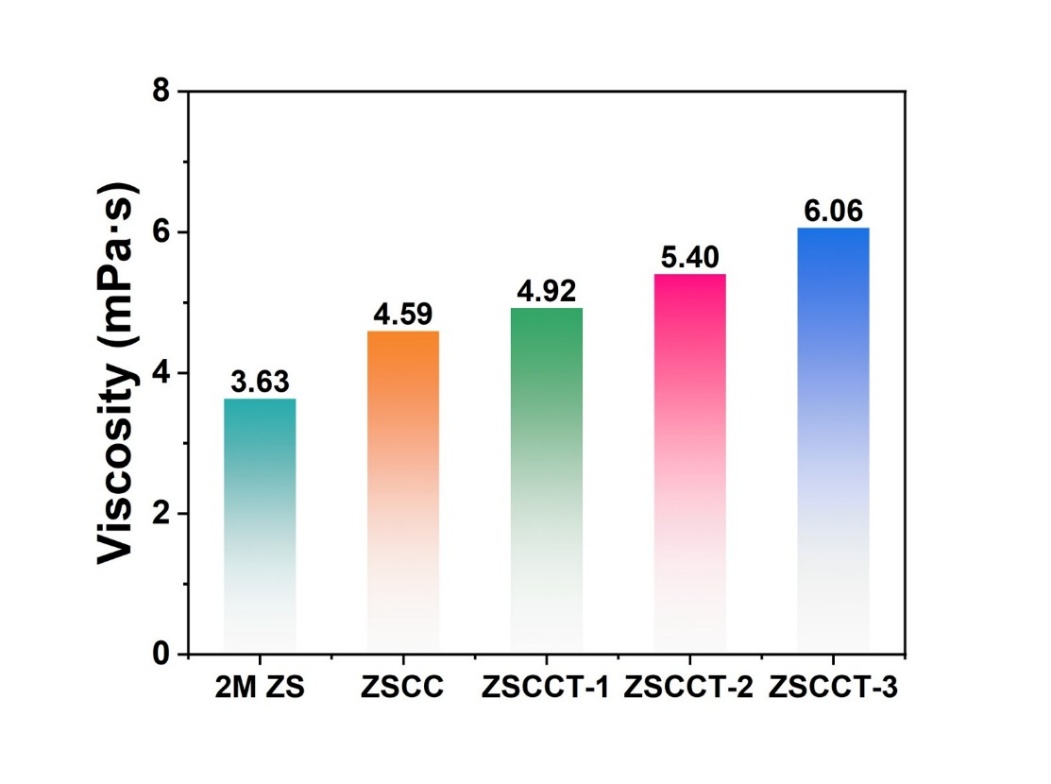
**

**Figure S2.** The viscosity values of the various electrolytes.


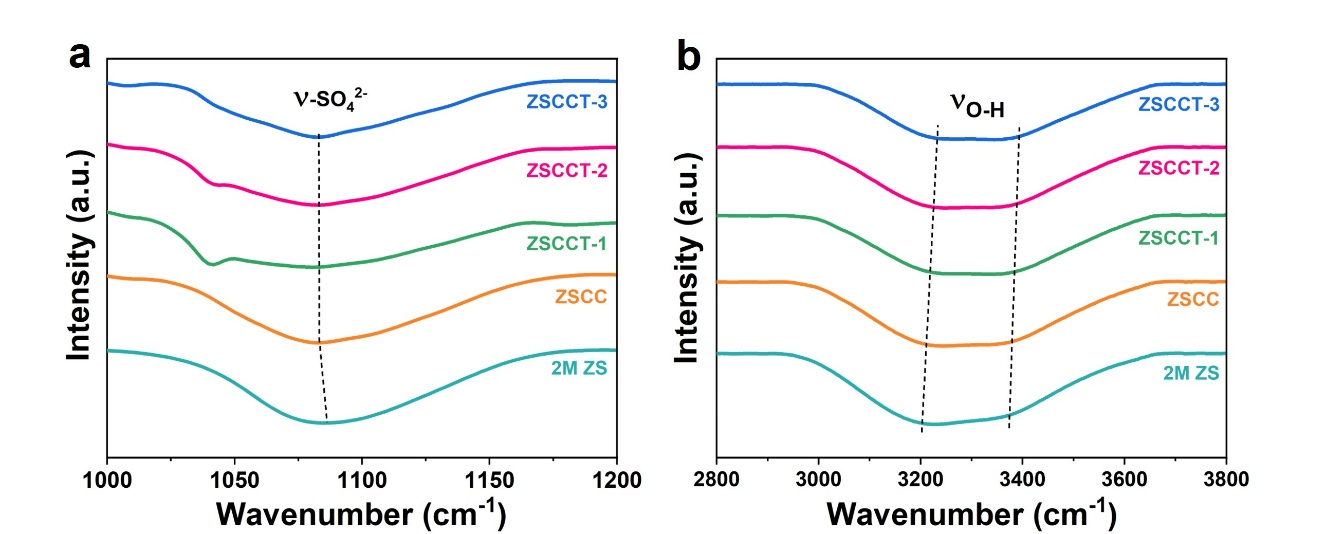


**Figure S3.** FTIR spectra of the various electrolytes.

**
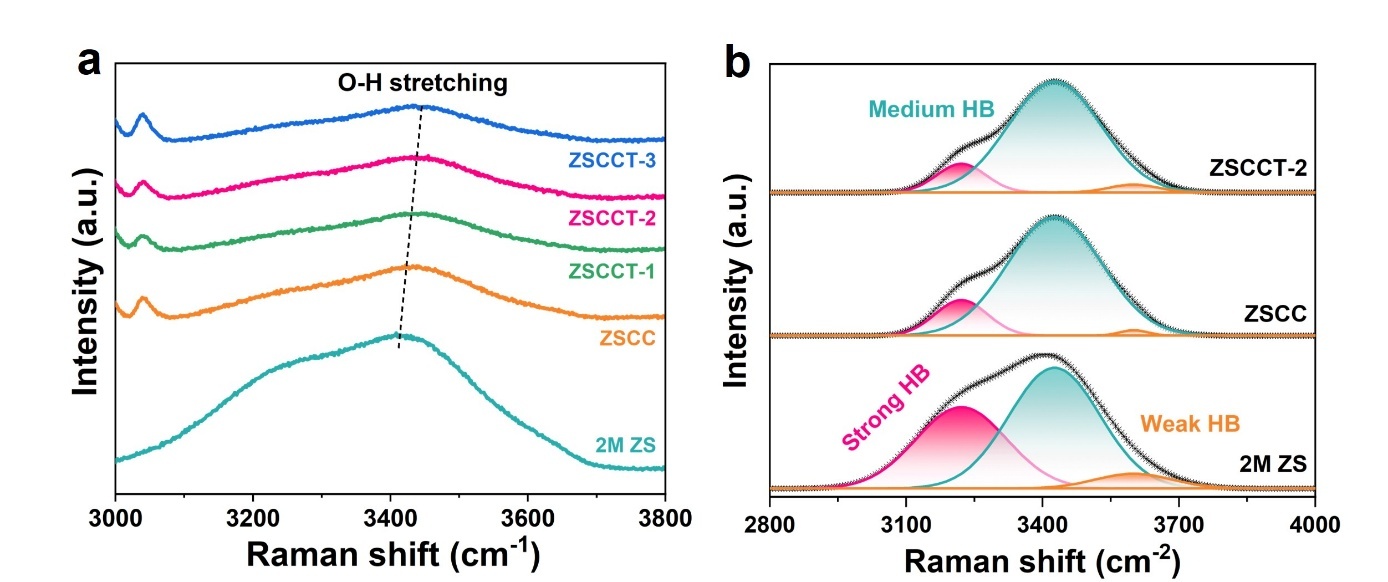
**

**Figure S4.** (a) Raman spectra of the various electrolytes. (b) Fitted Raman spectra of the various electrolytes in the region of O-H stretching.

**
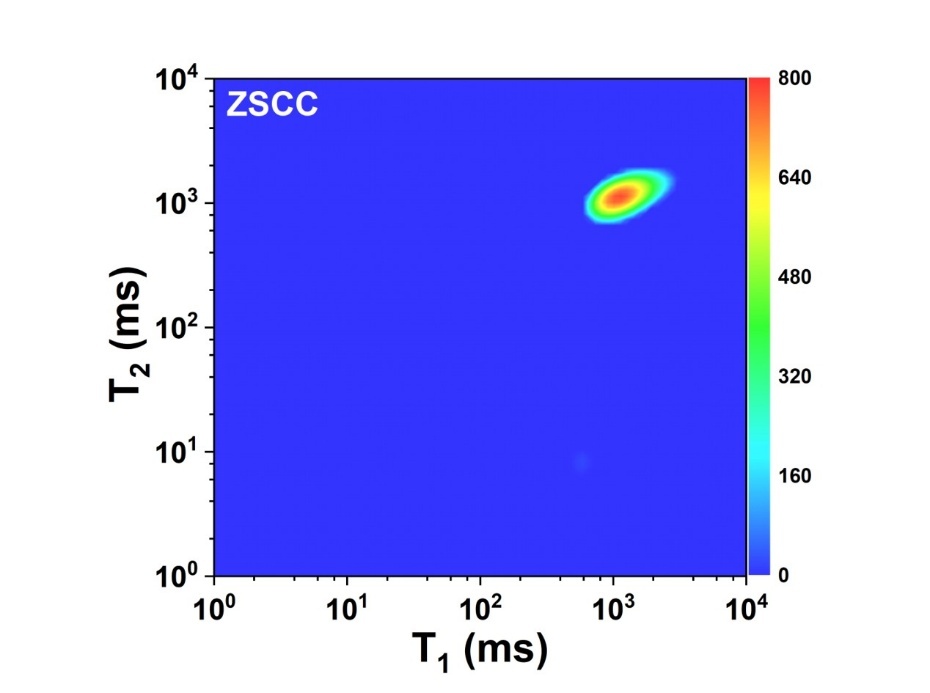
**

**Figure S5.** 2D LF-NMR T_1_-T_2_ relaxation spectrum of the ZSCC electrolyte.

**
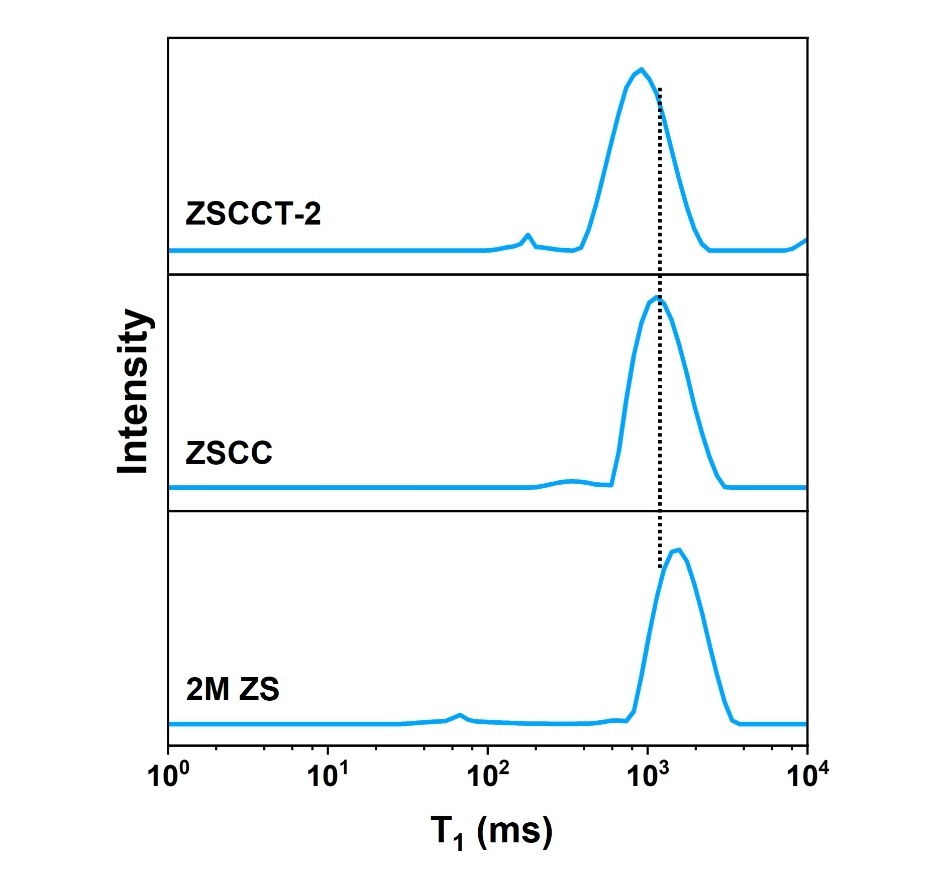
**

**Figure S6.** T_1_ relaxation spectra of the various electrolytes.

**
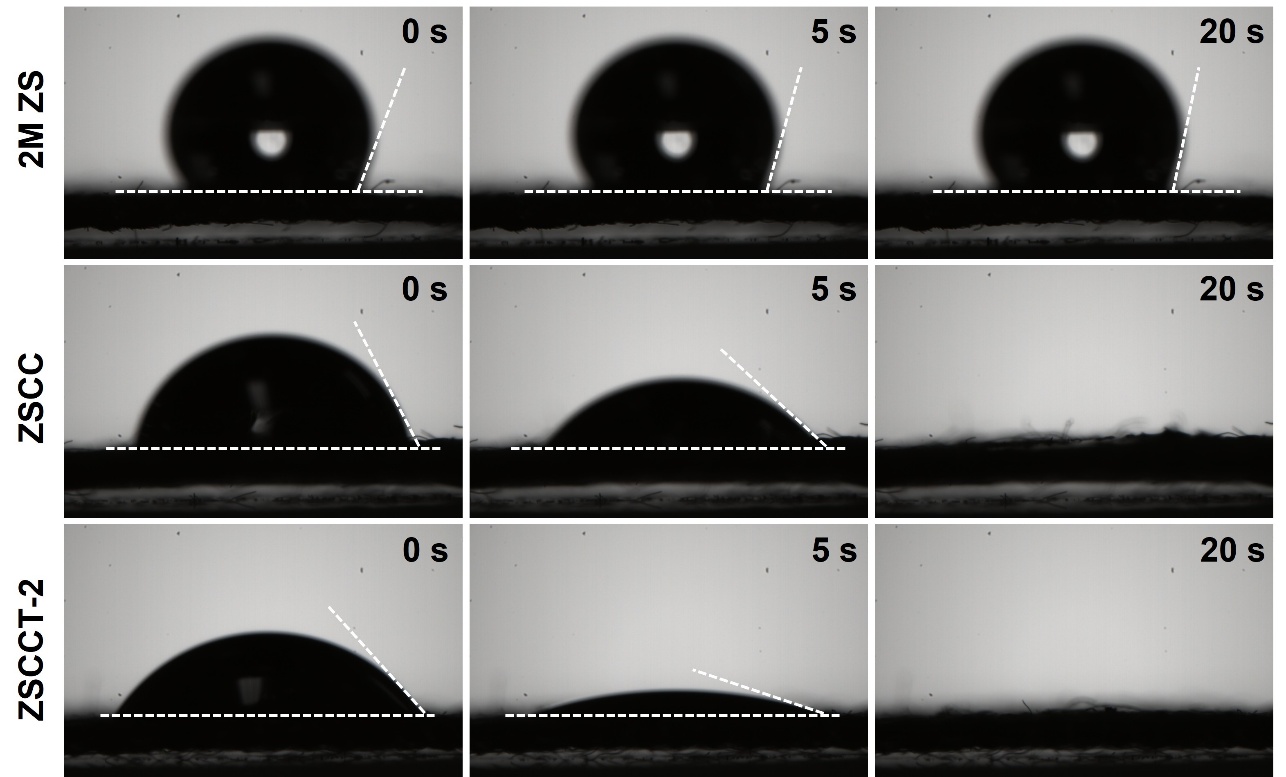
**

**Figure S7.** Optical images of the contact angles for various electrolytes on cathode surfaces after different times.

**
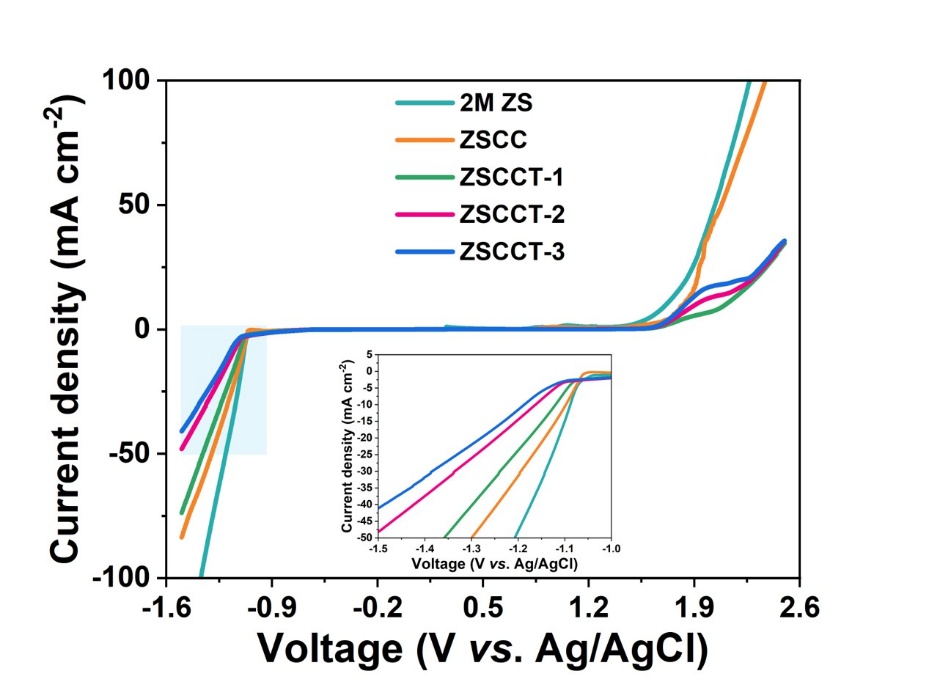
**

**Figure S8.** Linear sweep voltammetry (LSV) curves of the various electrolytes.

**
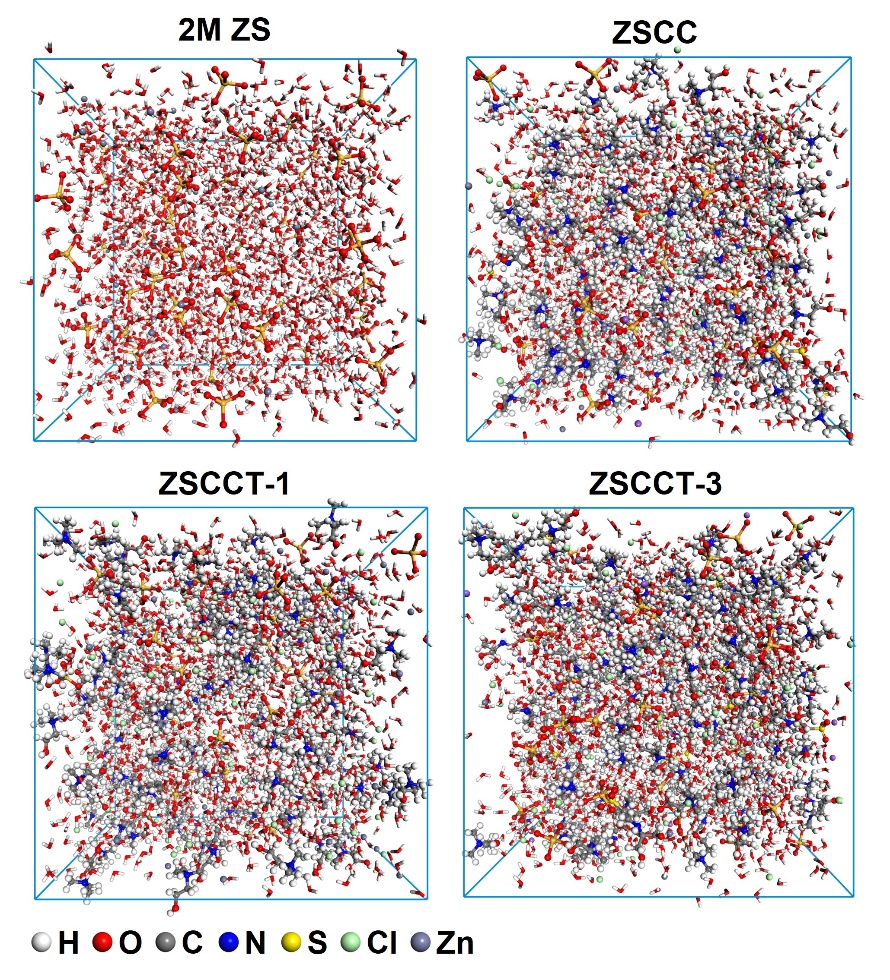
**

**Figure S9.** MD simulation snapshots of the various electrolytes.

**
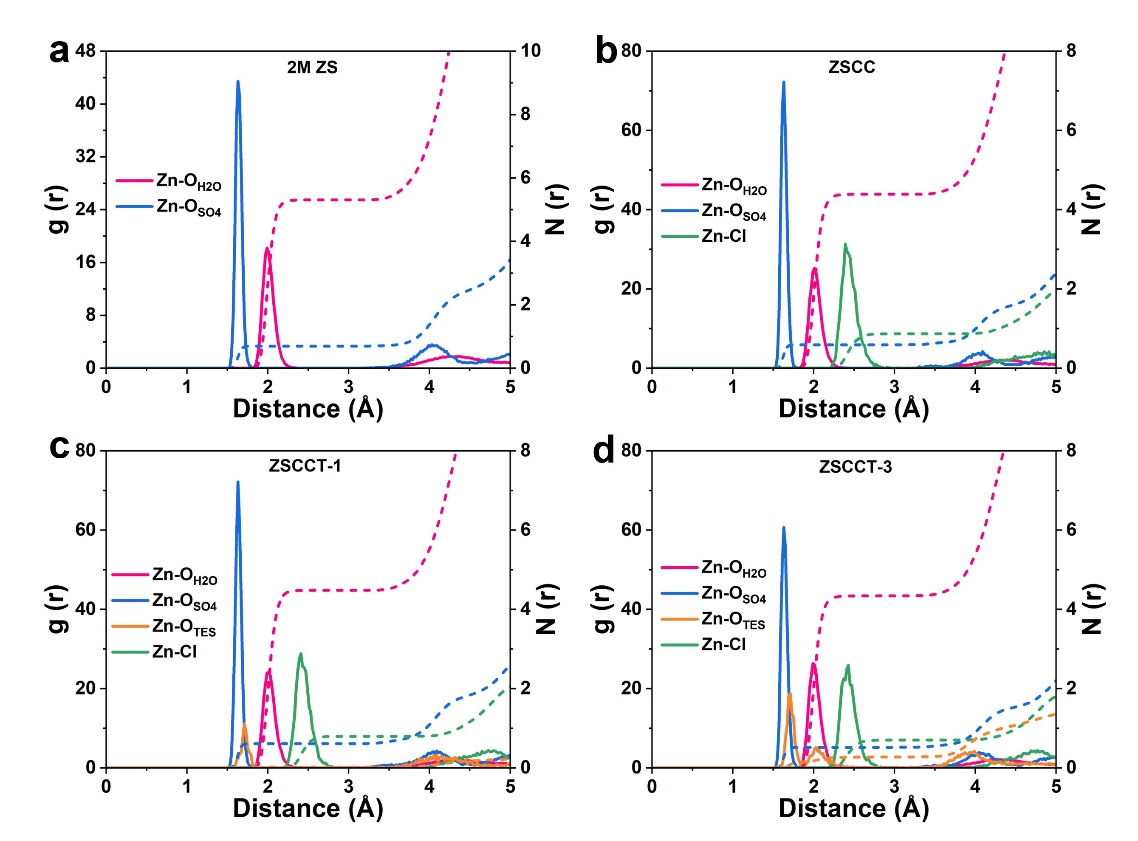
**

**Figure S10.** RDFs and the corresponding average coordination number of the various electrolytes.

**
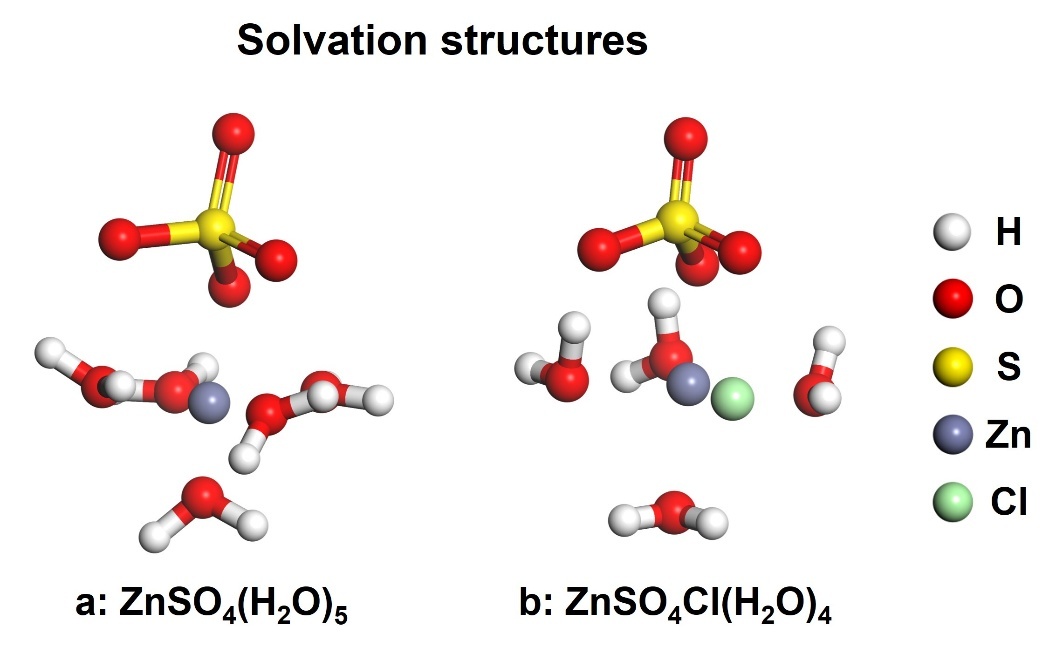
**

**Figure S11.** Solvation structures of ZnSO_4_(H_2_O)_5_ in the 2M ZS electrolyte and ZnSO_4_Cl(H_2_O)_4_ in the ZSCC, ZSCCT-1, and ZSCCT-2 electrolytes.

**
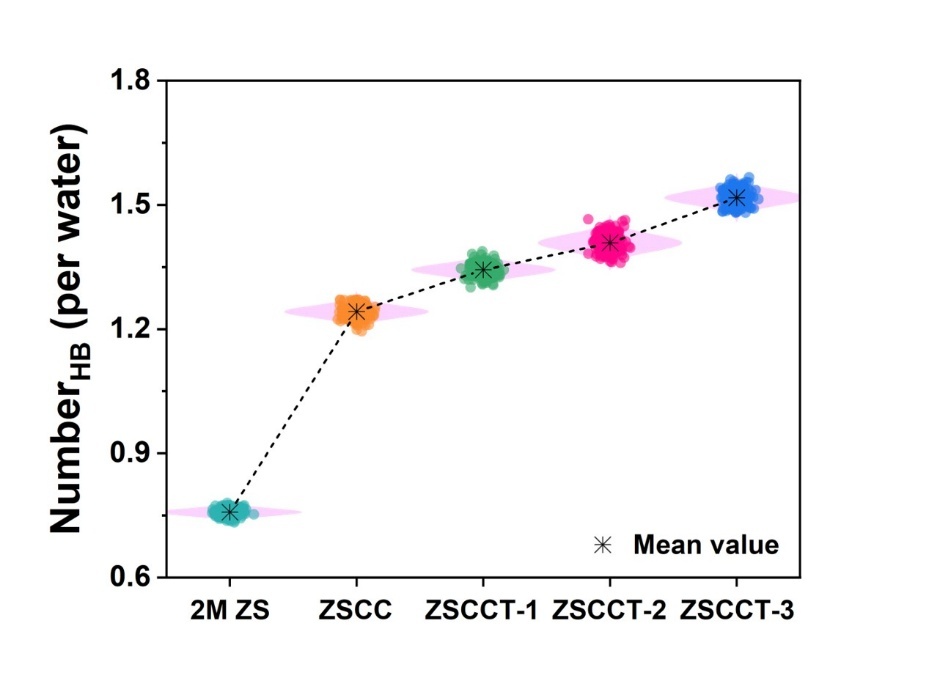
**

**Figure S12.** Mean values of the H-bonds between the additive ions and water molecules.

**
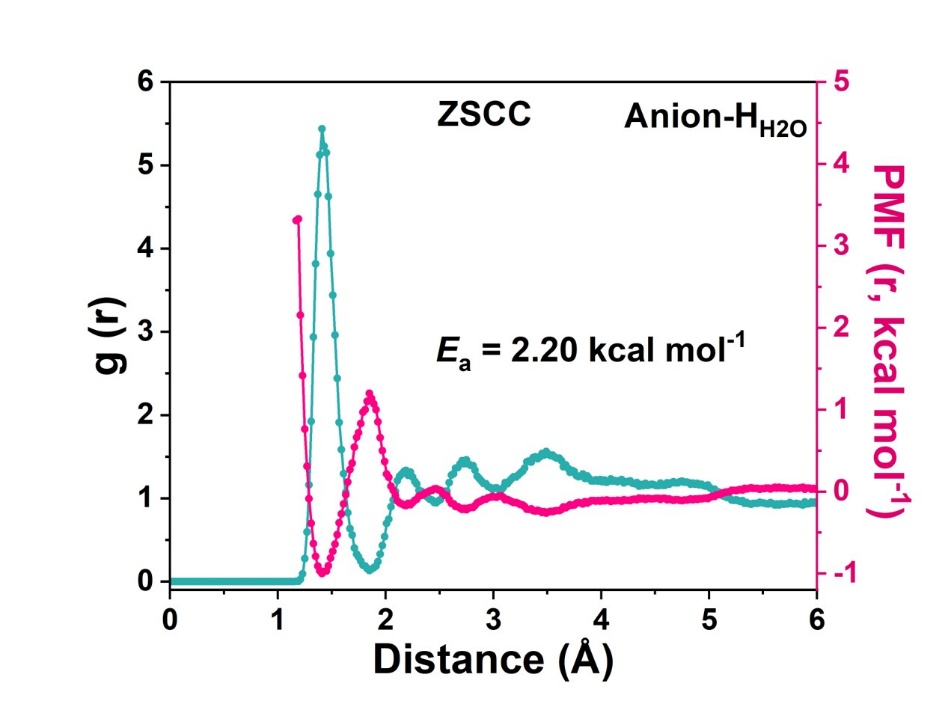
**

**Figure S13.** RDFs and PMFs of anion-H_H2O_ atom pairs in the ZSCC electrolyte.

**
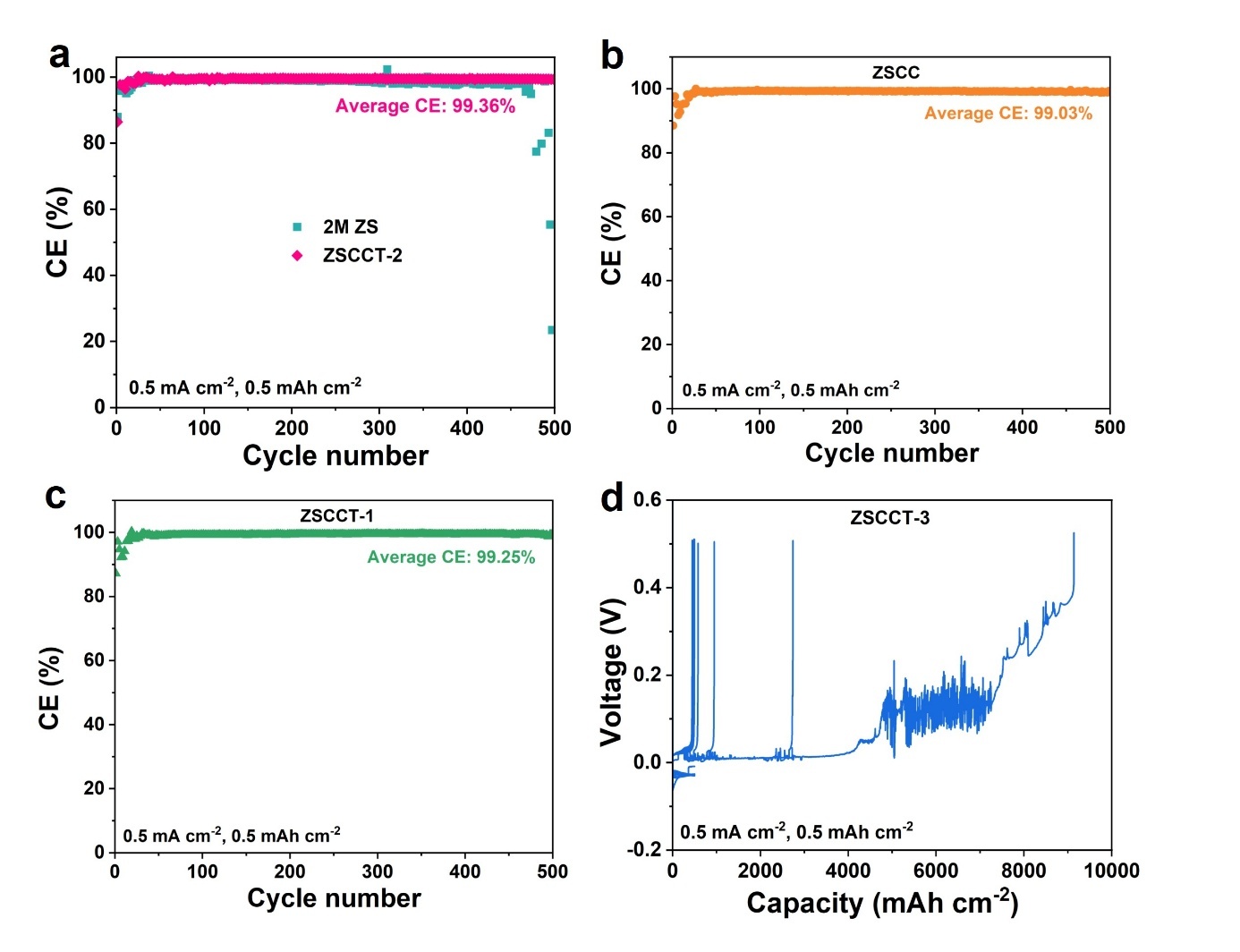
**

**Figure S14.** CE measurements at 0.5 mA cm^-2^, 0.5 mAh cm^-2^ in the various electrolytes. (a) 2M ZS and ZSCCT-2, (b) ZSCC, (c) ZSCCT-1, and (d) ZSCCT-3.

**
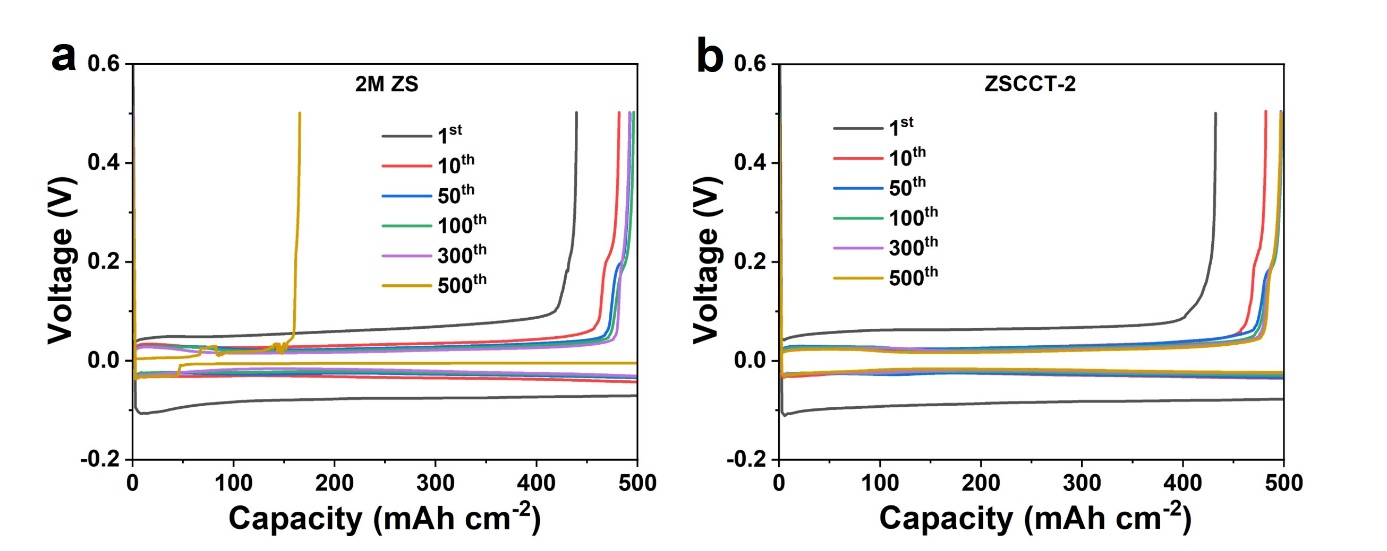
**

**Figure S15.** Voltage-capacity curves at 0.5 mA cm^-2^, 0.5 mAh cm^-2^ after different cycles in the various electrolytes. (a) 2M ZS and (b) ZSCCT-2.

**
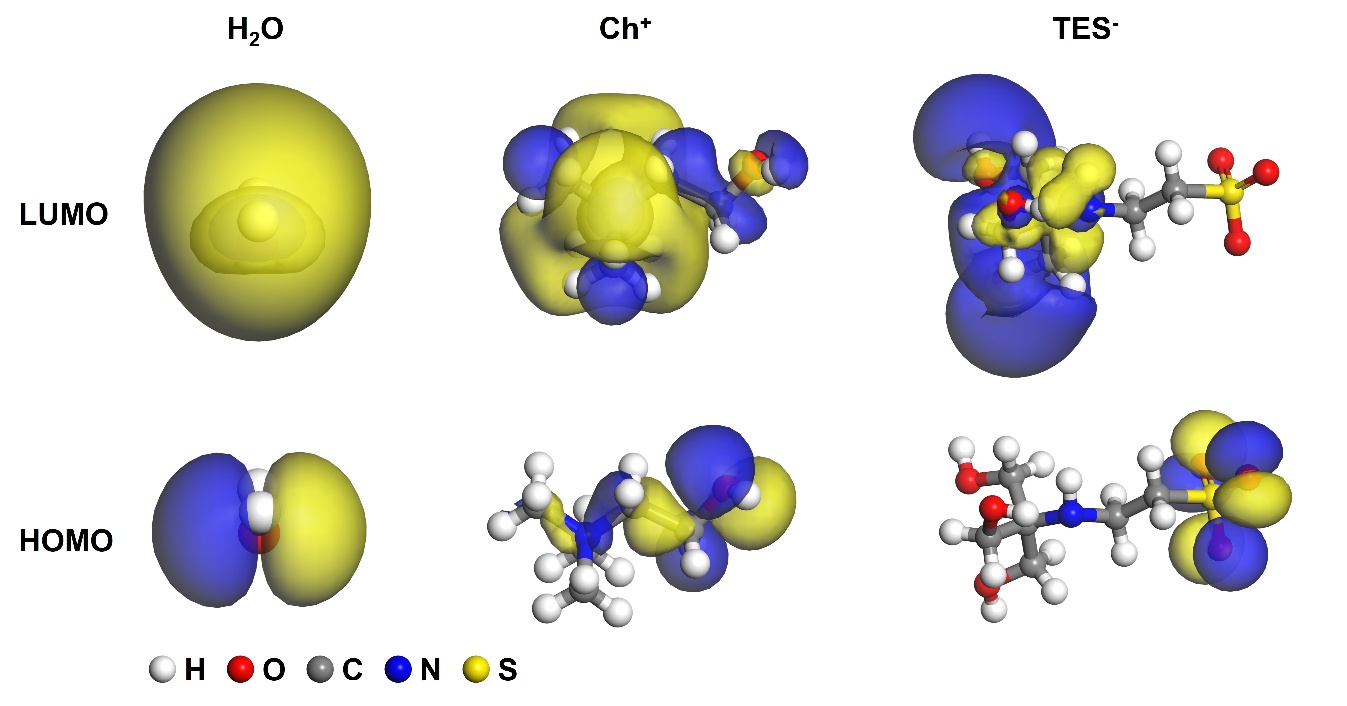
**

**Figure S16.** LUMO and HOMO of the different molecules or ions.

**
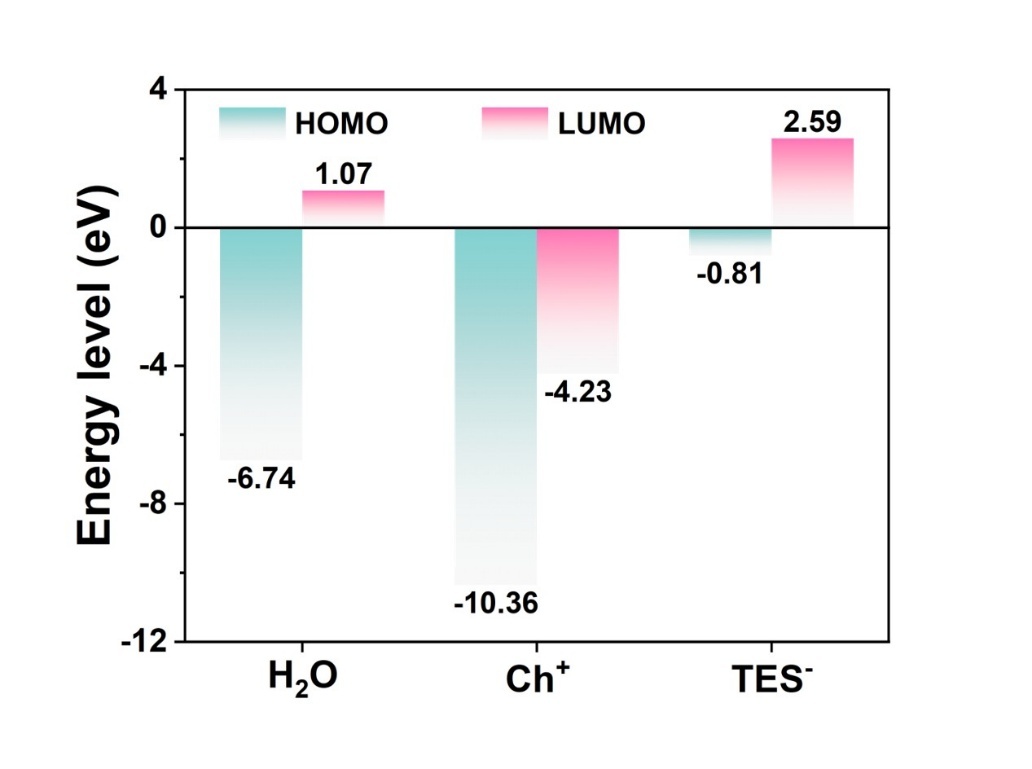
**

**Figure S17.** Frontier molecular orbital energy levels of the different molecules or ions.

**
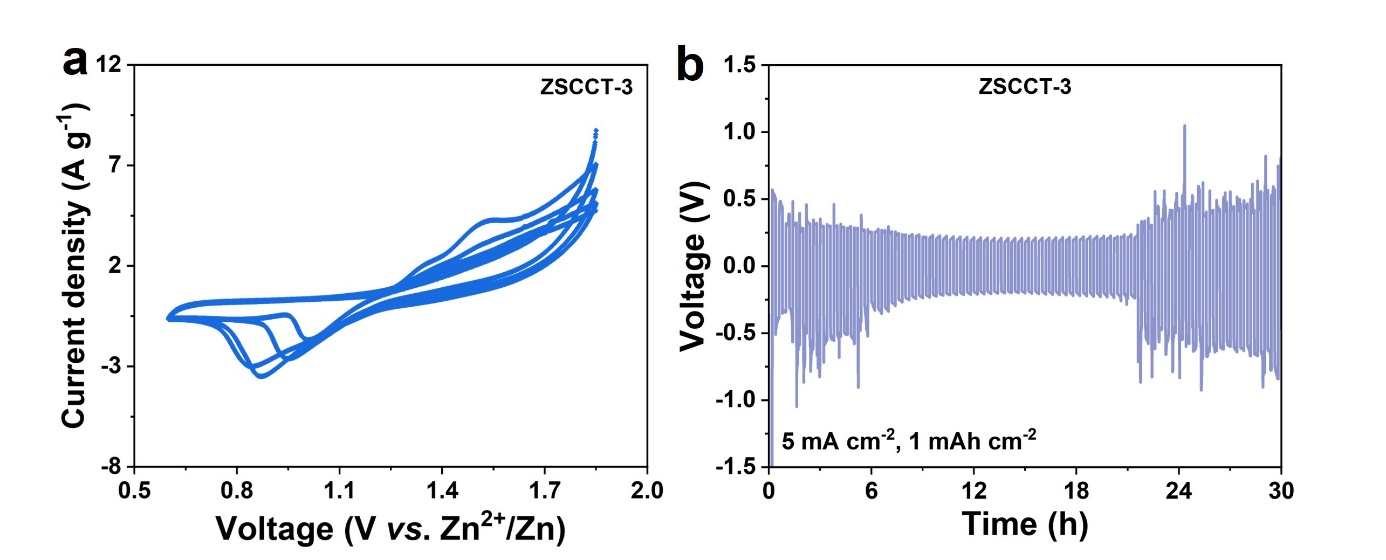
**

**Figure S18.** a) CV curves of zinc-iodine full battery with the ZSCCT-3 electrolyte. b) Cycling performance of Zn||Zn cell with ZSCCT-3 electrolyte at 5 mA cm^-2^, 1 mAh cm^-2^.

**
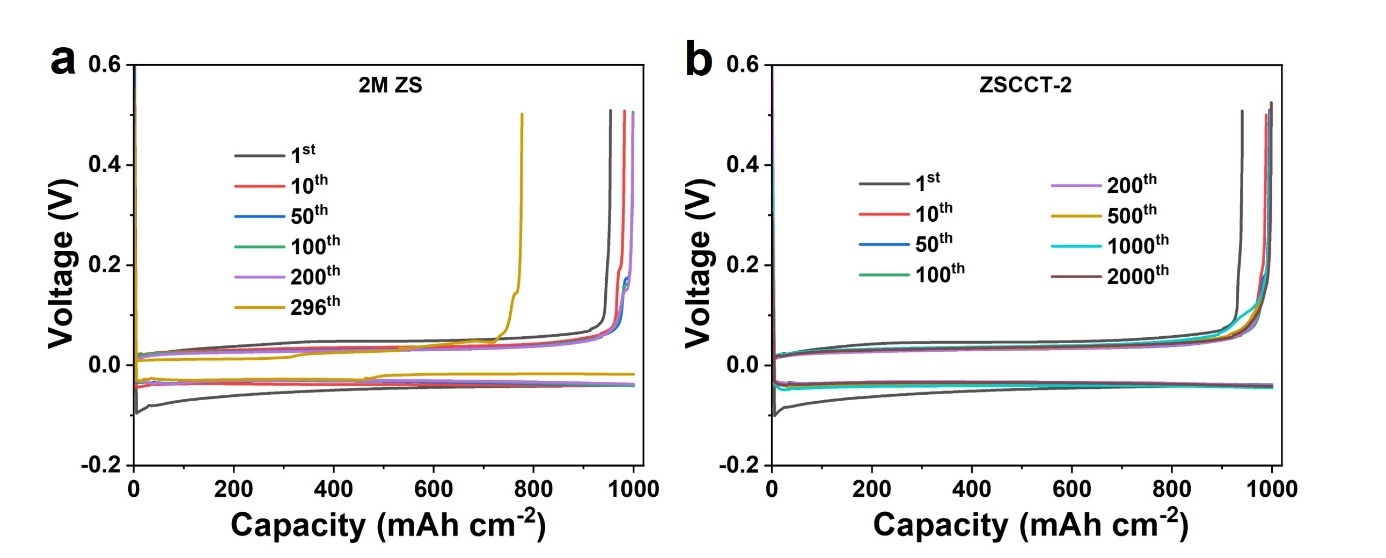
**

**Figure S19.** Voltage-capacity curves at 3 mA cm^-2^, 1 mAh cm^-2^ after different cycles in the various electrolytes. (a) 2M ZS and (b) ZSCCT-2.


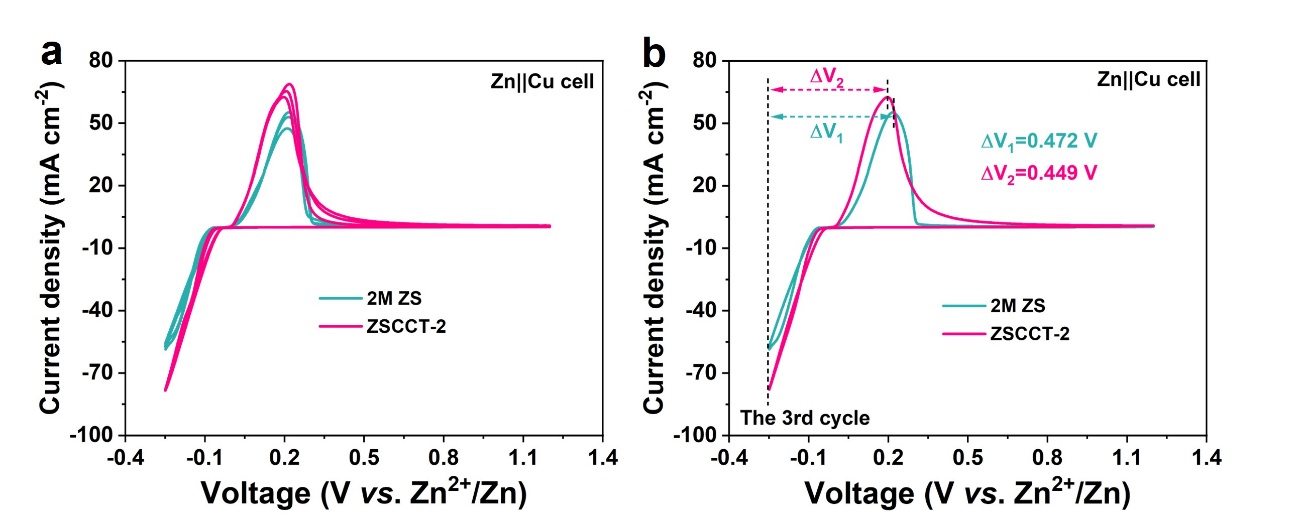


**Figure S20.** CV curves of Zn‖Cu cells at 10 mV s^-1^ in the various electrolytes.

**
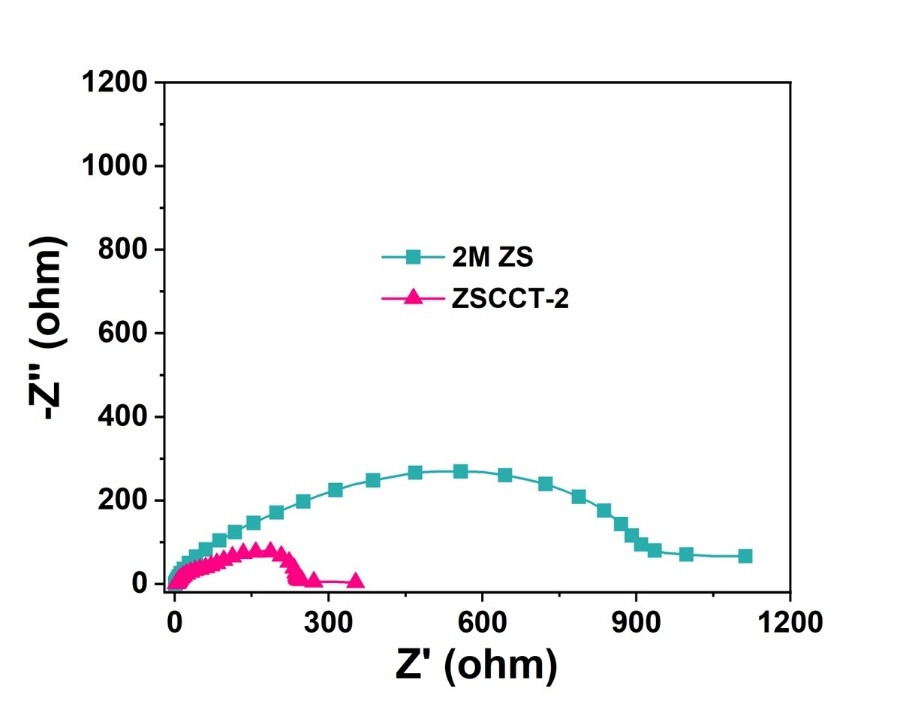
**

**Figure S21.** Nyquist impedance plots of Zn‖Zn symmetric cells after 3 cycles in the various electrolytes.


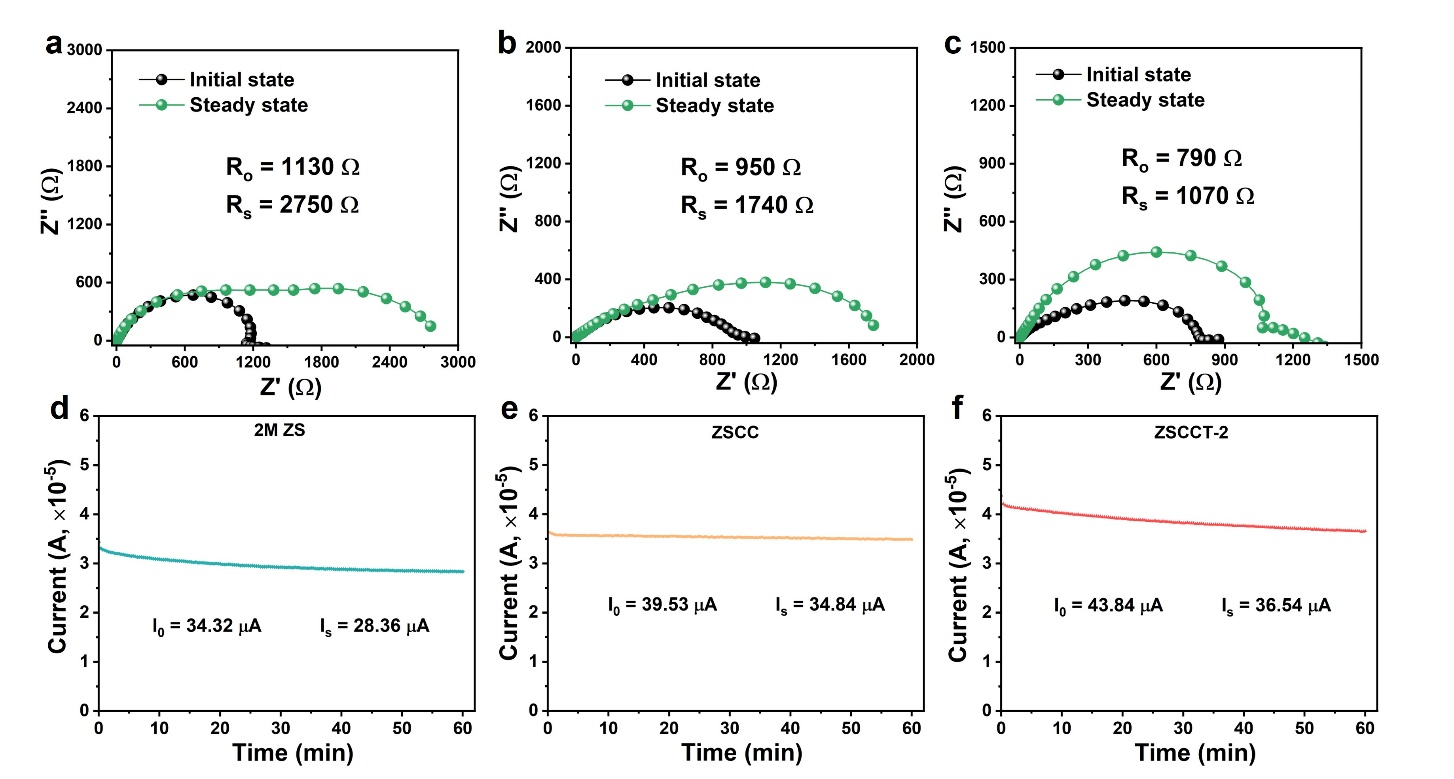


**Figure S22.** a-c) Nyquist impedance plots of Zn‖Zn symmetric cells before and after polarization in the various electrolytes. d-f) Current-time plots of Zn‖Zn symmetric cells in the various electrolytes after polarization at a constant potential (10 mV) for 60 min.


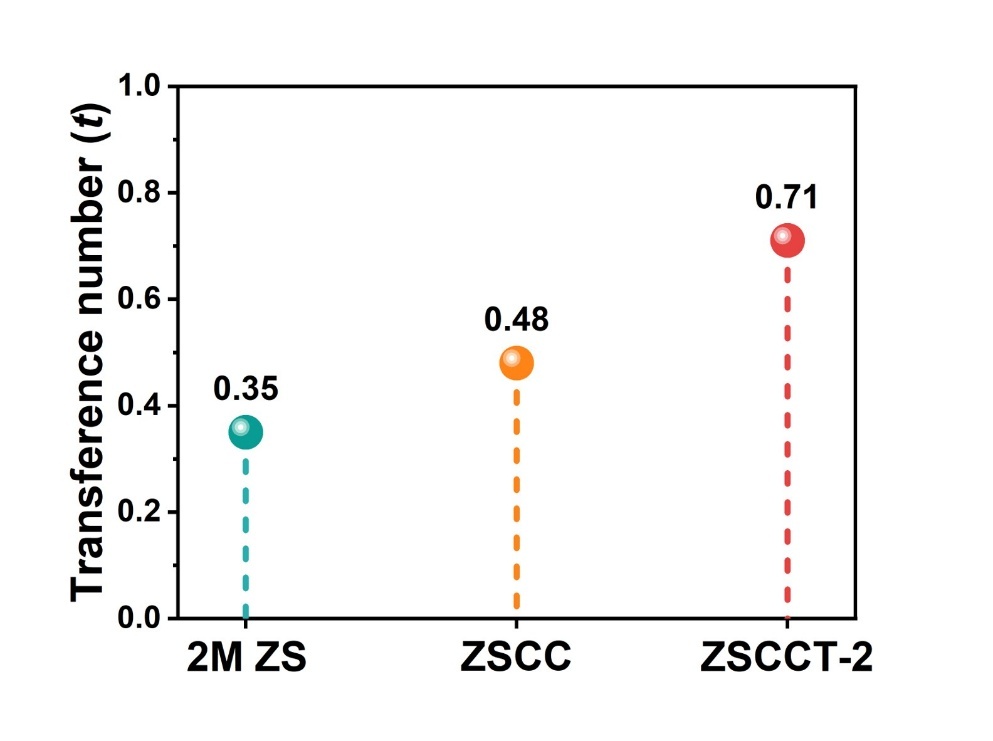


**Figure S23.** The calculated Zn^2+^ transference number in the various electrolytes.

The increase in Zn^2+^ transference number from 0.35 to 0.71 upon sequential addition of 5M ChCl and 0.5M TES-Na is primarily due to: 1) partial replacement of H_2_O in the Zn^2+^ solvation shell by Cl^-^, reducing the hydrodynamic radius of Zn^2+^; 2) disruption of the hydrogen-bond network of water, lowering water activity; 3) formation of a dehydrated electric double layer that minimizes solvent drag; and 4) maintenance of a favorable ionic conductivity without direct Zn^2+^ coordination by bulky TES^-^ ions at the optimal concentration.

**
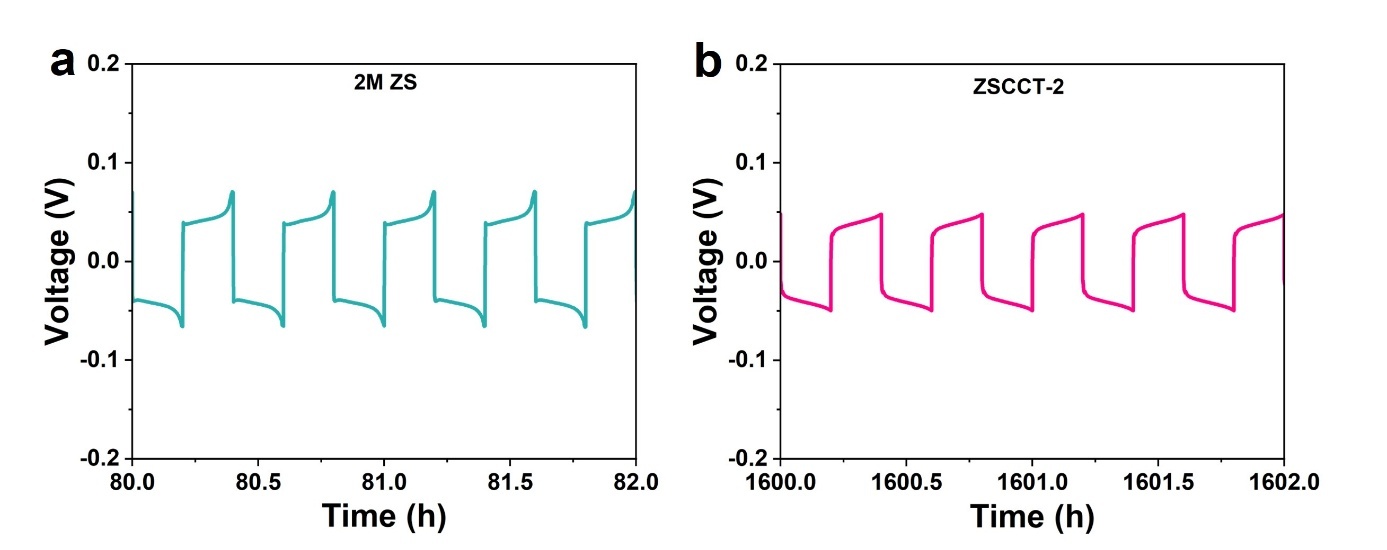
**

**Figure S24.** Enlarged voltage-time curves in Figure 3c at different cycles in the various electrolytes. a) 2M ZS and b) ZSCCT-2.

**
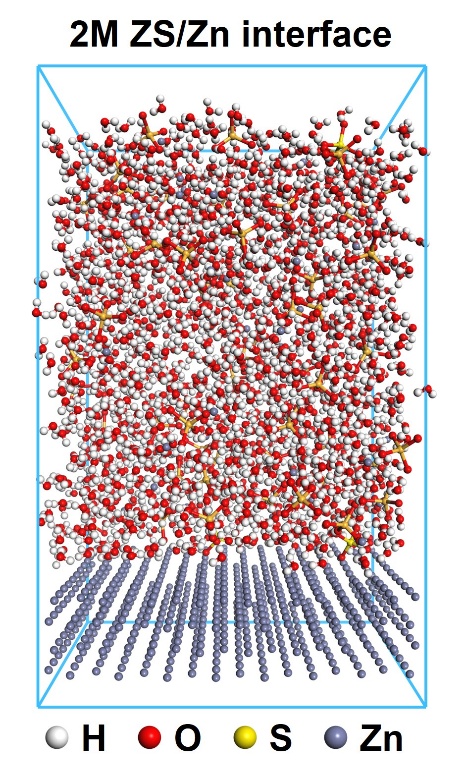
**

**Figure S25.** Surface energy for metallic Zn when exposed in the 2M ZS electrolyte.

**
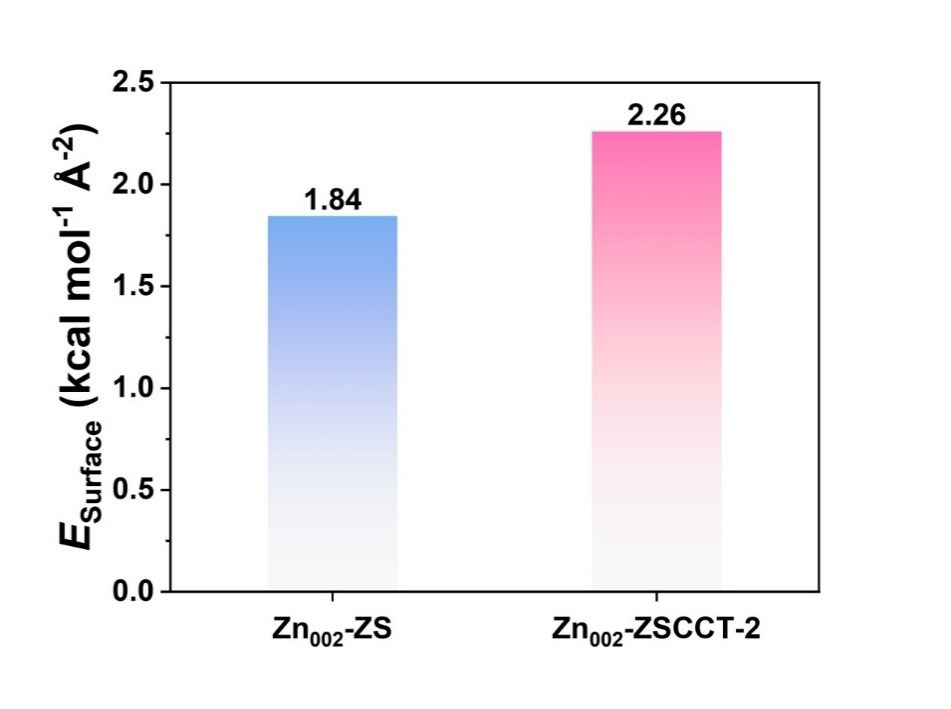
**

**Figure S26.** Values of surface energy for metallic Zn when exposed in the various electrolytes.

**
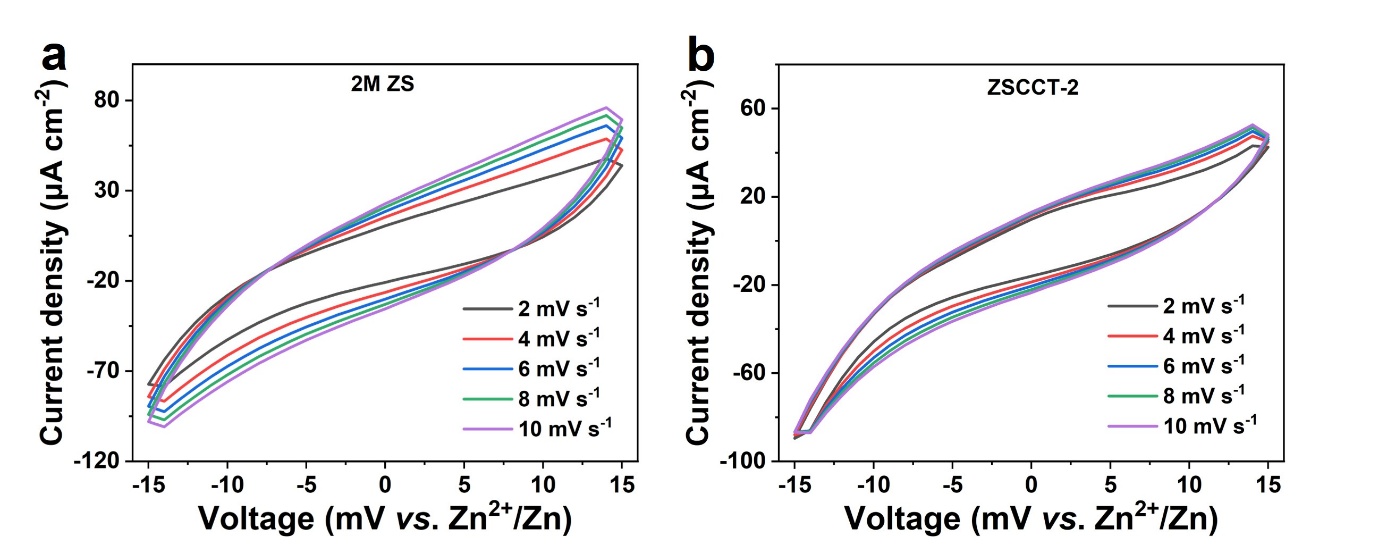
**

**Figure S27.** EDL measurements for Zn foils in the various electrolytes. (a) 2M ZS and (b) ZSCCT-2.

The capacitance (*C*) is determined by the linear relationship between capacitive current (*i*_c_) and scan rate (*v*), which can be achieved from the slope of the current-voltage curves. Therefore, the *C* could be calculated by the following equation:

$C=\frac{i_{c}}{v}$ (1)

where *i*_c_=(*i*_0v+_-*i*_0v-_)/2, meaning the half value of current difference during positive and negative scans at 0 V.

**
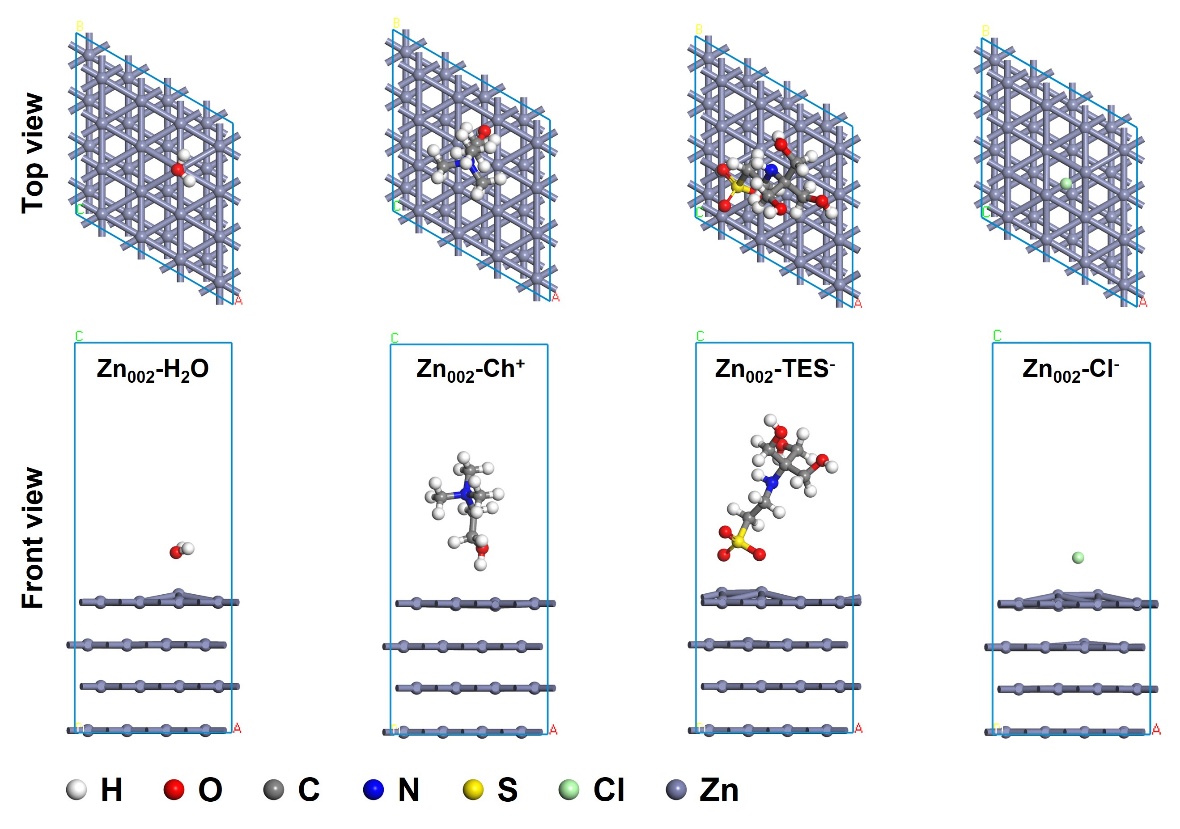
**

**Figure S28.** Surface adsorption structures of H_2_O, Ch^+^, TES^-^, and Cl^-^ on Zn_[002]_ plane.

**
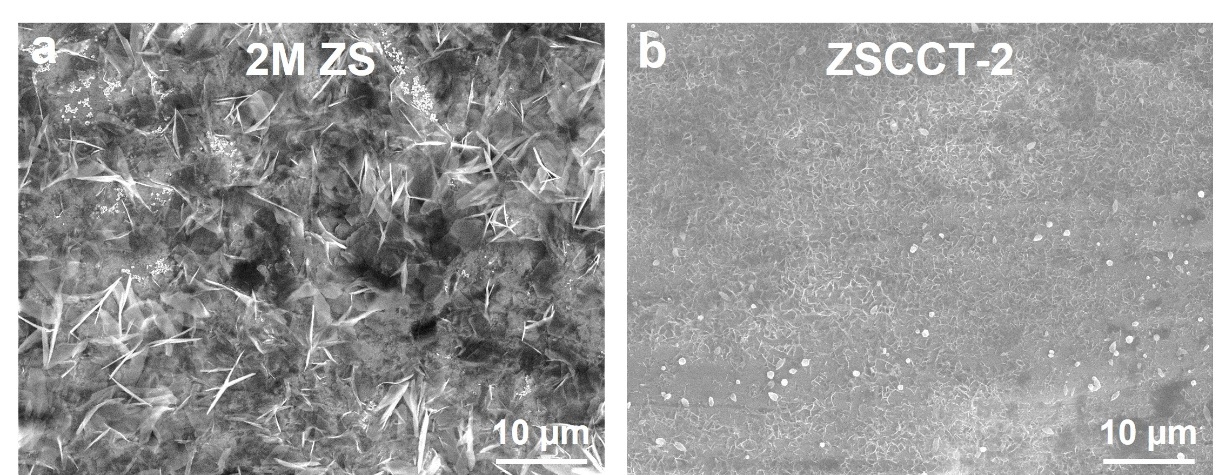
**

**Figure S29.** SEM images of the deposited Zn anodes in the various electrolytes.

**
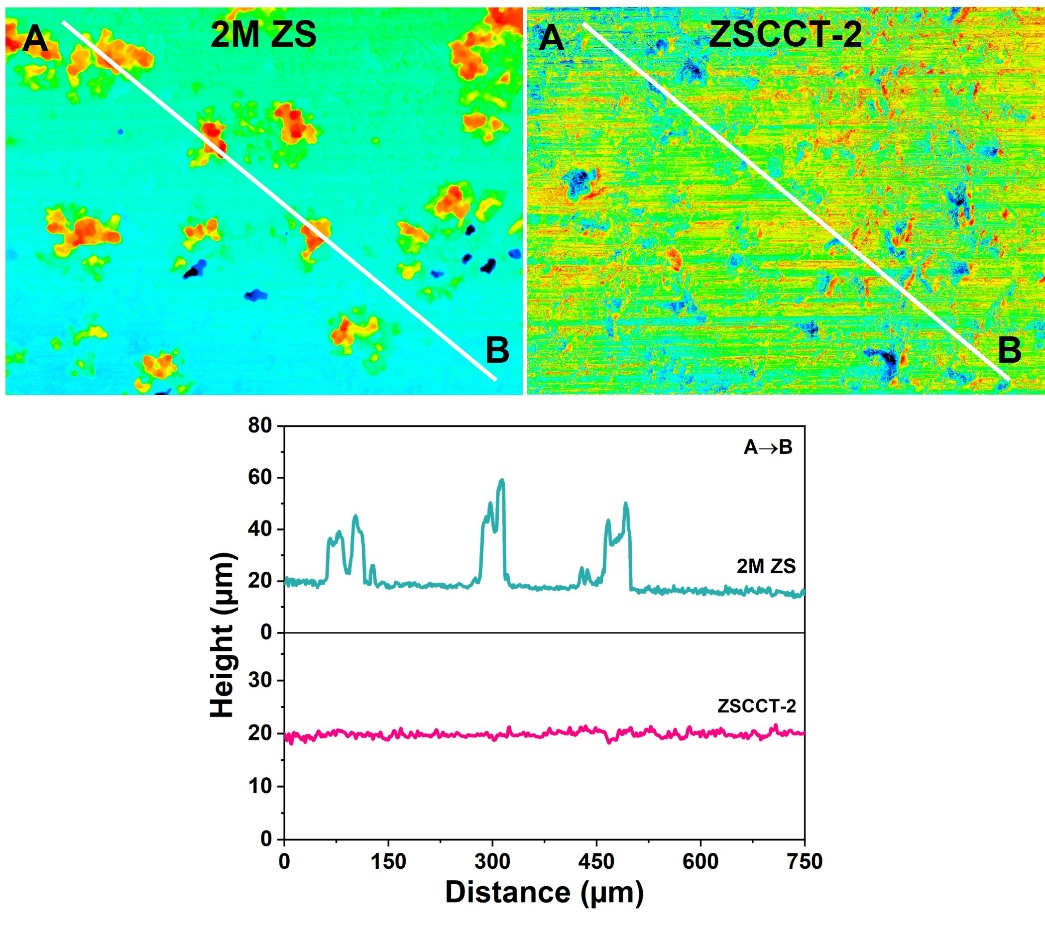
**

**Figure S30.** 2D LSCM images and the corresponding surface roughness curves of the deposited Zn surface after 20 cycles in the various electrolytes.

**
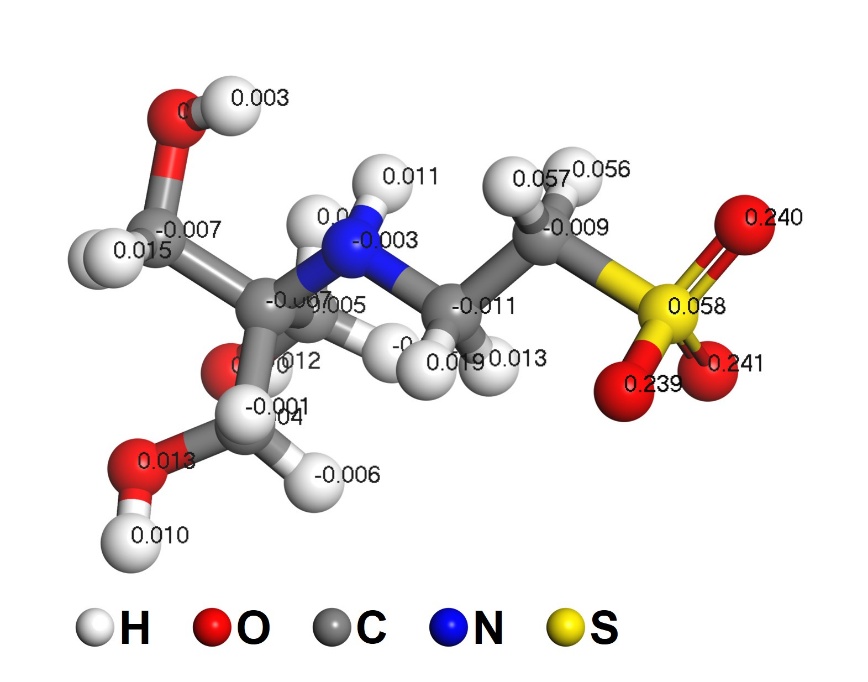
**

**Figure S31.** Fukui function (f^-^) of TES^-^.

**
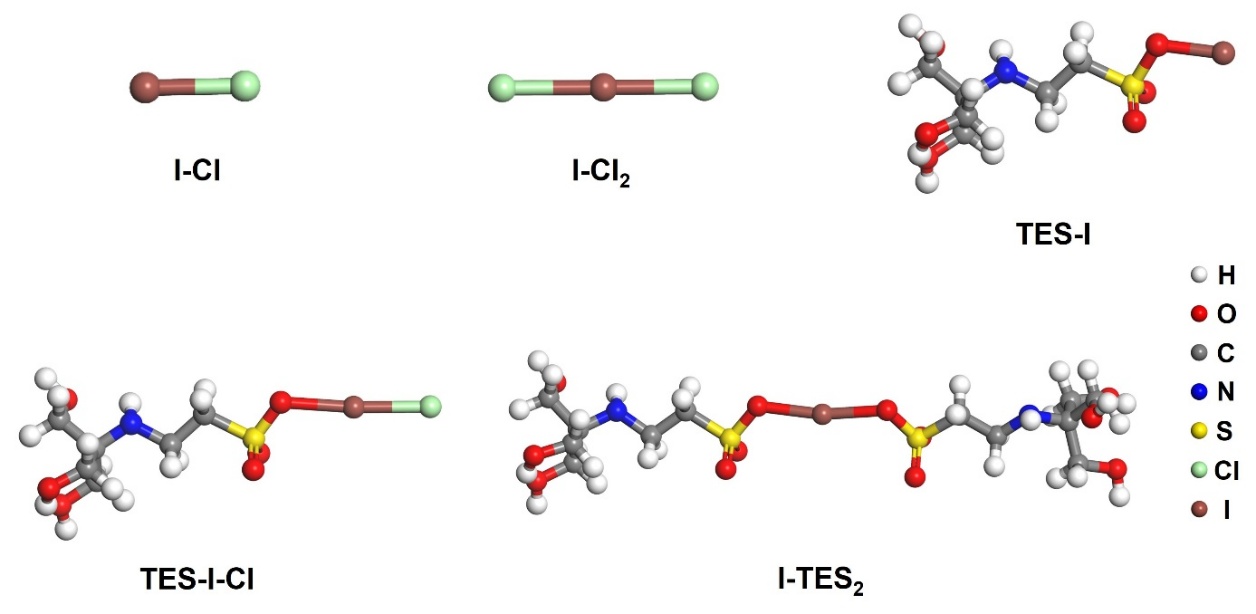
**

**Figure S32.** The representative coordination structures of I^+^ with different species.

**
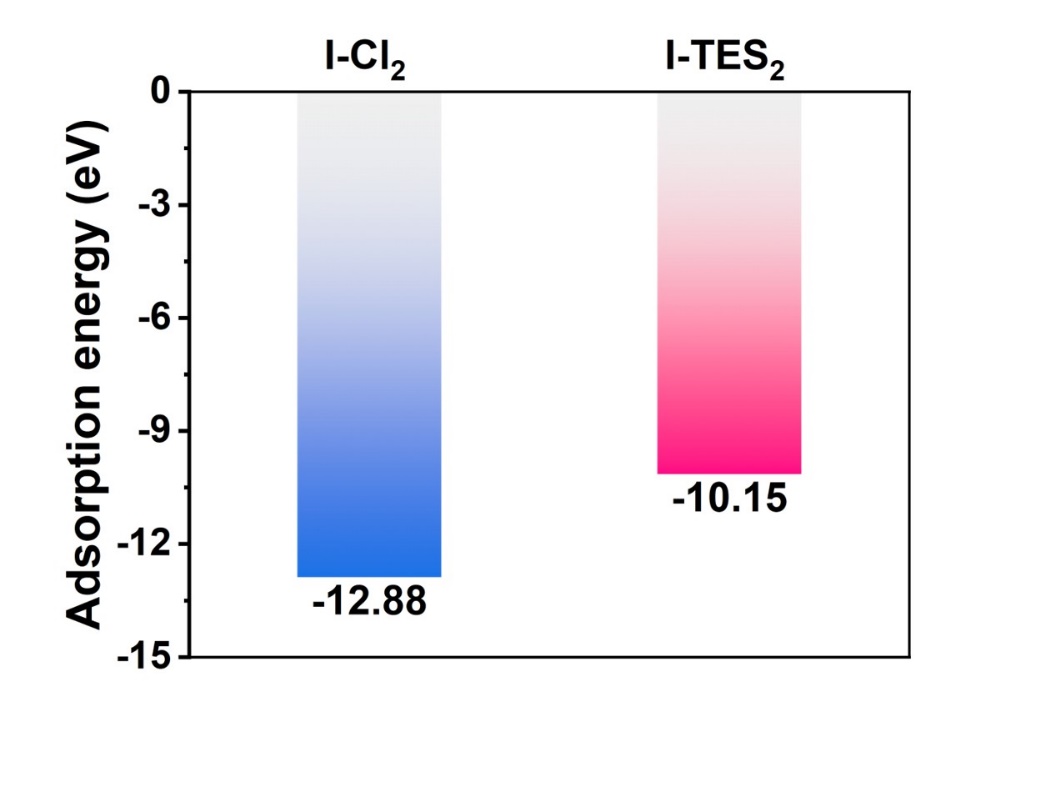
**

**Figure S33.** The adsorption energies of I^+^ coordination structures (I-Cl_2_ and I-TES_2_).


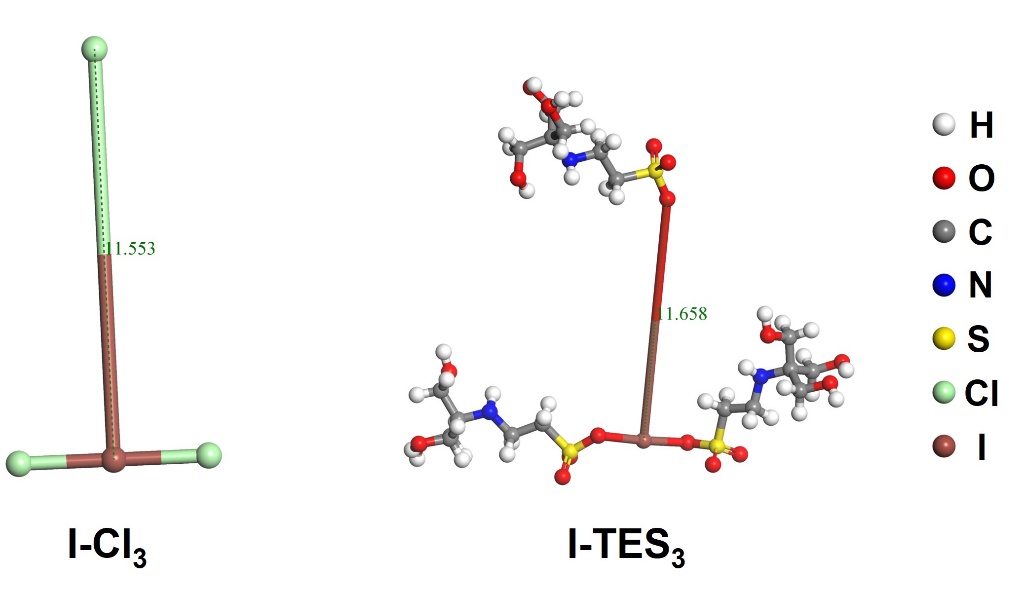


**Figure S34.** The representative three-coordination structures of I^+^ with Cl^-^ and TES^-^ ions.

**
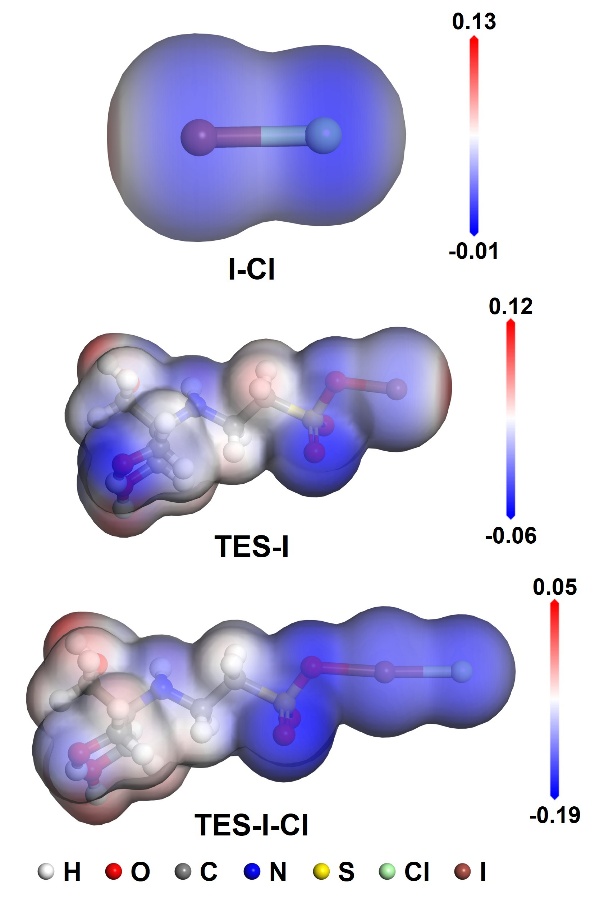
**

**Figure S35.** MESP mapping of various coordination structures (I-Cl, TES-I, and TES-I-Cl).


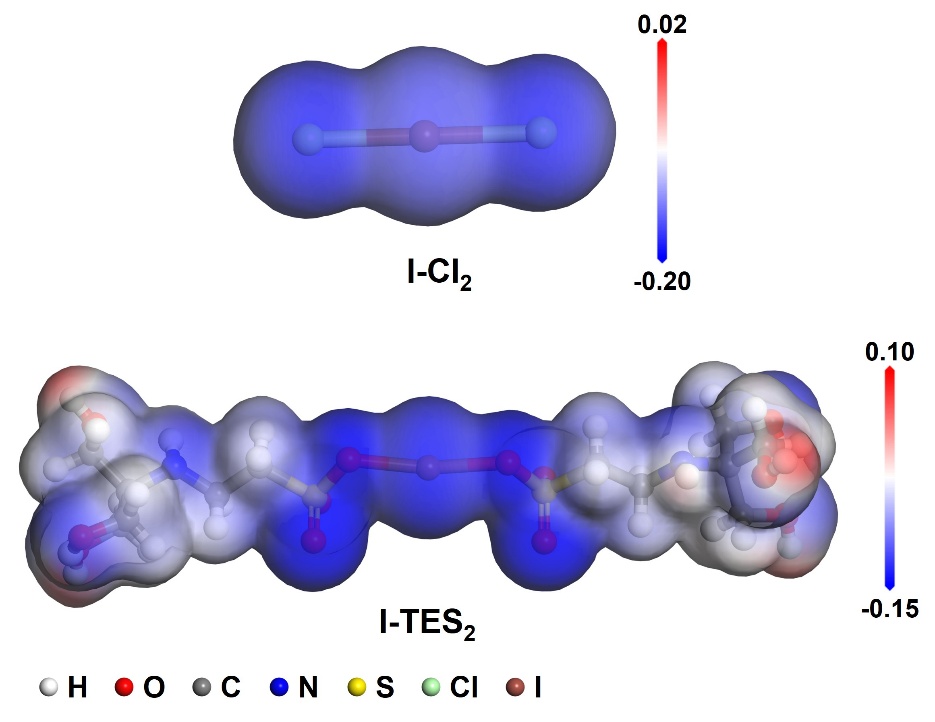


**Figure S36.** MESP mapping of various coordination structures (I-Cl_2_ and I-TES_2_).

**
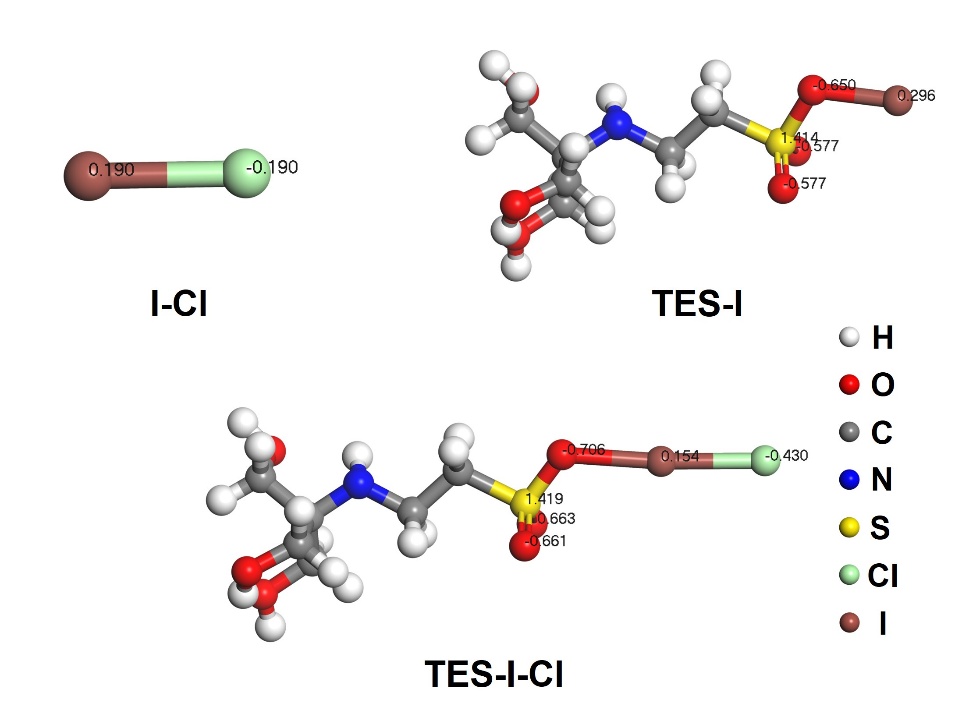
**

**Figure S37.** The detailed atomic charges of various coordination structures (I-Cl, TES-I, and TES-I-Cl).


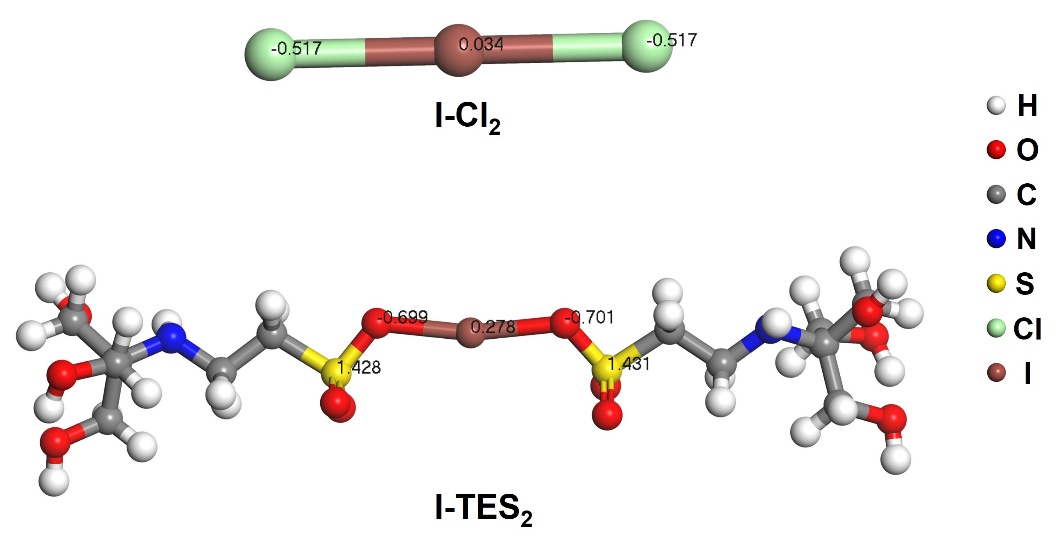


**Figure S38.** The detailed atomic charges of various coordination structures (I-Cl_2_ and I-TES_2_).

**
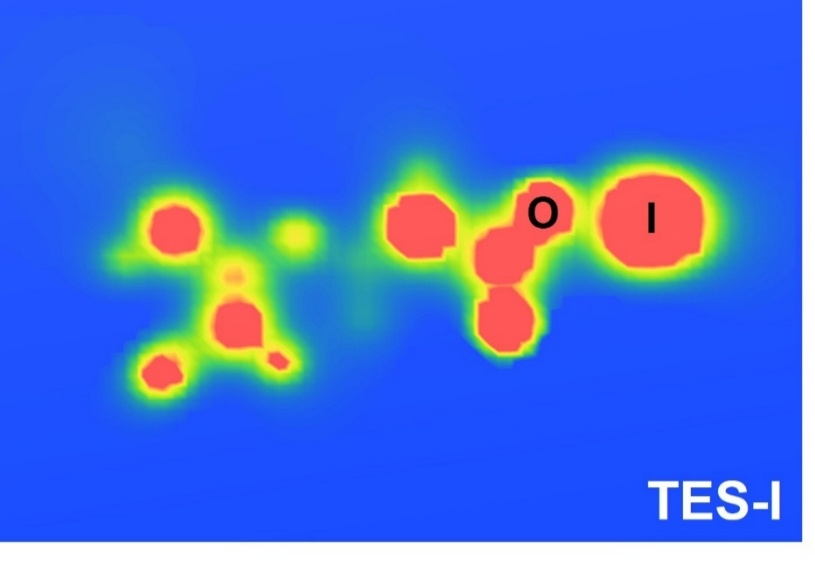
**

**Figure S39.** Electron localization function (ELF) of TES-I coordination structure.


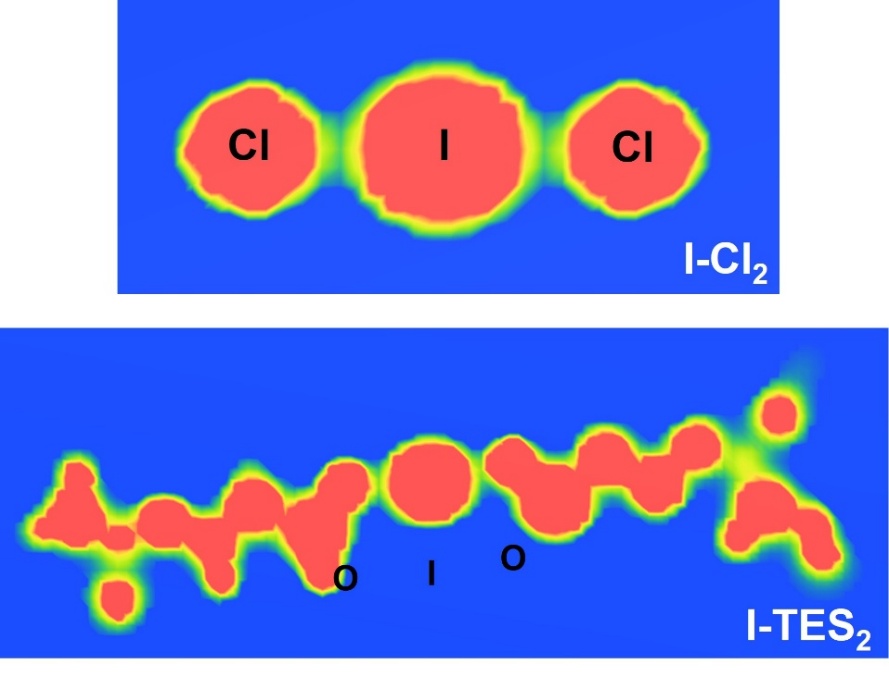


**Figure S40.** Electron localization function (ELF) of various coordination structures (I-Cl_2_ and I-TES_2_).


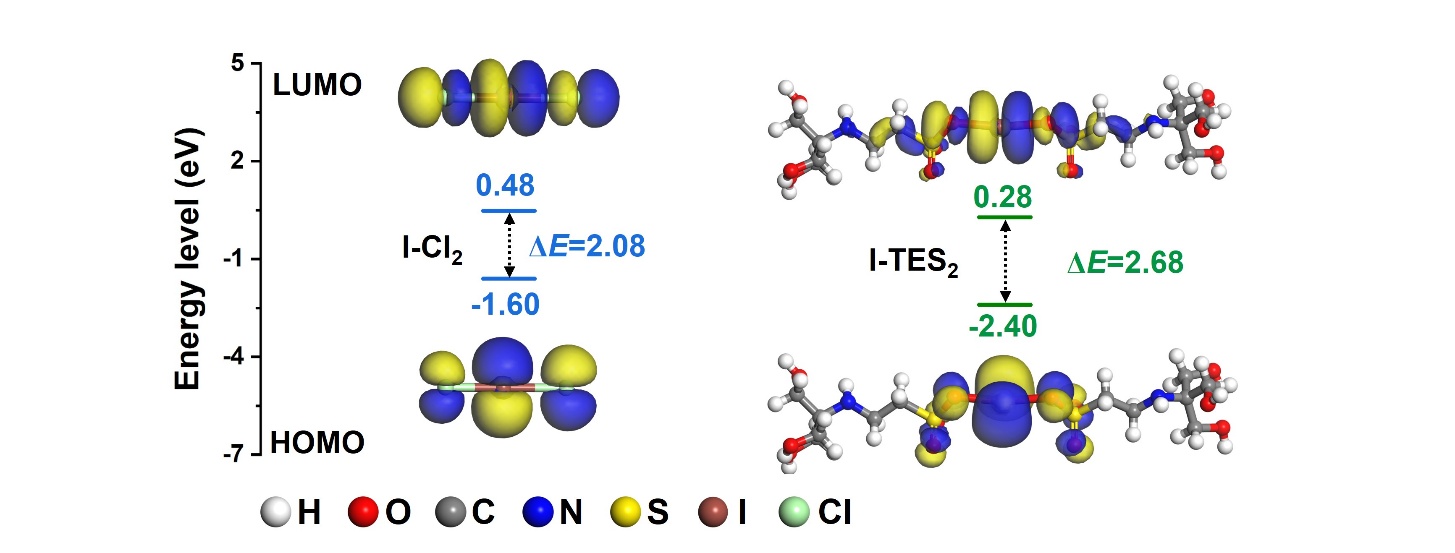


**Figure S41.** The frontier molecular orbital energy levels of various coordination structures (I-Cl_2_ and I-TES_2_).

**
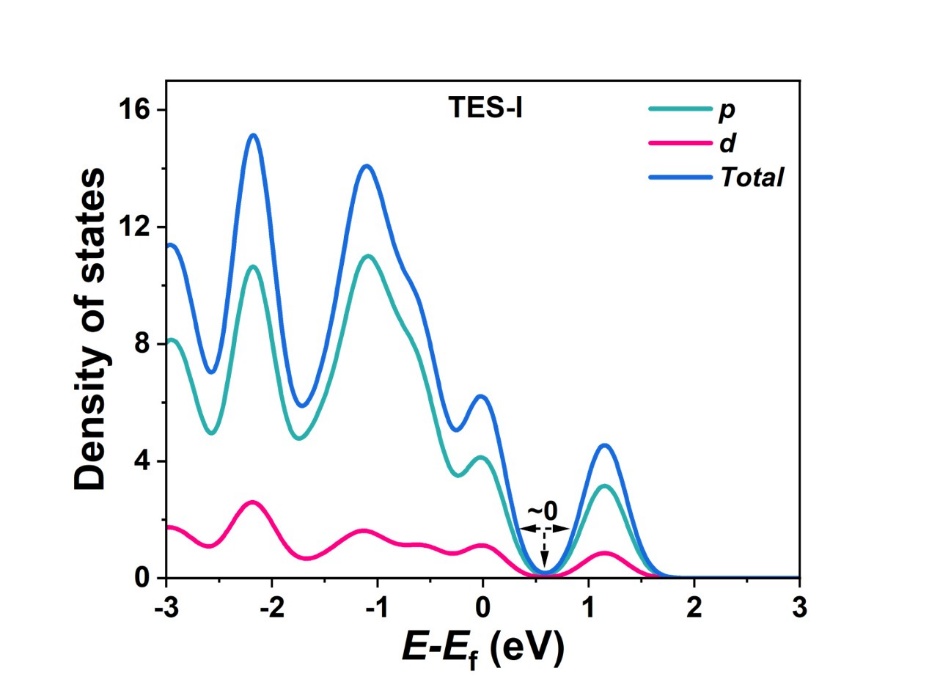
**

**Figure S42.** Density of states of TES-I coordination structure.


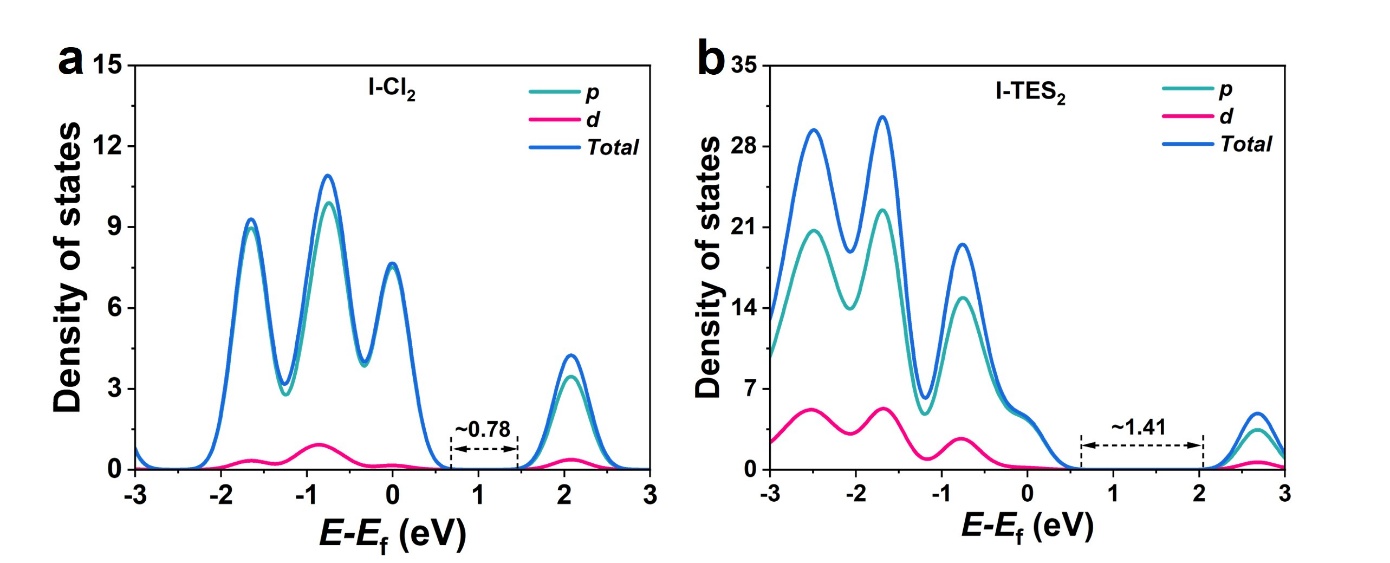


**Figure S43.** The density of states of various coordination structures (I-Cl_2_ and I-TES_2_).

**
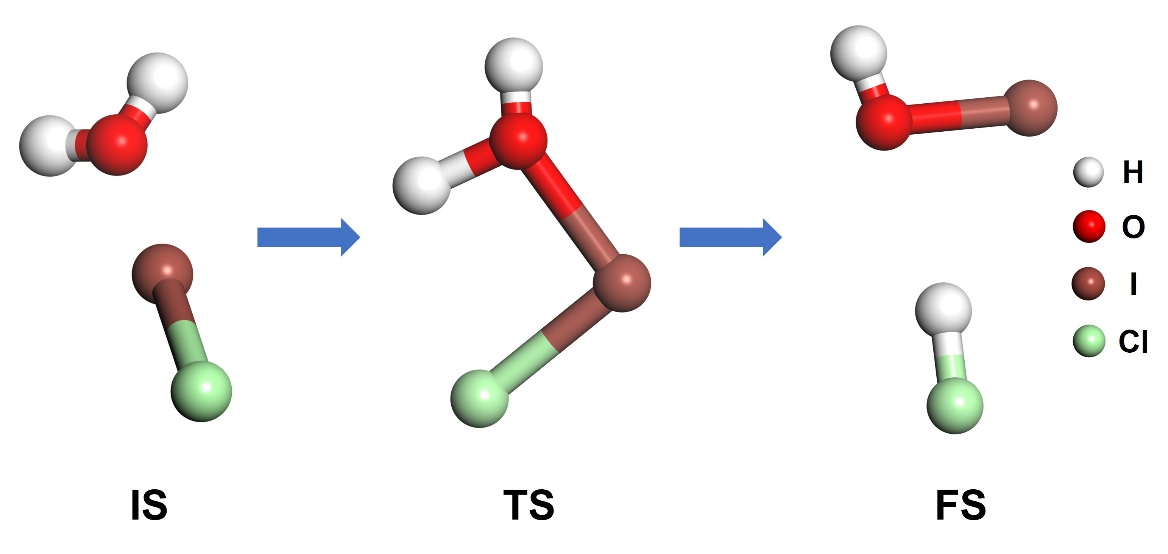
**

**Figure S44.** The initial, transition, and final structures (IS, TS, and FS) from hydrolysis reaction of ICl without TES^-^.

**
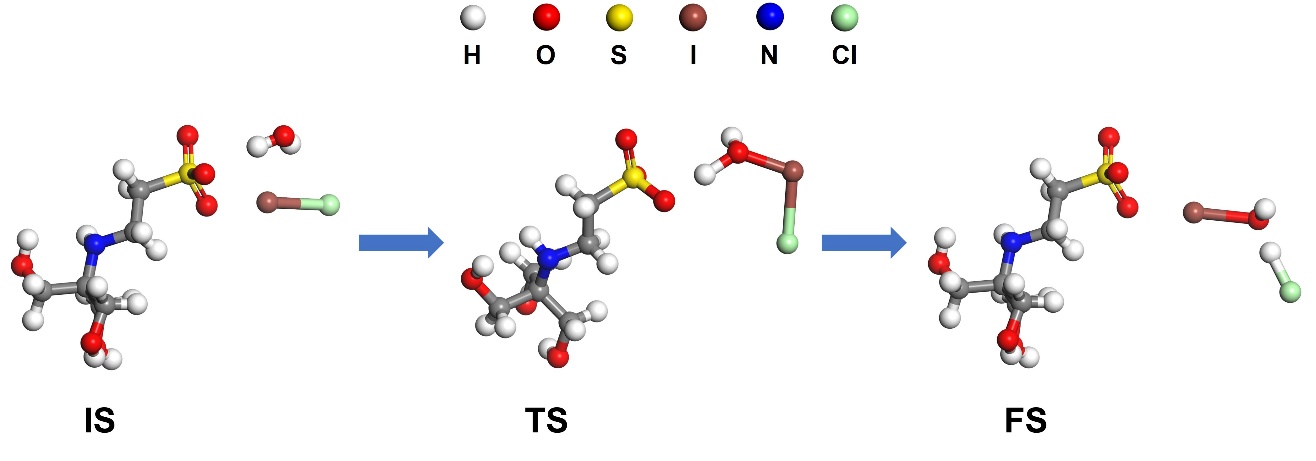
**

**Figure S45.** The initial, transition, and final structures (IS, TS, and FS) from hydrolysis reaction of ICl with TES^-^.

**
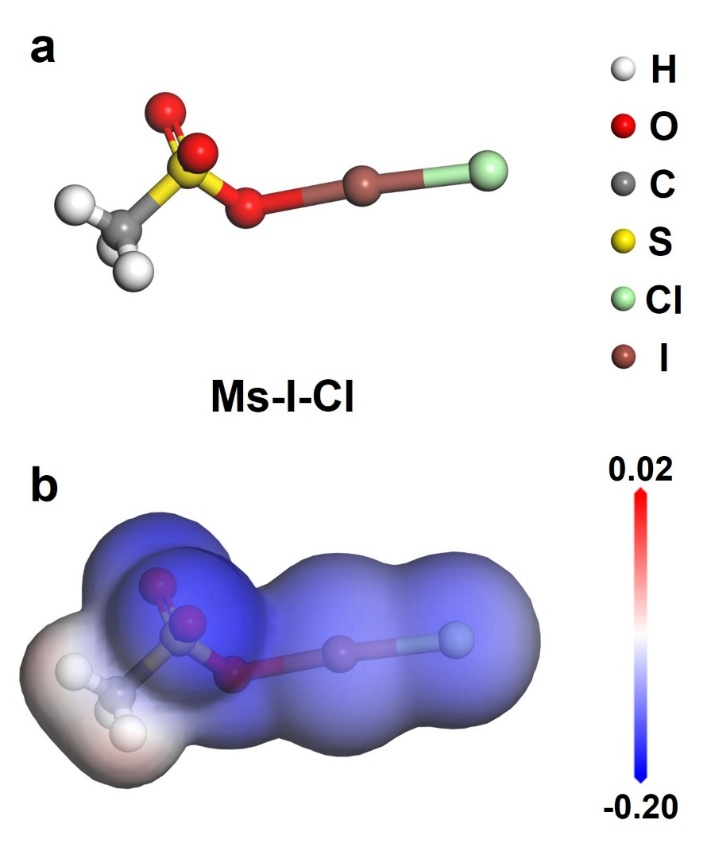
**

**Figure S46.** (a) The representative coordination structure of I^+^ with Ms^-^ and Cl^-^ ions. (b) MESP mapping of Ms-I-Cl coordination structure.

We simulated the representative I^+^ coordination structure with Ms^-^: Ms-I-Cl (Figure S46). The MESP mapping of Ms^-^ shows that the negative charge is similarly localized on the sulfonate oxygen atoms (Figure S47), confirming that Ms^-^ can coordinate with I^+^ in an analogous manner to TES^-^.


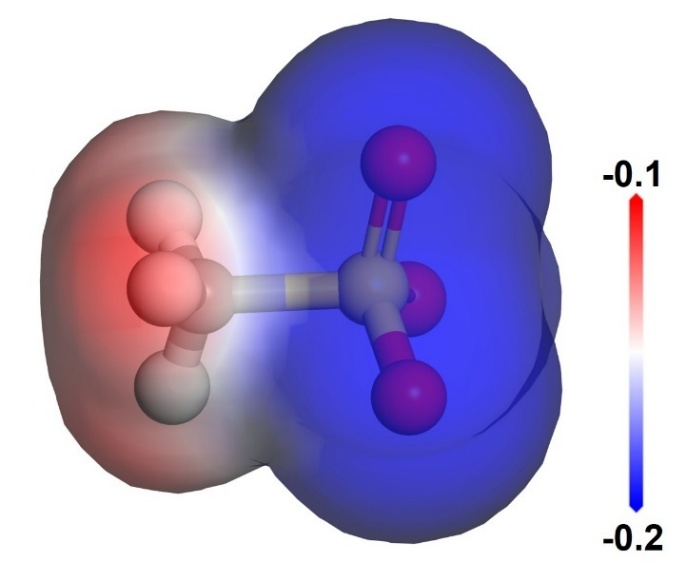


**Figure S47.** MESP mapping of Ms^-^.


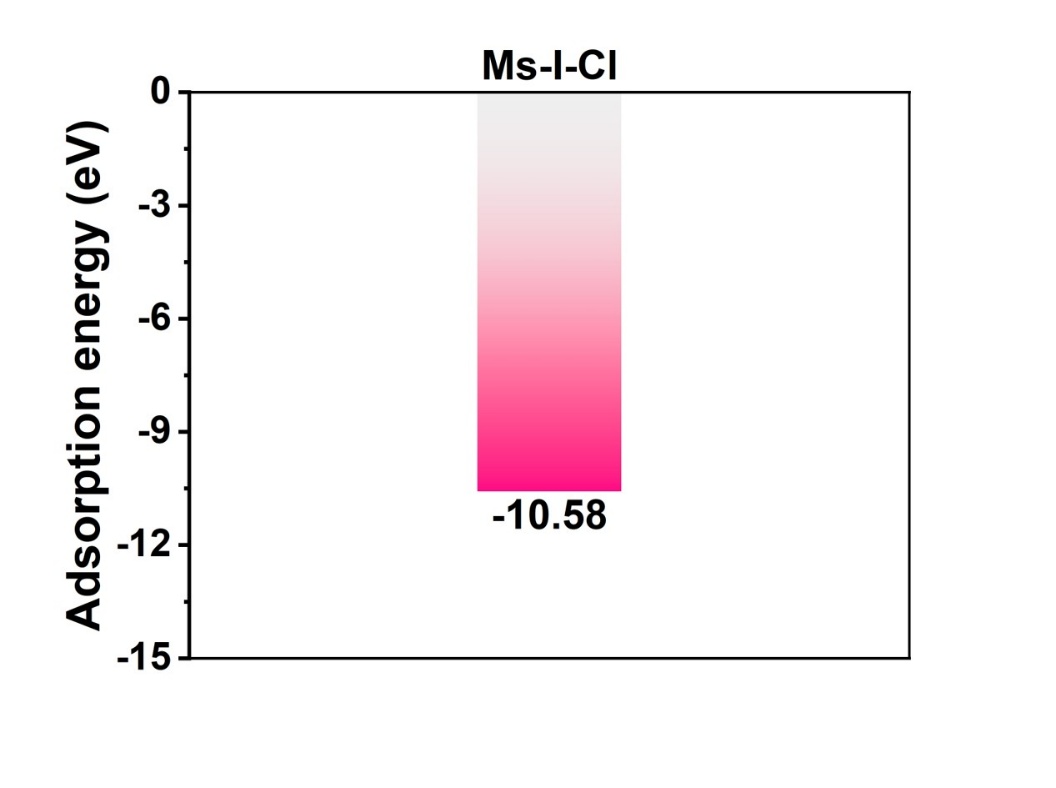


**Figure S48.** The adsorption energies of Ms-I-Cl coordination structure.

The calculated adsorption energy for the structure is -10.58 eV (Figure S48). This highly negative value confirms that the coordination structure is thermodynamically stable.


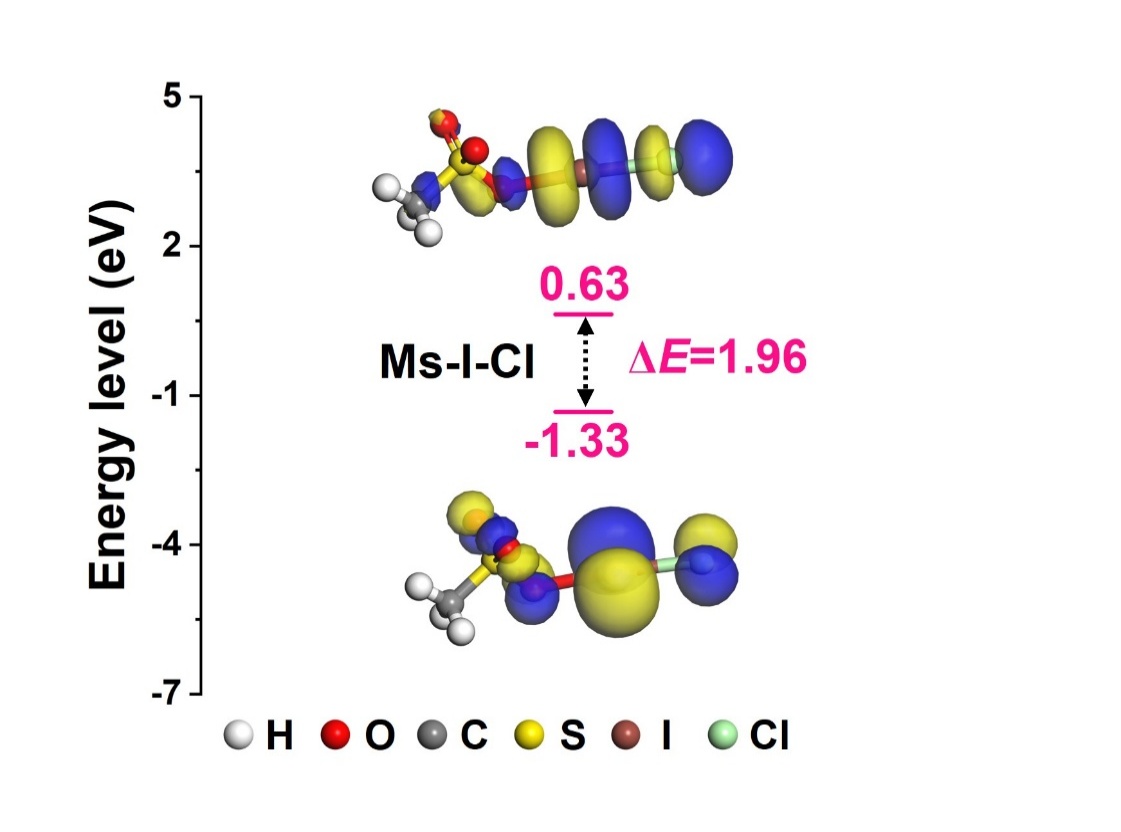


**Figure S49.** The frontier molecular orbital energy level of Ms-I-Cl coordination structure.

The HOMO-LUMO energy gap value for Ms-I-Cl is 1.96 eV (Figure S49). Furthermore, the calculated band gap for Ms-I-Cl is 0.92 eV (Figure S50). These electronic structure parameters indicate that while Ms^-^ can coordinate with I^+^ and form stable complexes, its small molecular size provides only a limited steric shielding effect. In comparison, as reported in our manuscript (Figure 4e, g), the TES-I-Cl coordination structure exhibits much wider HOMO-LUMO gap and larger band gap, which correlate with higher resistance to hydrolysis of I^+^.


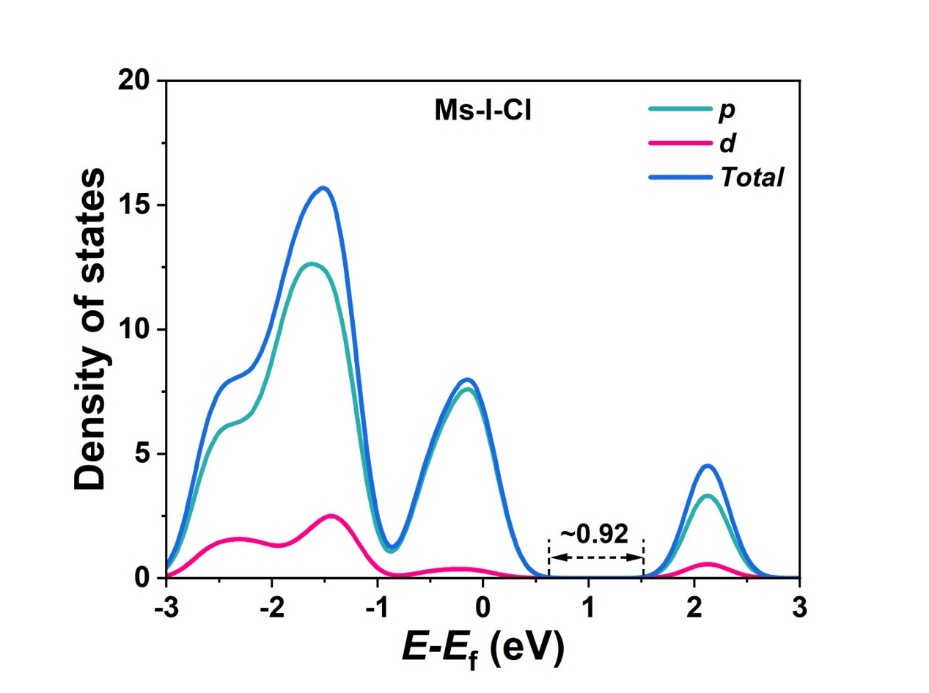


**Figure S50.** The density of states of Ms-I-Cl coordination structure.


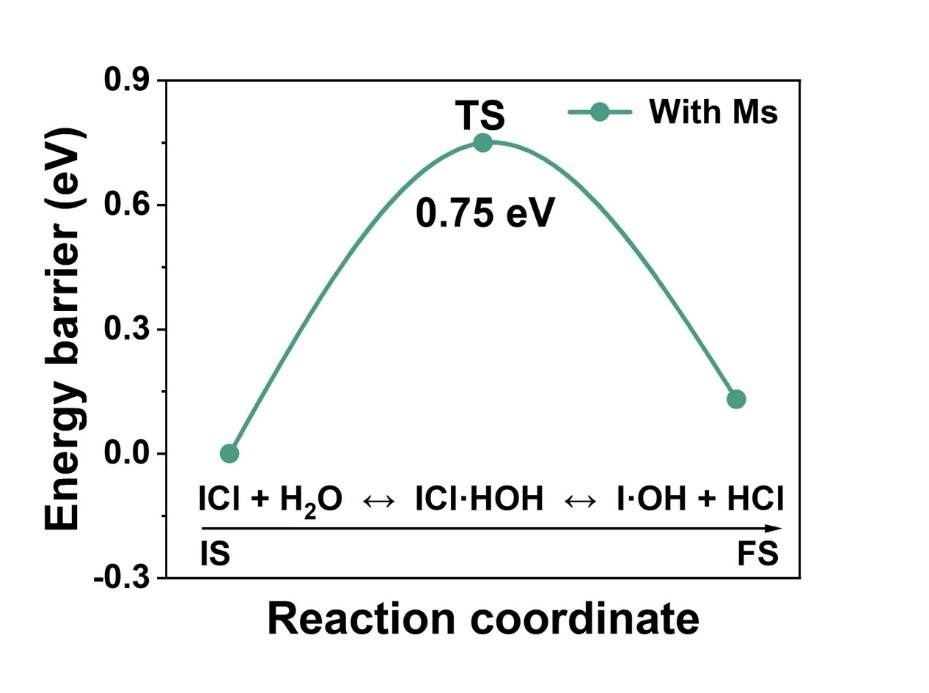


**Figure S51.** The energy barrier of ICl hydrolysis reaction with Ms^-^.

The calculated energy barrier for the hydrolysis reaction of the I^+^-Ms complex is 0.75 eV (Figures S51 and S52), which is only slightly higher than that of the I^+^-Cl complex (0.31 eV) but substantially lower than that of the I^+^-TES complex (1.26 eV). This clearly demonstrates that the bulky TES^-^ ligand, rather than the sulfonate coordination itself, is responsible for the dramatically increased hydrolysis energy barrier through its physical shielding effect.


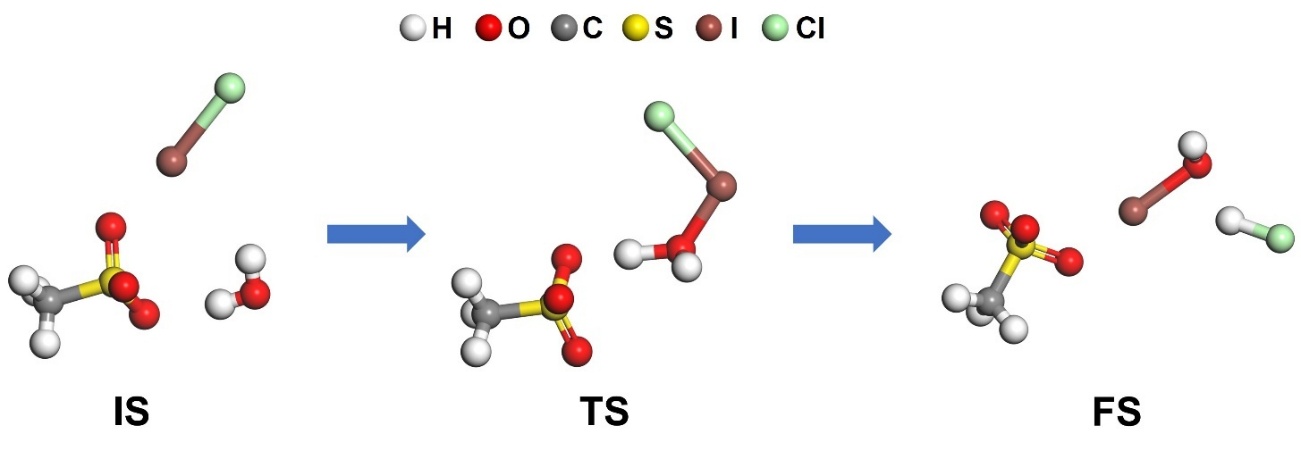


**Figure S52.** The initial, transition, and final structures (IS, TS, and FS) from hydrolysis reaction of ICl with Ms^-^.


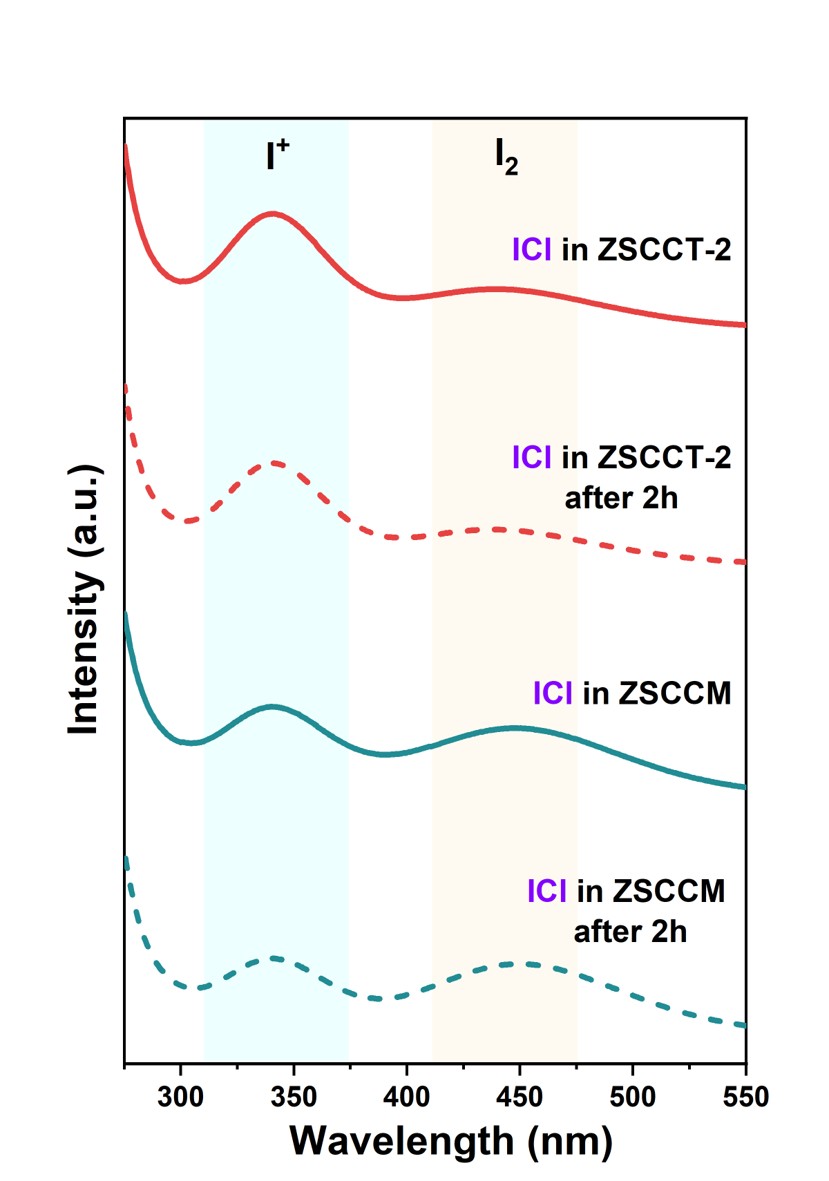


**Figure S53.** UV-vis spectra of ICl in the different electrolytes before and after 2h.

We prepared a control electrolyte containing 2M ZnSO_4_, 5M ChCl, and 0.5M Ms-Na (labeled as ZSCCM), and compared it with our optimized ZSCCT-2 electrolyte containing TES-Na. In the ZSCCM electrolyte, UV-vis spectroscopy reveals the presence of the characteristic I^+^ absorption band at ~345 nm. However, a distinct I_2_ peak is also observed, suggesting that partial hydrolysis of I^+^ still occurs (Figure S53). After 2 hours, the intensity of the I_2_ peak increases, while the I^+^ peak diminishes, confirming that hydrolysis becomes more pronounced over time. This behavior contrasts sharply with that observed in the ZSCCT-2 electrolyte containing TES^-^, where the I^+^ peak remains stable for over two hours with negligible I_2_ formation (Figure S53). These results further support that the steric-hindrance effect provided by the bulky TES^-^ ligand, rather than simple sulfonate coordination from Ms^-^, is essential for effectively suppressing I^+^ hydrolysis.

**
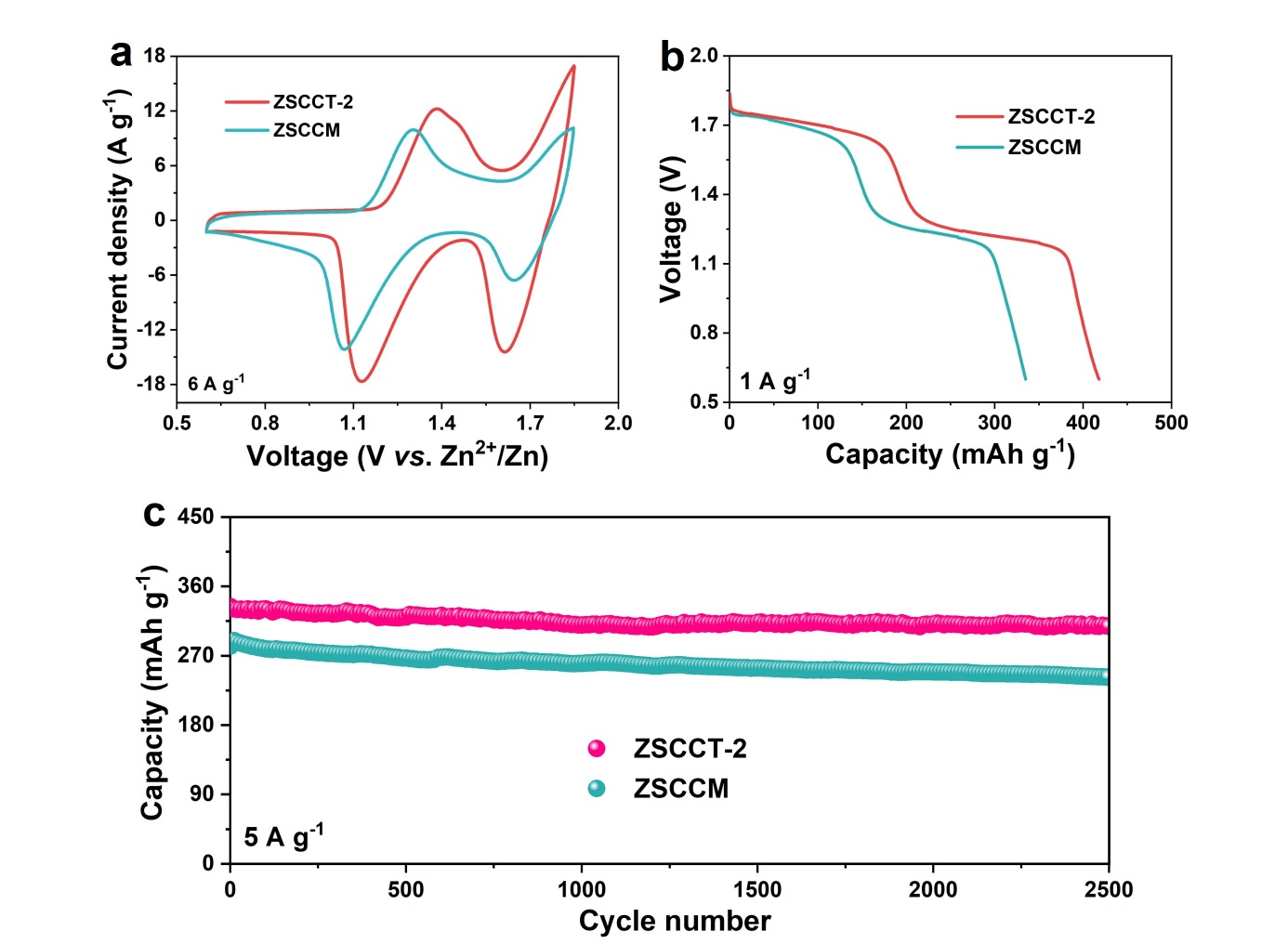
**

**Figure S54.** (a) CV curves and (b) discharge curves of zinc-iodine batteries in the various electrolytes. (c) Cycling stability of zinc-iodine batteries at 5 A g^-1^ over 2,500 cycles in the various electrolytes.

To directly compare the effectiveness of TES^-^ and Ms^-^ in suppressing I^+^ hydrolysis and enabling stable four-electron conversion, we evaluated the electrochemical performance of zinc-iodine batteries using the ZSCCT-2 and ZSCCM electrolytes. As shown in Figure S54a, CV curves of the zinc-iodine batteries with these electrolytes exhibit two pairs of oxidation and reduction peaks, which can be assigned to the reversible reactions of I^0^/I^-^ at low voltage and I^+^/I^0^ at high voltage, respectively. Specifically, the battery with the ZSCCT-2 electrolyte displays a relatively larger response current than the case of ZSCCM, indicating the enhanced reactivity and redox kinetics of I^+^/I^0^/I^-^ conversion. More importantly, the discharge voltage plateau for the I^+^/I^0^ conversion is more clearly defined and stable in the ZSCCT-2 electrolyte (Figure S54b), indicating a more reversible four-electron conversion process. In contrast, the zinc-iodine battery with ZSCCM electrolyte shows a less distinct high-voltage plateau and a lower overall capacity, consistent with partial I^+^ hydrolysis. Figure S54c presents the cycling performance of the zinc-iodine batteries with the ZSCCT-2 and ZSCCM electrolytes. The battery with the ZSCCT-2 electrolyte maintains a high capacity of ~333 mAh g^-1^ with excellent stability, showing only a slight decline to 310 mAh g^-1^ after 2,500 cycles. In contrast, the battery with the ZSCCM electrolyte displays a lower initial capacity of ~283 mAh g^-1^ and gradually decays to about 242 mAh g^-1^ after 2,500 cycles. The lower initial capacity and continuous capacity decay in the ZSCCM electrolyte are evidence of progressive I^+^ hydrolysis during cycling due to the insufficient steric-hindrance effect provided by the small Ms^-^ anion.


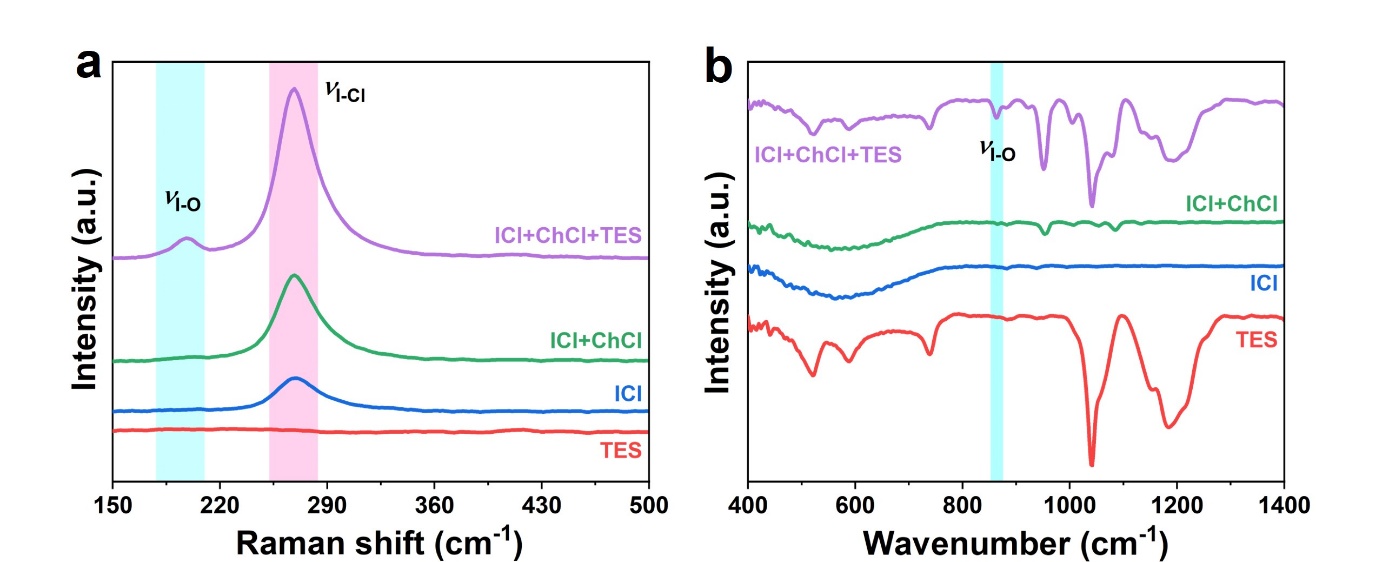


**Figure S55.** (a) Raman and (b) FTIR spectra of the various solutions.

**
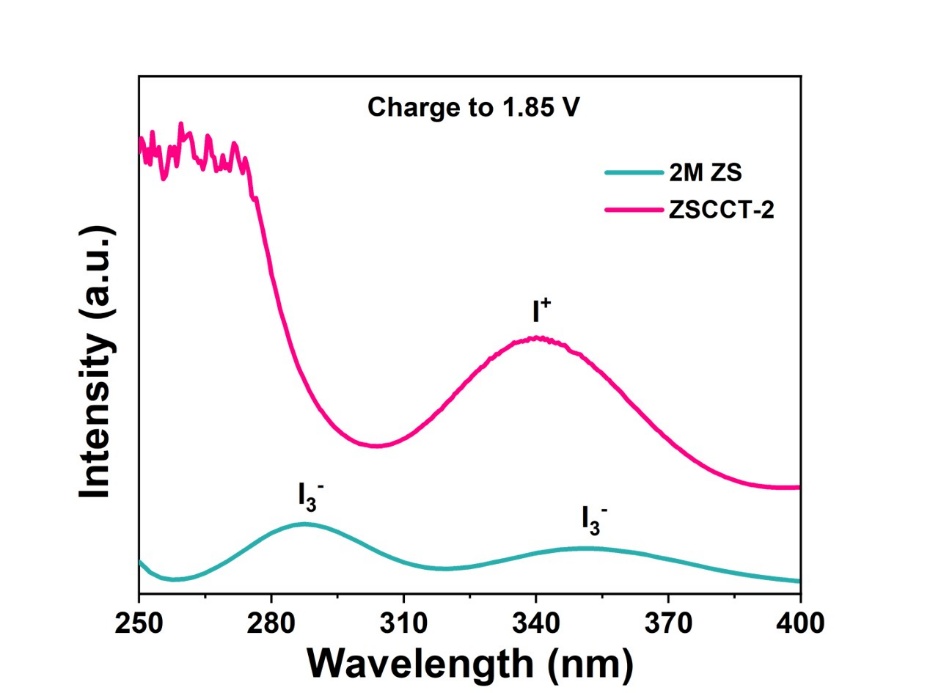
**

**Figure S56.** UV-vis spectra of the fully charged cathodes in the various electrolytes.

**
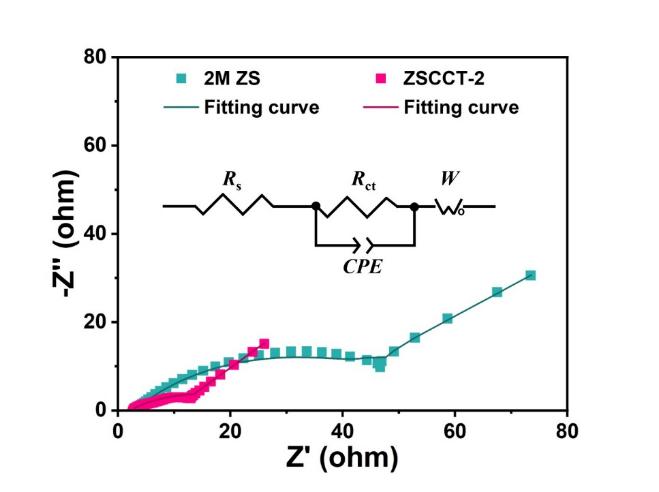
**

**Figure S57.** EIS and fitting curves of zinc-iodine batteries with 2M ZS and ZSCCT-2 electrolytes and the corresponding equivalent circuit model.

**
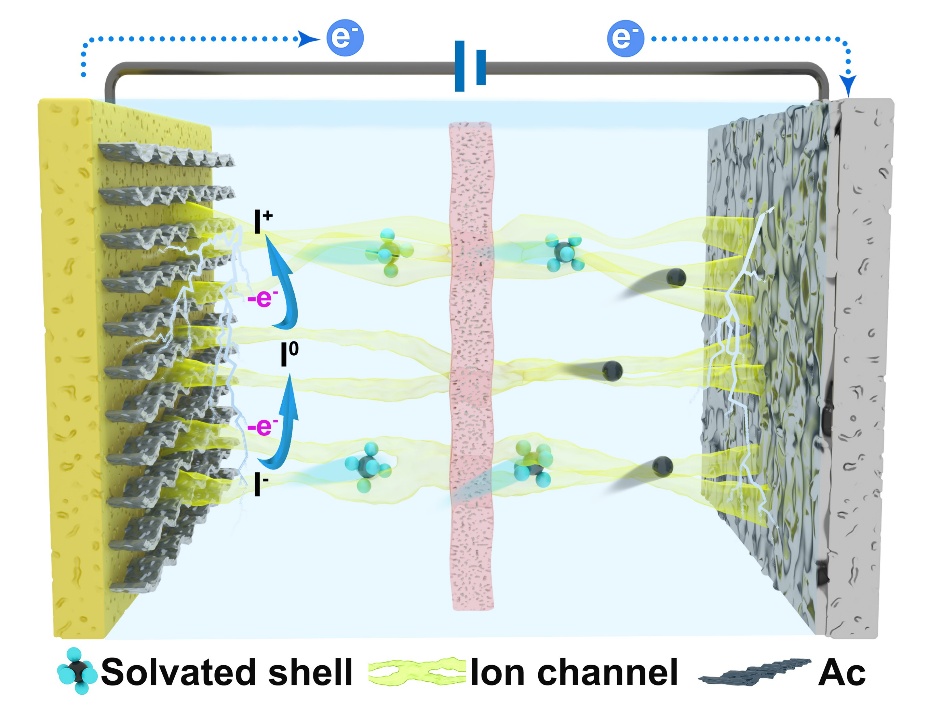
**

**Figure S58.** Schematic illustration of zinc-iodine full battery with highly reversible I^+^/I^0^/I^-^ conversion reaction.


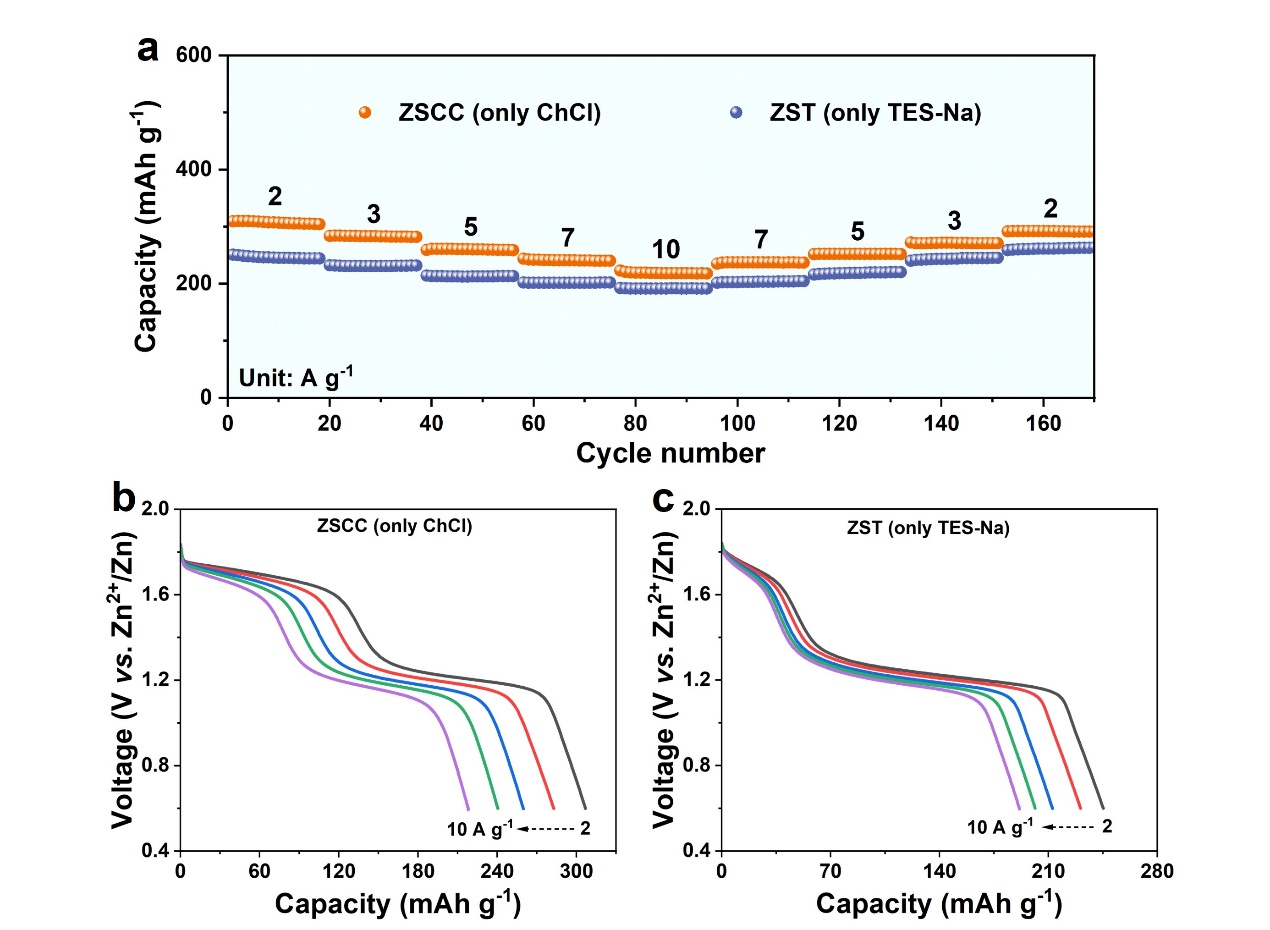


**Figure S59.** (a) Rate capability of the zinc-iodine batteries with ZSCC (with only ChCl) and ZST (with only TES-Na) electrolytes, and (b, c) the corresponding discharge curves at different current densities.


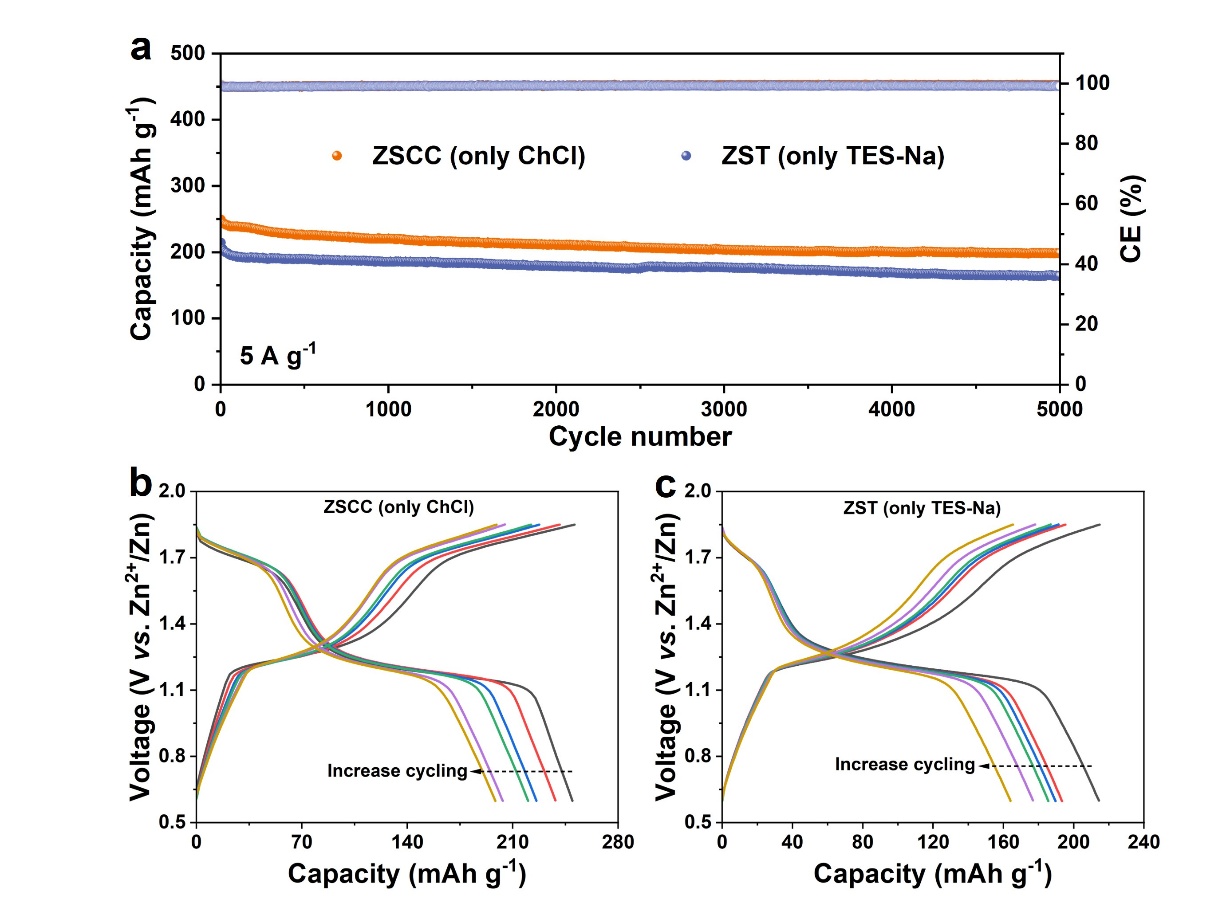


**Figure S60.** (a) Cycling stability of the zinc-iodine batteries with ZSCC (with only ChCl) and ZST (with only TES-Na) electrolytes, and (b, c) the corresponding discharge/charge curves.

**
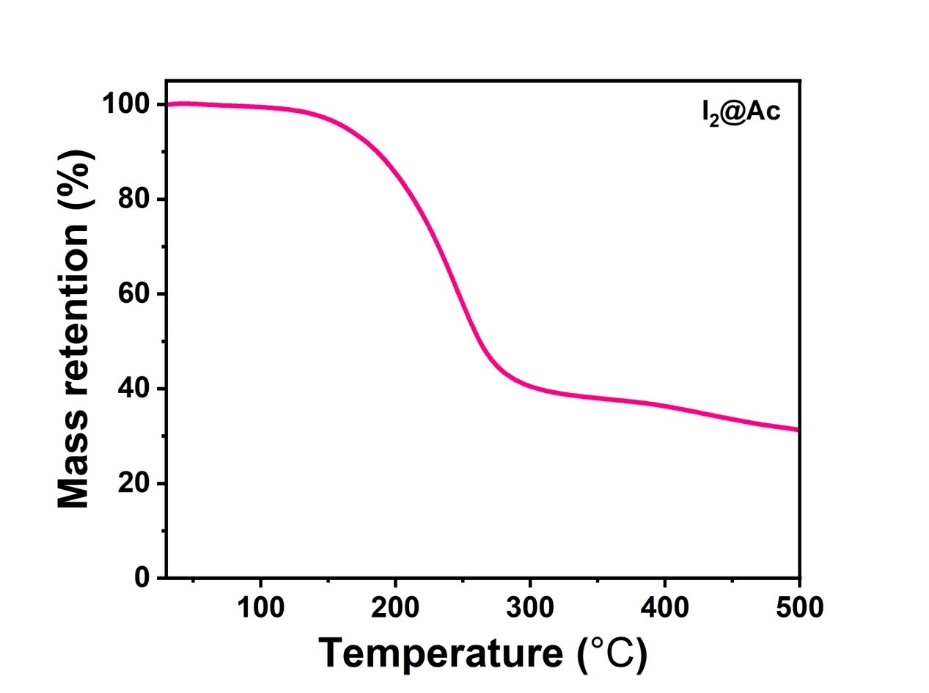
**

**Figure S61.** TG analysis of I_2_@Ac at 5 °C min^-1^.

**
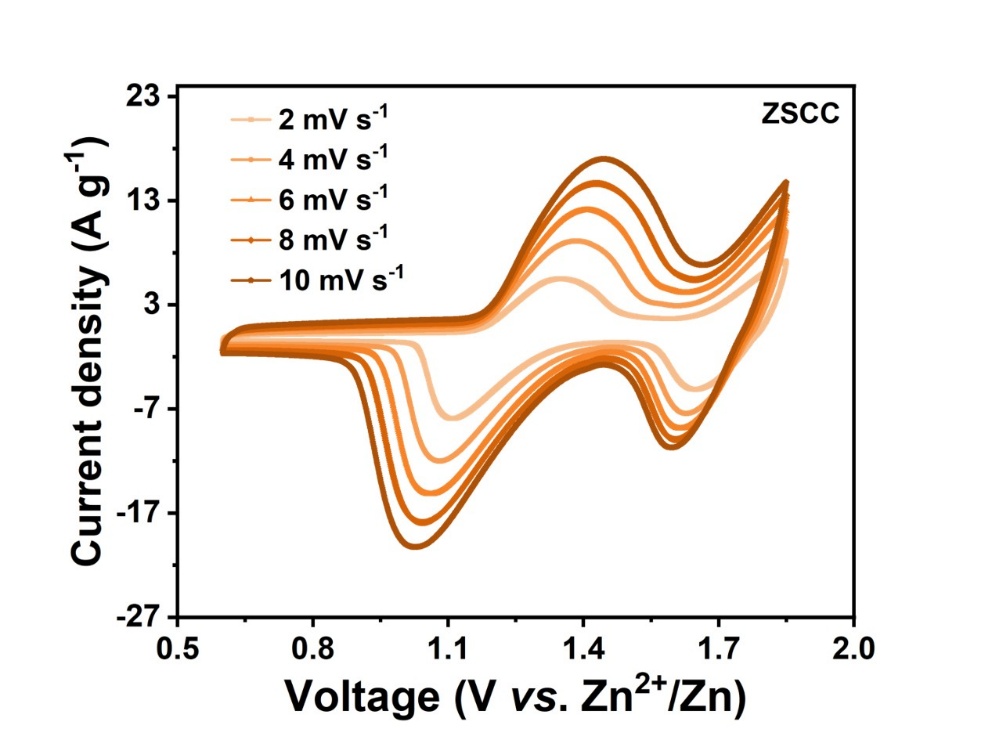
**

**Figure S62.** CV curves of zinc-iodine battery at various scan rates with the ZSCC electrolyte.

**
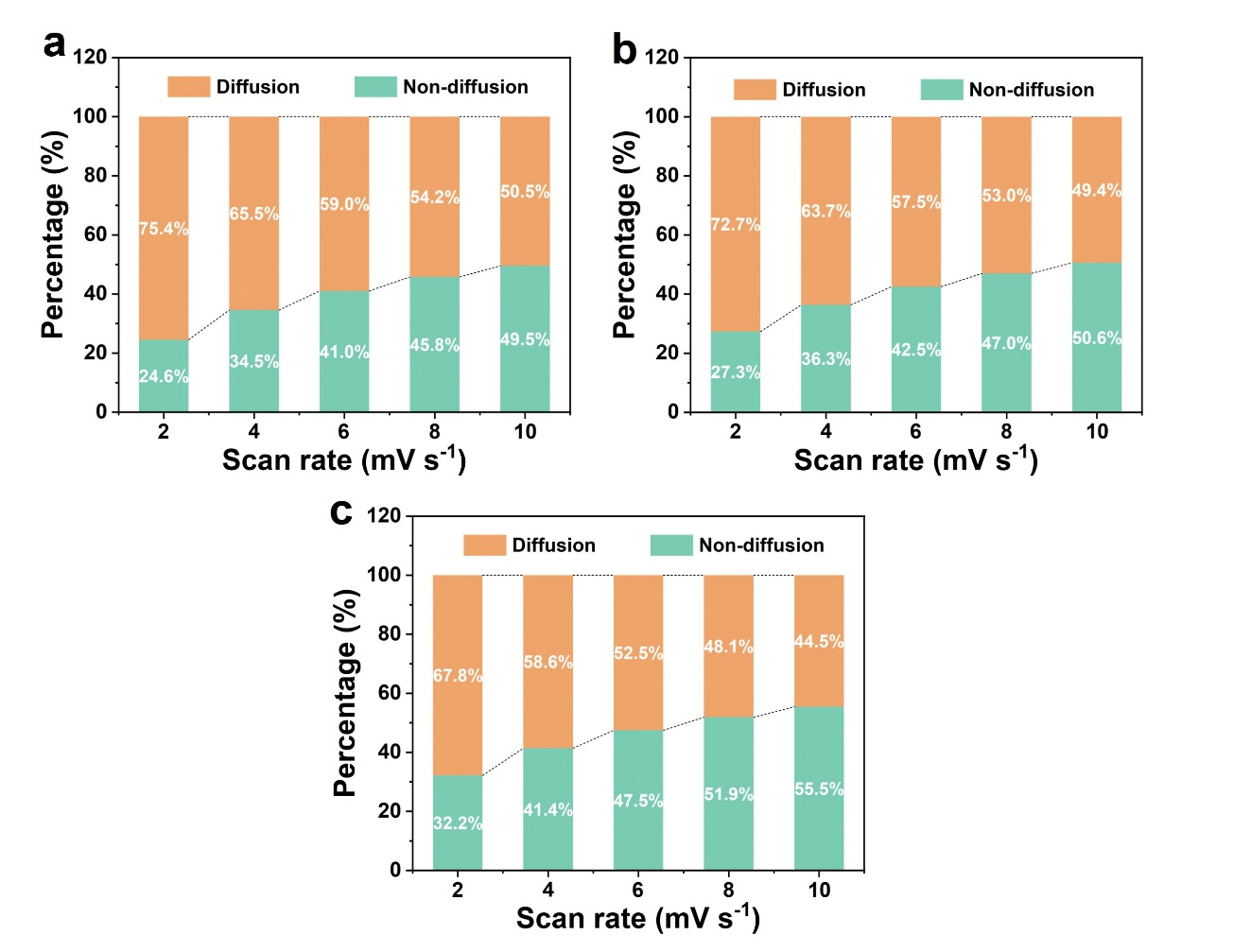
**

**Figure S63.** The capacitive contributions of zinc-iodine batteries at different scan rates in the various electrolytes. (a) 2M ZS, (b) ZSCC, and (c) ZSCCT-2.

**
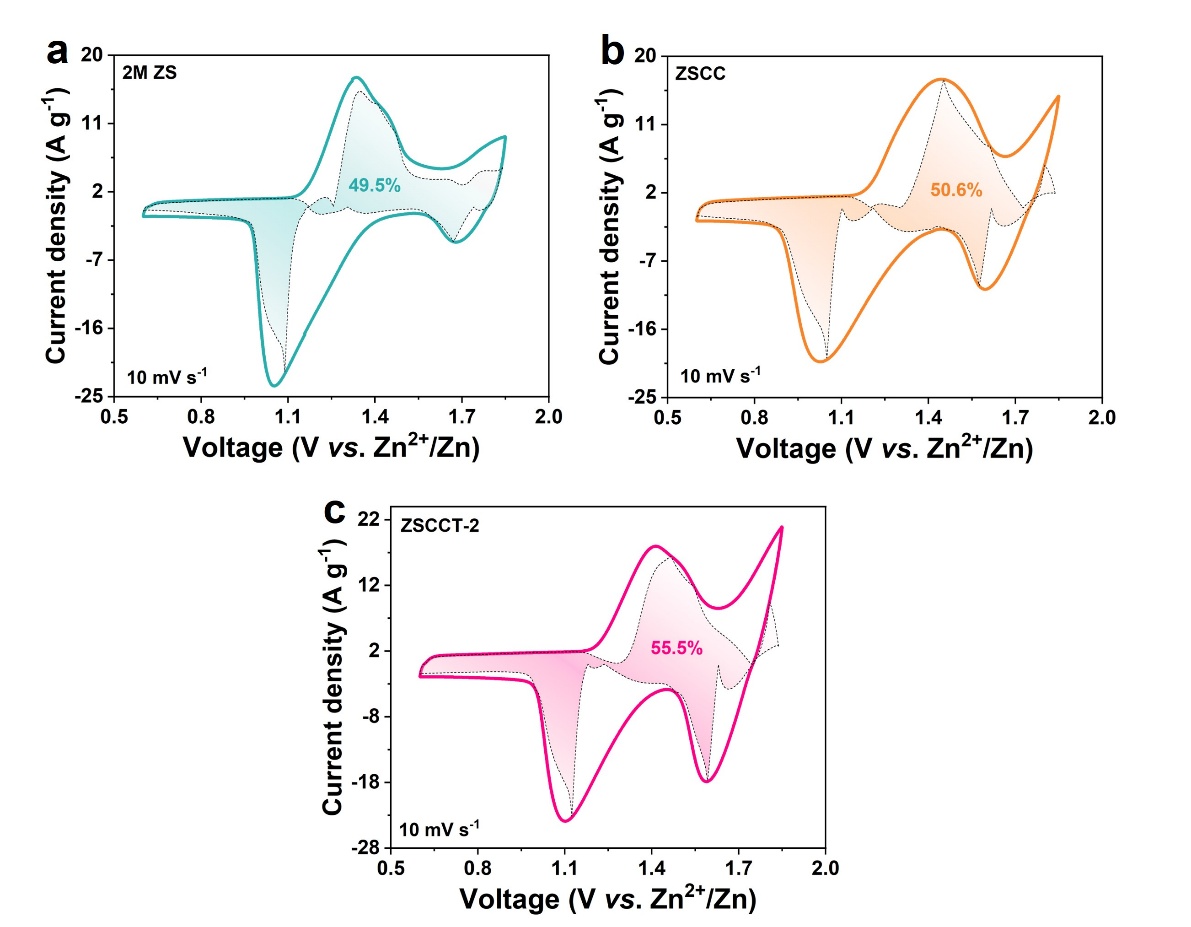
**

**Figure S64.** The CV profiles with the capacitive contribution of zinc-iodine batteries at 10 mV s^-1^ in the various electrolytes. (a) 2M ZS, (b) ZSCC, and (c) ZSCCT-2.

**
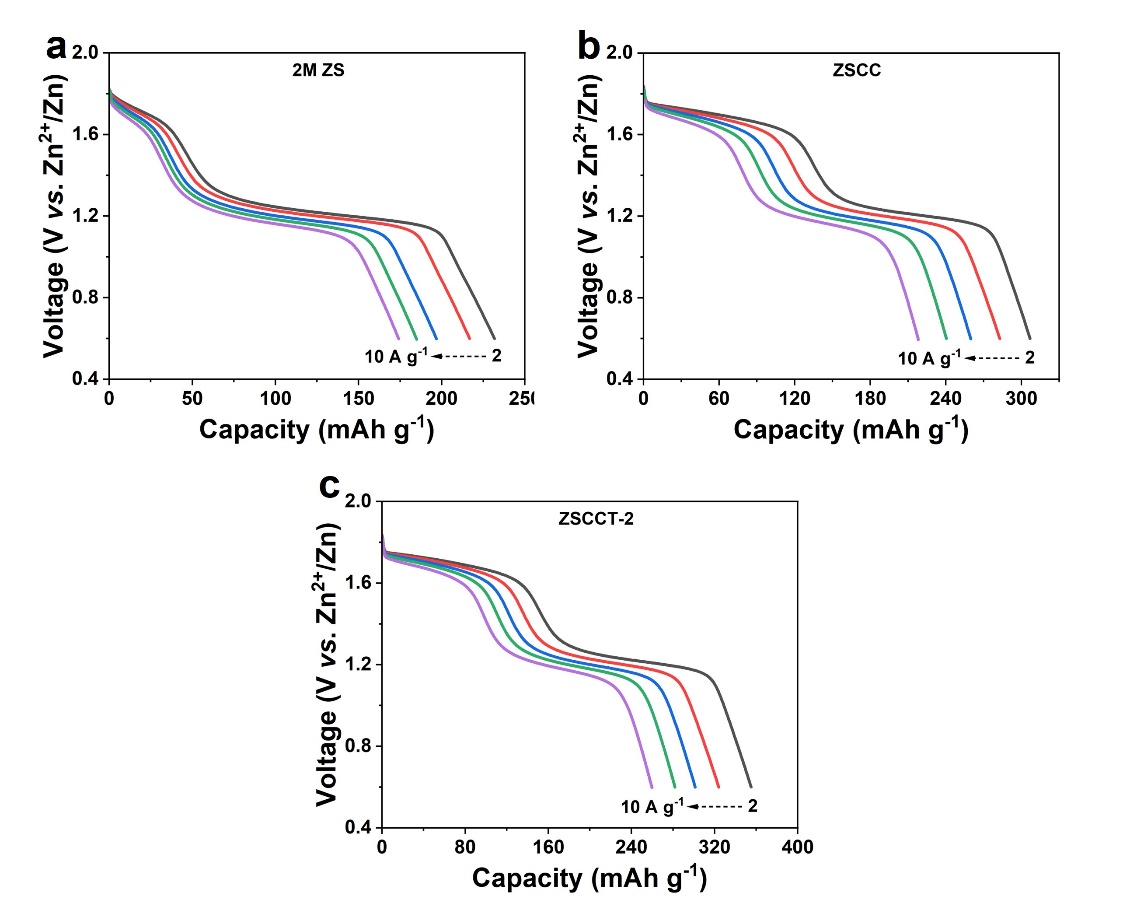
**

**Figure S65.** Discharge curves of zinc-iodine batteries at different current densities in the various electrolytes.


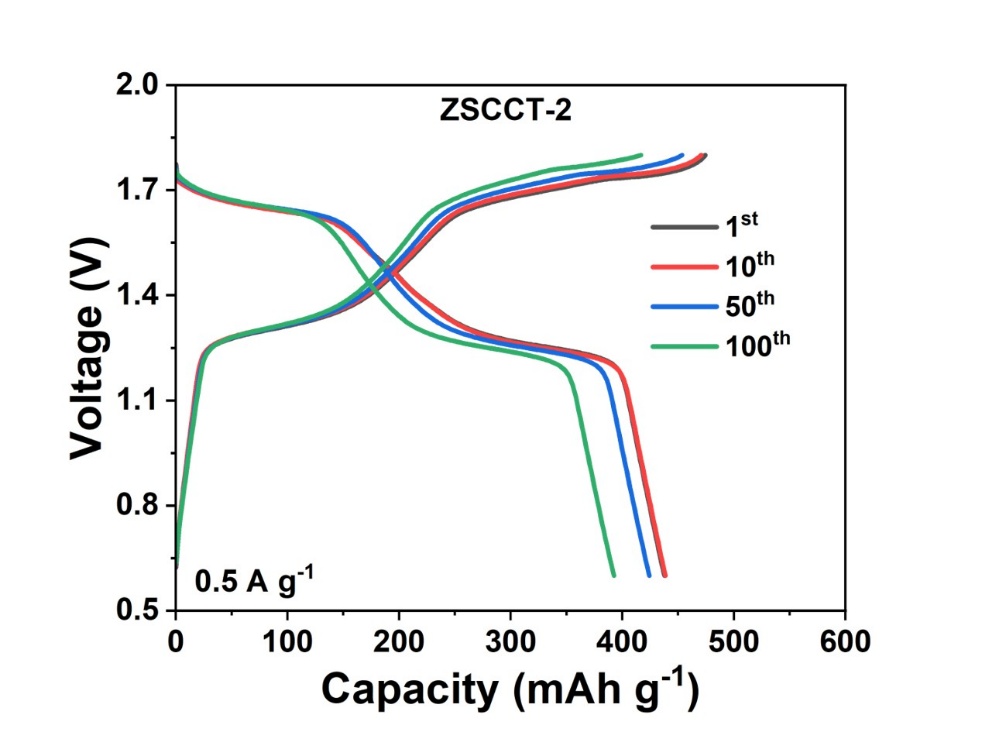


**Figure S66.** Galvanostatic discharge/charge curves of zinc-iodine battery at 0.5 A g^-1^ with the ZSCCT-2 electrolyte.


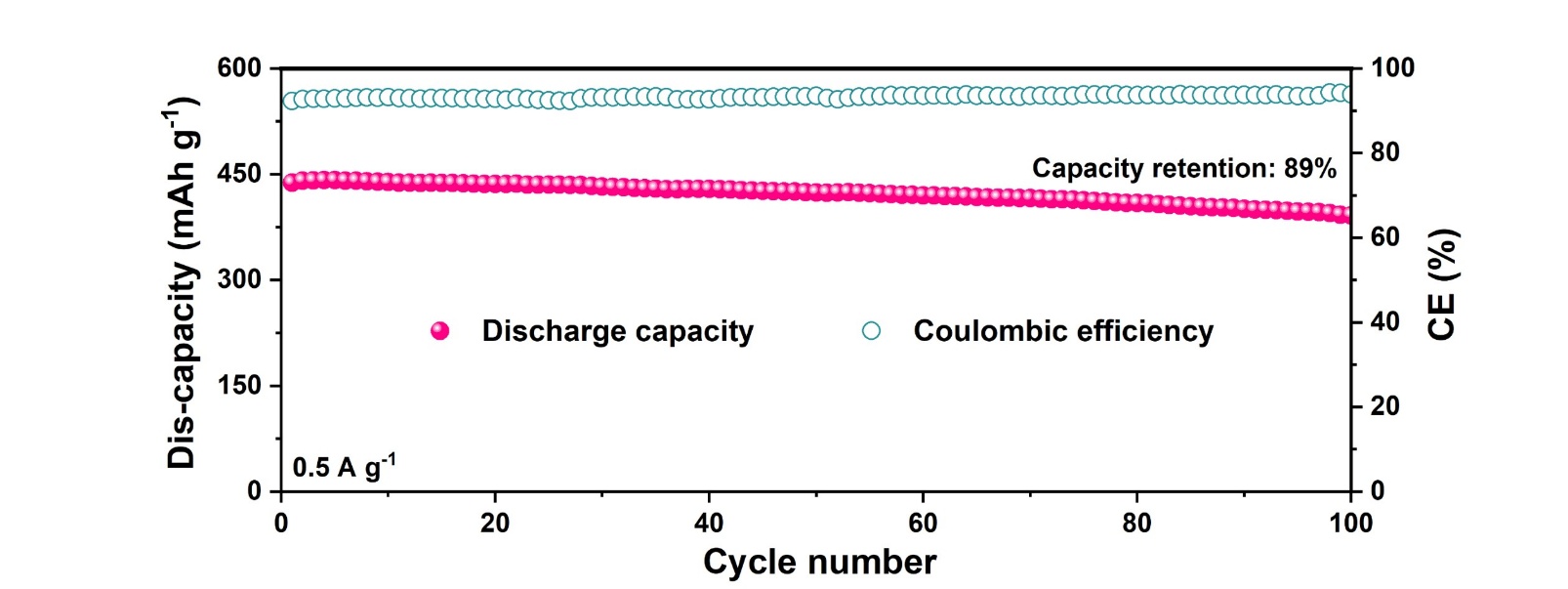


**Figure S67.** Cycling stability of zinc-iodine battery at 0.5 A g^-1^ with the ZSCCT-2 electrolyte.

**
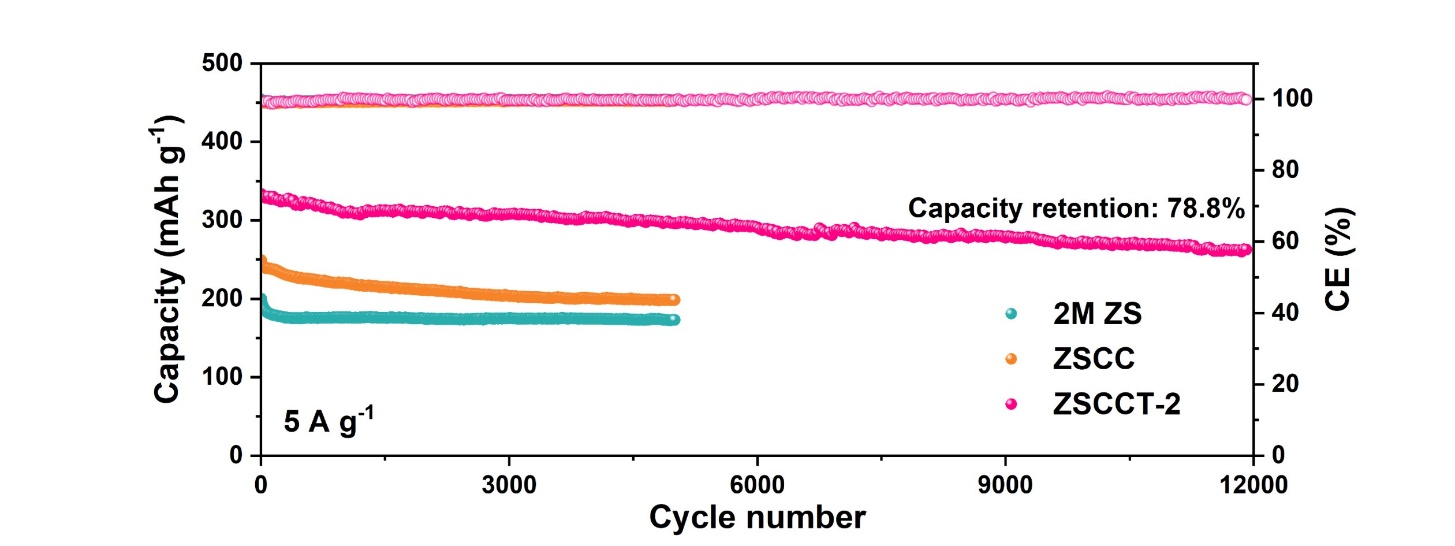
**

**Figure S68.** Cycling stability of zinc-iodine batteries at 5 A g^-1^ in the various electrolytes.

**
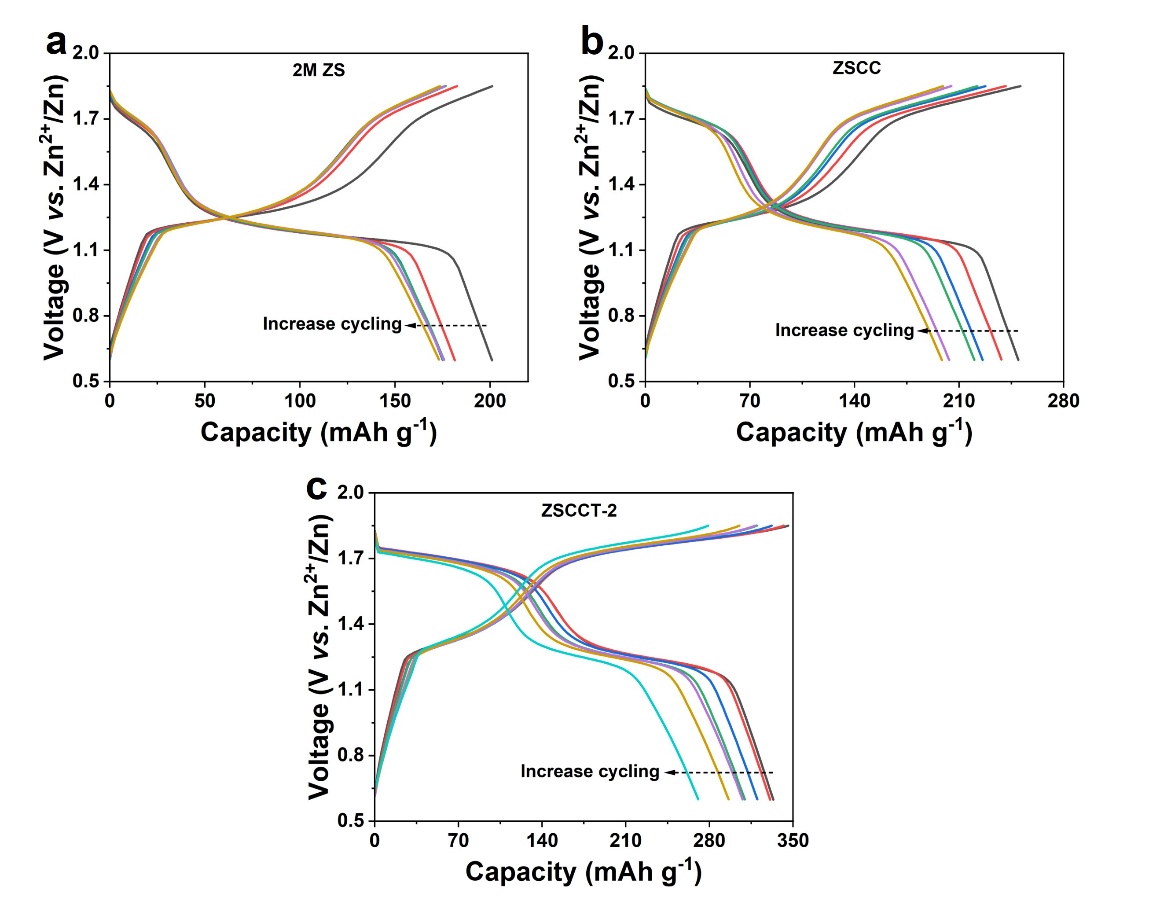
**

**Figure S69.** Galvanostatic discharge/charge curves at 5 A g^-1^ in the various electrolytes.


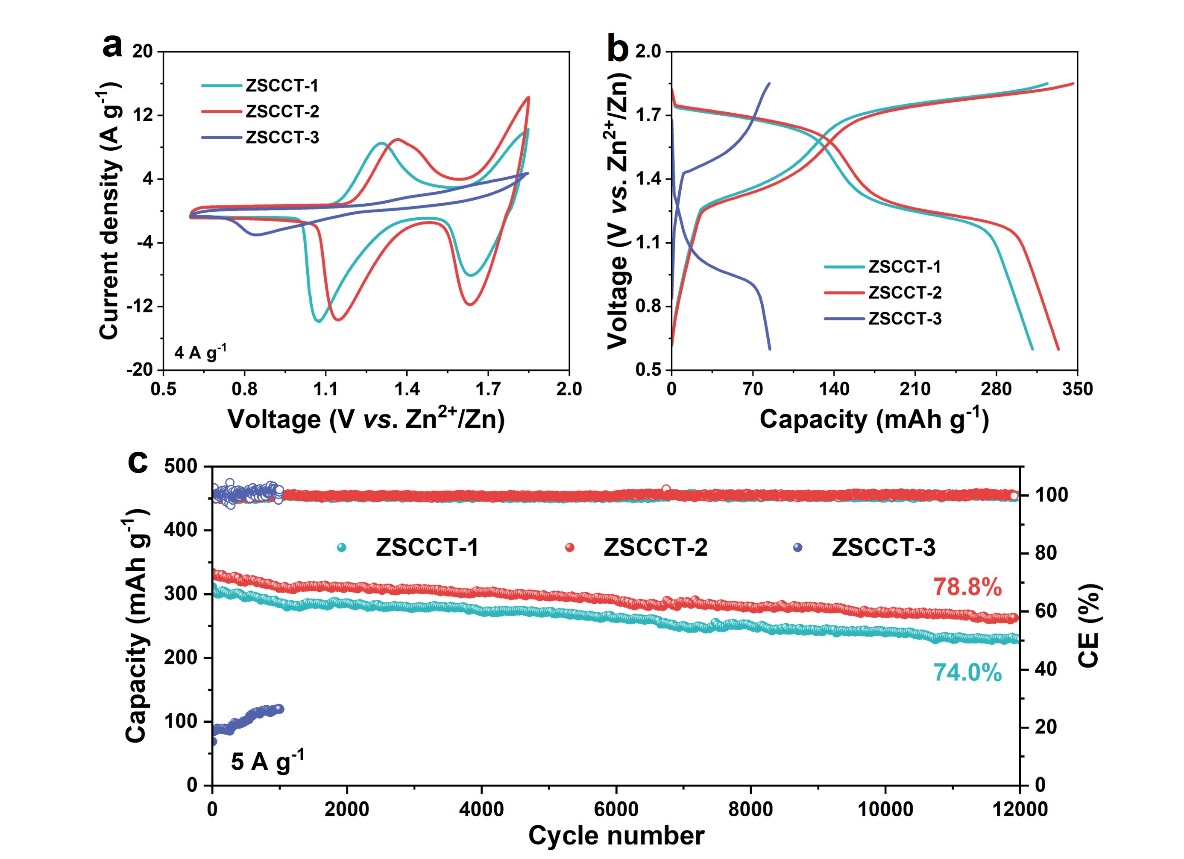


**Figure S70.** The full-cell performance at different TES^-^ concentrations. a) CV curves. b) Galvanostatic discharge/charge curves at 5 A g^-1^. c) Cycling stability at 5 A g^-1^.

The CV curves of the zinc-iodine batteries with the ZSCCT-1 and ZSCCT-2 electrolytes exhibit two pairs of oxidation and reduction peaks, which can be assigned to the reversible reactions of I^0^/I^-^ at low voltage and I^+^/I^0^ at high voltage, respectively. Specifically, the battery with the ZSCCT-2 electrolyte displays a relatively larger response current than the case of ZSCCT-1 electrolyte (Figure S70a), indicating the enhanced reactivity and redox kinetics of I^+^/I^0^/I^-^ conversion in the ZSCCT-2 electrolyte due to the formation of more TES-I-Cl coordination structures. By contrast, the zinc-iodine battery with the ZSCCT-3 electrolyte displays poor reversibility of I^+^/I^0^/I^-^ conversion due to the participation of TES^-^ in the Zn^2+^-solvation shell when the TES^-^ concentration reaches 1M. Moreover, the batteries with the ZSCCT-1 and ZSCCT-2 electrolytes also exhibit a long-term cycling life of over 12,000 cycles with capacity retentions of 74.0% and 78.8% (Figure S70b, c), respectively. However, the battery with the ZSCCT-3 electrolyte displays low discharge capacity and poor cycling performance.

**
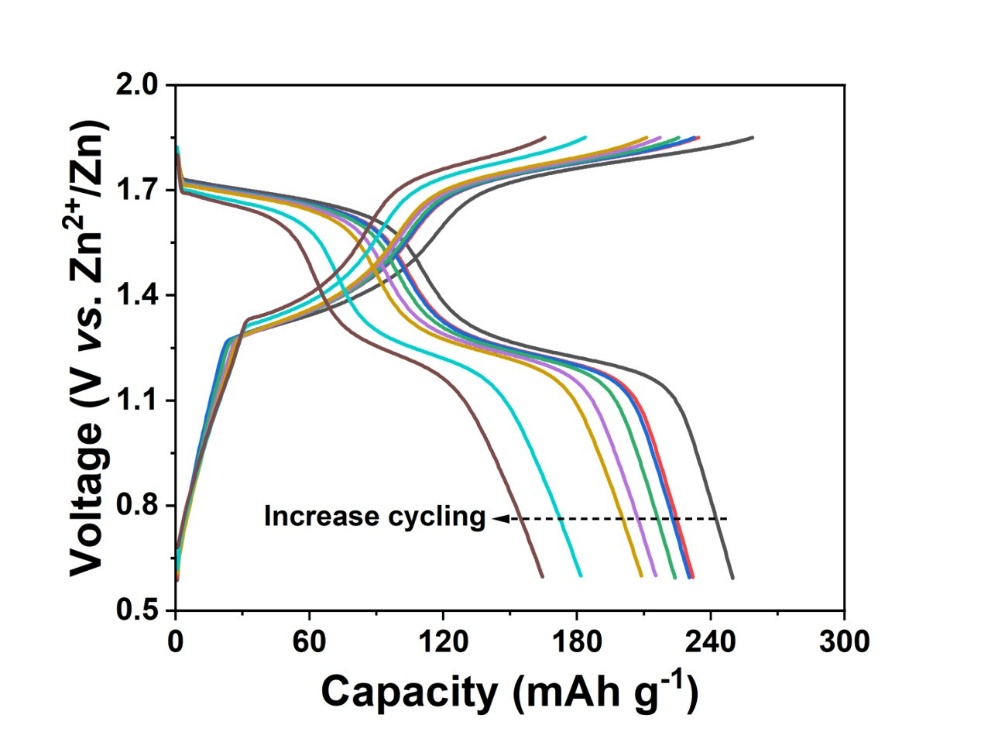
**

**Figure S71.** Galvanostatic discharge/charge curves at 10 A g^-1^.


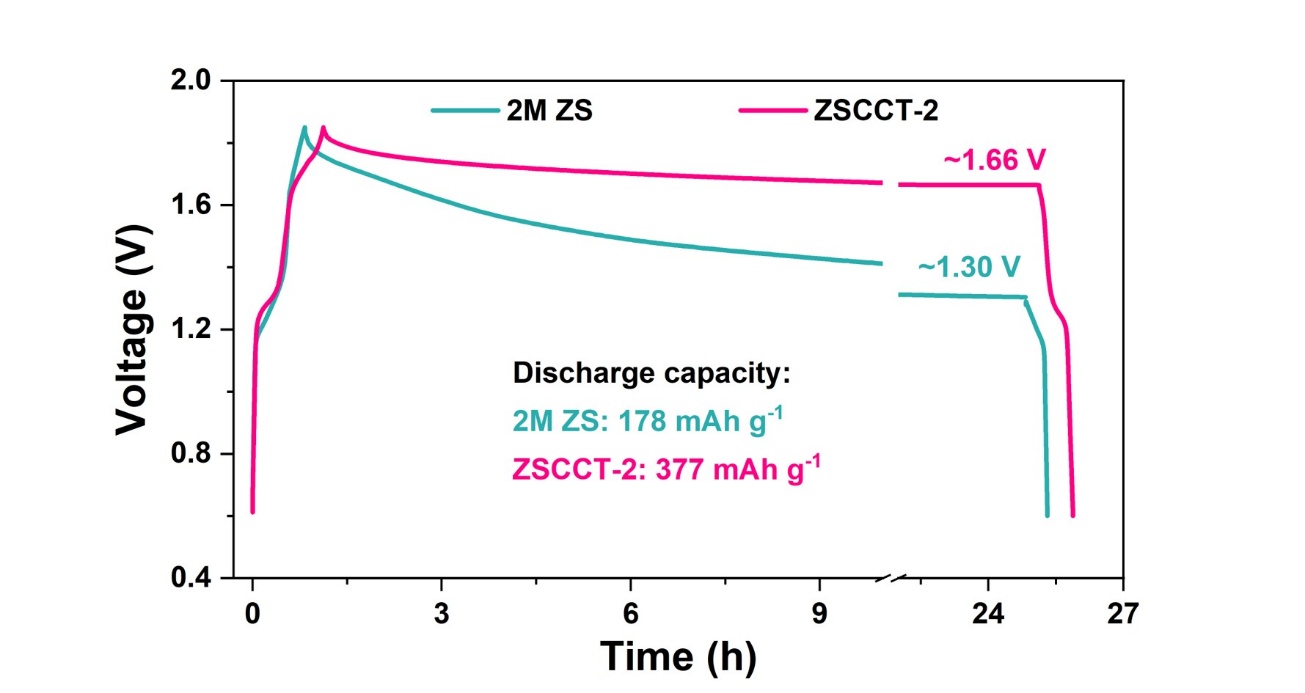


**Figure S72.** Self-discharge capability of the zinc-iodine batteries in the various electrolytes.

**
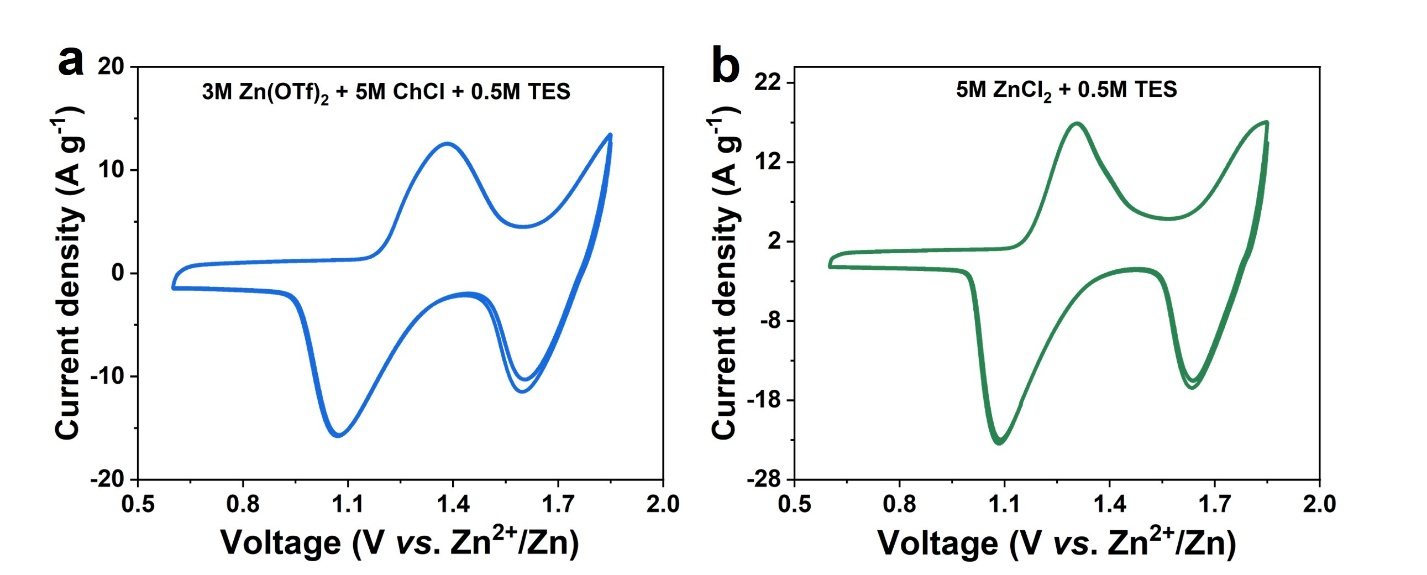
**

**Figure S73.** CV curves of zinc-iodine batteries at 10 mV s^-1^ in the various electrolytes. (a) 3M Zn(OTf)_2_+5M ChCl+0.5M TES and (b) 5M ZnCl_2_+0.5M TES.

**
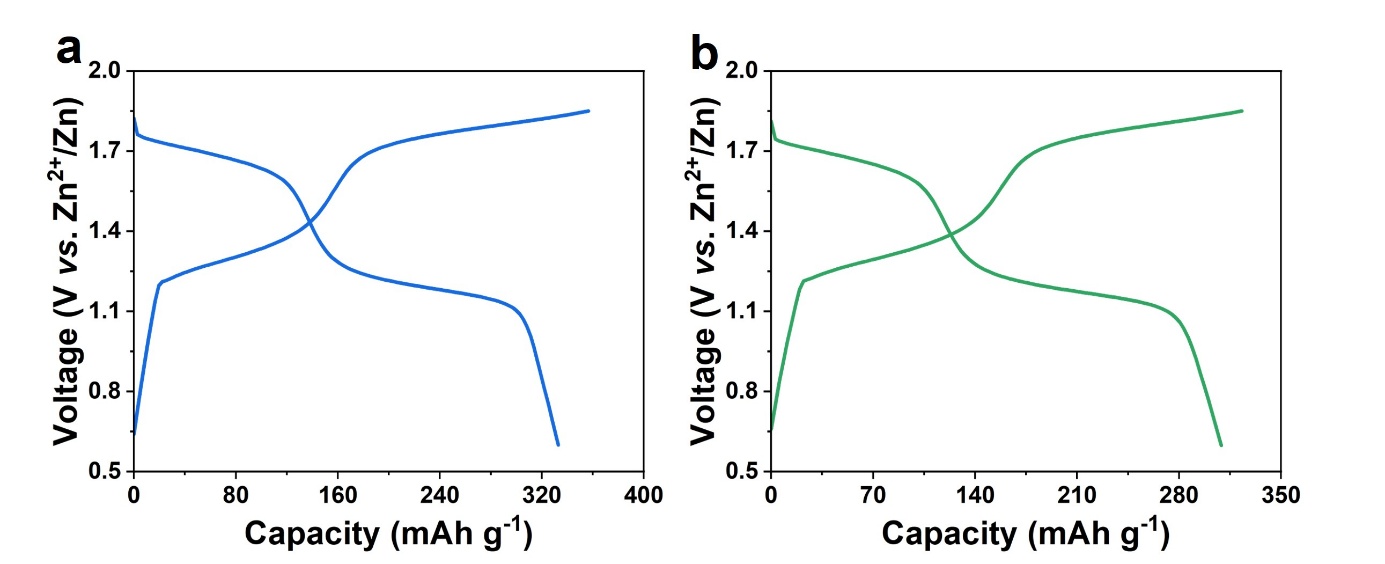
**

**Figure S74.** Galvanostatic discharge/charge curves of zinc-iodine batteries at 5 A g^-1^ in the various electrolytes. (a) 3M Zn(OTf)_2_+5M ChCl+0.5M TES and (b) 5M ZnCl_2_+0.5M TES.

**
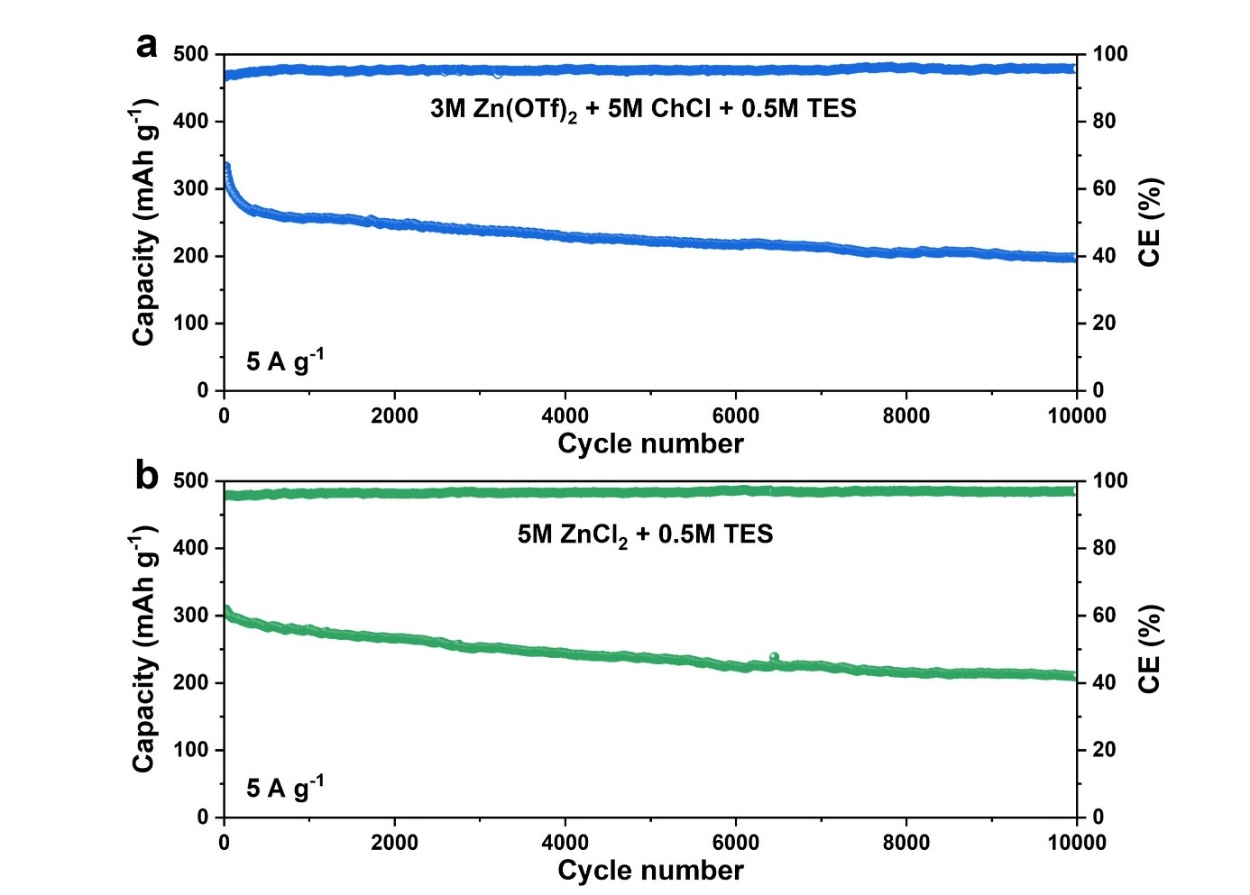
**

**Figure S75.** Cycling stability of zinc-iodine batteries at 5 A g^-1^ over 10,000 cycles in the various electrolytes. (a) 3M Zn(OTf)_2_+5M ChCl+0.5M TES and (b) 5M ZnCl_2_+0.5M TES.


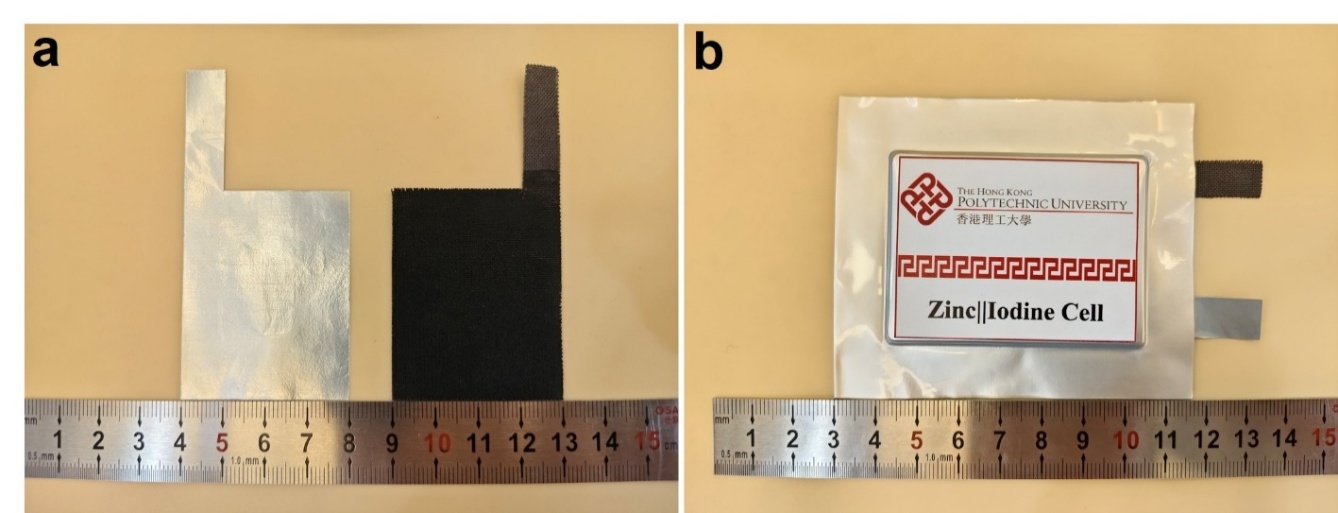


**Figure S76.** (a) The dimensions of the zinc anode and iodine cathode. (b) The digital image of a soft-packaged zinc-iodine battery.


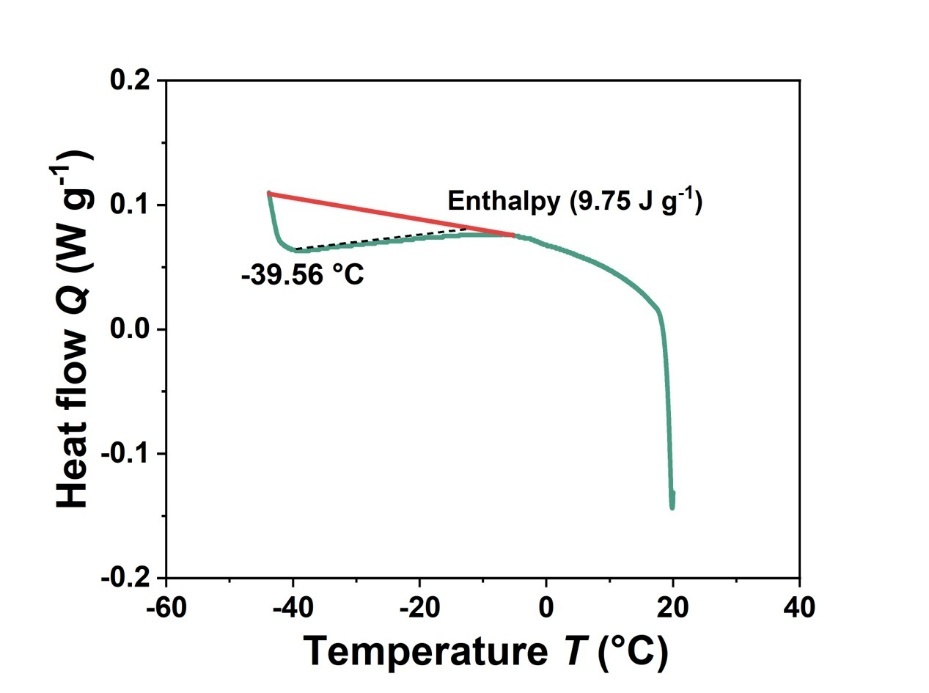


**Figure S77.** The freezing point data of the ZSCCT-2 electrolyte were obtained from the DSC curves.

**
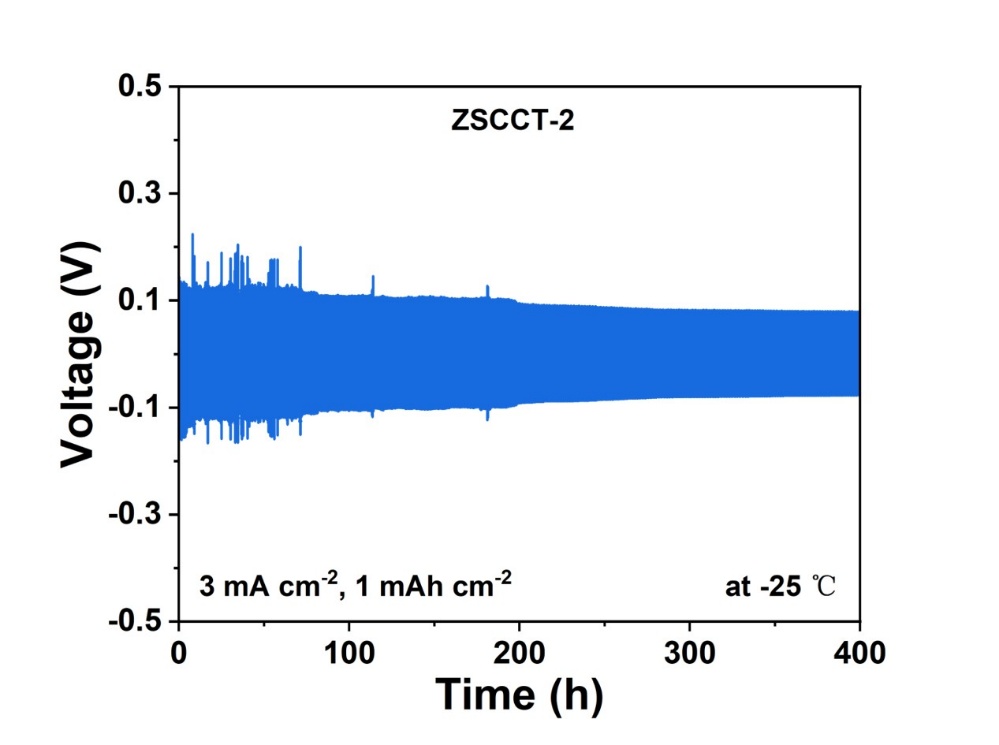
**

**Figure S78.** Cycling performance of Zn||Zn cell with the ZSCCT-2 electrolyte at a low temperature of -25 °C.

**
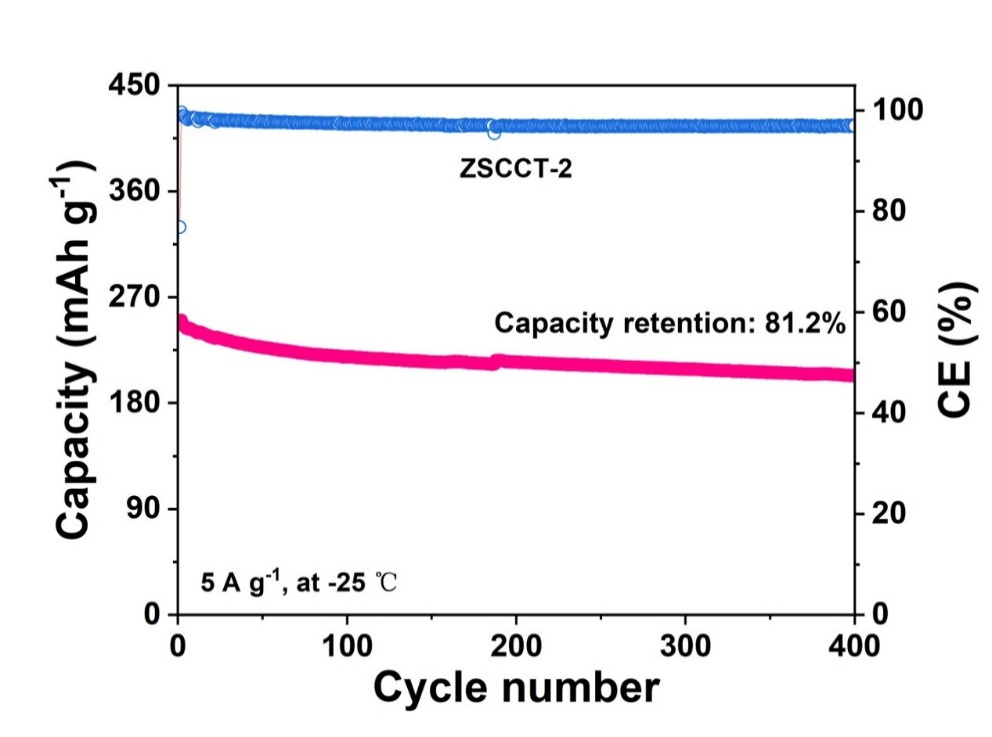
**

**Figure S79.** Cycling stability of zinc-iodine battery with the ZSCCT-2 electrolyte at a low-temperature of -25 °C.


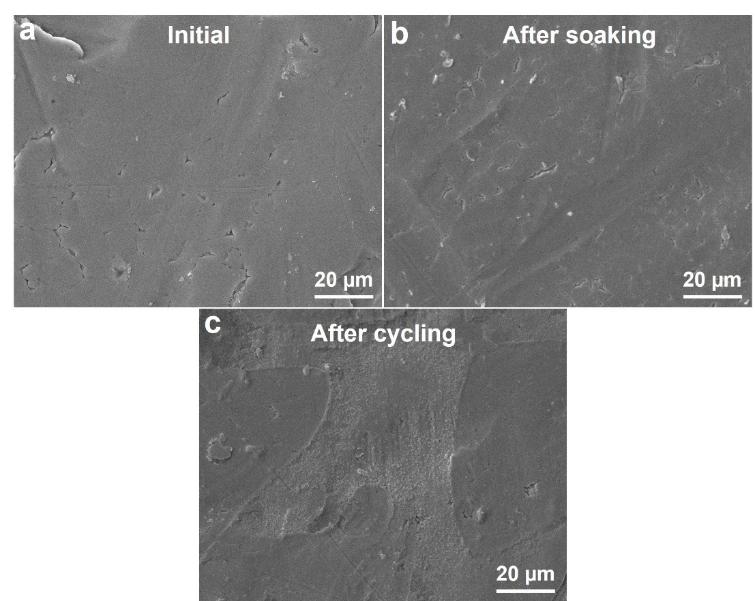


**Figure S80.** SEM images of stainless-steel spacers at different states. (a) Initial, (b) after soaking in the ZSCCT-2 electrolyte (7 days), and (c) after cycling in the ZSCCT-2 electrolyte.

**Table S1.** The interfacial tension (IFT) values of the various electrolytes.

| **Liquid** | **Test 1** | **Test 2** | **Test 3** | **Test 4** | **Test 5** | **Average** |
| --- | --- | --- | --- | --- | --- | --- |
| 2M ZS | 77.07 | 77.62 | 77.88 | 78.00 | 77.80 | 77.67 |
| ZSCC | 76.69 | 77.14 | 76.92 | 76.9 | 77.29 | 76.99 |
| ZSCCT-2 | 75.43 | 76.06 | 75.95 | 75.84 | 75.64 | 75.78 |

Note: The unit is mN m^-1^. The test temperature is 25 °C.

**Table S2.** The sizes of MD simulation systems of the various electrolytes.

| **Surface** | ***X*, *Y*, *Z* (Å)** | **System size** |
| --- | --- | --- |
| 2M ZS | 39.64, 39.64, 39.64 | (H_2_O)_2000_(ZnSO_4_)_72_ |
| ZSCC | 42.51, 42.51, 42.51 | (H_2_O)_1500_(ZnSO_4_)_54_(ChCl)_135_ |
| ZSCCT-1 | 42.90, 42.90, 42.90 | (H_2_O)_1500_(ZnSO_4_)_54_(ChCl)_135_(NaTES)_6_ |
| ZSCCT-2 | 43.04, 43.04, 43.04 | (H_2_O)_1500_(ZnSO_4_)_54_(ChCl)_135_(NaTES)_14_ |
| ZSCCT-3 | 43.60, 43.60, 43.60 | (H_2_O)_1500_(ZnSO_4_)_54_(ChCl)_135_(NaTES)_27_ |

**Table S3.** The EIS fitted parameters of zinc-iodine batteries with 2M ZS and ZSCCT-2 electrolytes (data from Figure S57).

| **Electrolyte** | ***R*_s_ (ohm)** | ***R*_ct_ (ohm)** | ***CPE*_1_-T** | ***CPE*_1_-P** | ***W*_1_-R** | ***W*_1_-T** | ***W*_1_-P** |
| --- | --- | --- | --- | --- | --- | --- | --- |
| 2M ZS | 2.911 | 50.30 | 1.56E10-3 | 0.532 | 95.77 | 55.23 | 0.758 |
| ZSCCT-2 | 2.327 | 16.27 | 7.15E10-3 | 0.436 | 75.97 | 71.76 | 0.650 |

Note: The circuit includes the following components.

*R*_s_: Ohmic resistance of the electrolyte and electrodes.

*R*_ct_: Charge transfer resistance at the electrode-electrolyte interface.

*CPE*: Constant phase element representing double-layer capacitance.

*W*: Warburg diffusion impedance.

**Table S4.** Comparison of electrochemical performance of metal-iodine batteries.[^5-42^](#_ENREF_5)

| **Anode** | **Cathode** | **Electrolyte** | **Current density/capacity (mAh g^-1^)** | **Cycle number/capacity retention (%)** | **Ref.** |
| --- | --- | --- | --- | --- | --- |
| Li | I_2_/KB | PEO:LiTFSI= 18:1 in acetonitrile | 5 C/117 | 4,000/83.5 | 5 |
|  | I_2_/HCM | 0.5M LiTFSI/0.1M LiNO_3_ in dioxolane/dimethoxyethane (1:1, v/v) | 50 C/120.2 | 4,000/NA | 6 |
|  | I_2_/BPD-HI | 1M LiTFSI/2 wt.% LiNO_3_ in DME/DOL (1:1, v/v) | 2 A g^-1^/327 | 850/85 | 7 |
|  | I_2_/N-Graphene | 1M LiPF_6_/1 wt.%  LiNO_3_ in EC/DMC  (1:1, v/v) | 20 C/189.3 | 1,000/95.8 | 8 |
|  | I_2_/C | 0.5M LiTFSI/0.5M  LiNO_3_ in DOL/DME  (1:1, v/v) | 5 C/132 | 3,000/79.7 | 9 |
|  | I_2_/MXene | 1M LiTFSI/1 wt.%  LiNO_3_ in DOL/DME  (1:1, v/v) | 20 C/85.6 | 1,000/78 | 10 |
| Na | I_2_/NiAl-LDH | 1M NaPF_6_ in EC/PC (1:1, v/v) | 1.5 A g^-1^/~100 | 2,000/NA | 11 |
|  | I_2_/γ-Mo_2_N/NC | 1M NaClO_4_ in EC/DEC (1:1, v/v) | 3.55 C/213.5 | 800/NA | 12 |
|  | I_2_/3D-COFs-Co | 1M NaClO_4_ in EC/DEC (1:1, v/v) with 5 wt.%FEC | 10 C/142.5 | 2,000/80.9 | 13 |
|  | CNT/GO/iodinene | 1M NaClO_4_ in EC/DEC (1:1, v/v) | 47 C/109.5 | 5,000/~75 | 14 |
|  | I_2_/polypyrrole | 1M NaClO_4_ in EC/DEC (1:1, v/v) | 5 C/55 | 400/52 | 15 |
|  | I_2_/MOF | 1M NaClO_4_ in EC/DEC (1:1, v/v) | 11.8 C/127 | 3,200/95 | 16 |
| K | I_2_/MXene@CNP | 1M KFSI in EC/DEC (1:1, v/v) | 10 A g^-1^/97.2 | 3,000/80.1 | 17 |
|  | I_2_/CMK-3 | 1M KPF_6_ in PC/DEC (1:1, v/v) | 0.17 A g^-1^/126.3 | 300/NA | 18 |
|  | I_2_/C composite | 0.5 M KPF_6_ in EC/DEC (1:1, v/v) | 0.4 A g^-1^/~40 | 500/71 | 19 |
| Mg | I_2_/ACC | (HMDS)_2_Mg/AlCl_3_/Mg  Cl_2_ in TEGDME | 1 C/~140 | NA | 20 |
|  | I_2_/Mg-PVPI | (HMDS)_2_Mg/AlCl_3_/Mg  Cl_2_ in TEGDME | 0.8 A g^-1^/~100 | 500/NA | 21 |
| Al | I_2_/AC-PVPI | AlCl_3_/1-ethyl-3-  methylimidazolium  chloride (1.3:1, m/m) | 1 C/102.7 | 1,050/NA | 22 |
|  | PVP-I_2_ | AlCl_3_/1-ethyl-3-  methylimidazolium  chloride (1.3:1, m/m) | 1 C/133 | 150/NA | 23 |
|  | I_2_/ZIF-8-C | 9M LiTFSI/1M AlCl_3_ | 2 A g^-1^/162 | 150/62.5 | 24 |
| Zn | Co@AC/I_2_ | 2M Zn(CF_3_SO_3_)_2_ | 5 C/112.3 | 1,000/68 | 25 |
|  | I_2_/AC | PVA/Urea/zinc ion hydrogel | 1 A g^-1^/80.3 | 8,000/73 | 26 |
|  | KB-I_2_ | 2M ZnCl_2_/1 wt.%SP | 10 C/~160 | 6,000/91.1 | 27 |
|  | I_2_/AC | Cation-conduction dominated hydrogel | 10 C/~150 | 22,000/83.3 | 28 |
|  | M9 | 2M ZnSO_4_ | 2 A g^-1^/162 | 10,000/NA | 29 |
|  | TBA-I_3_ | 0.5M KOH/0.1M ZnAc/saturated KCl | 1 A g^-1^/~280 | 350/70 | 30 |
|  | I_2_/ACC-PVPI | 4M Zn(OTf)_2_/6M urea | 5 C/147 | 10,000/NA | 31 |
|  | I_2_@AC | 15m ZnCl_2_-TEA^+^ | 10 A g^-1^/~138 | 35,000/47.5 | 32 |
|  | I_2_/AC | 2M ZnSO_4_/500mM TAH | 2 A g^-1^/~280 | 5,000/70 | 33 |
|  | Ti_3_C_2_I_2_ | 2M ZnSO_4_/1M KCl | 3 A g^-1^/~175 | 2,800/~71.4 | 34 |
|  | BiI_3_ | 2M ZnSO_4_/0.3m KCl | 1 A g^-1^/~370 | 500/~81.1 | 35 |
|  | I_2_@AC | WiDES-13 | 0.2 A g^-1^/309 | 1500/82.4 | 36 |
|  | KB-I_2_ | ZS/NaCl-0.2HMTA | 4 A g^-1^/264 | 5000/82.1 | 37 |
|  | I_2_@Q-PAN | 3/5ZC | 10 C/~200 | 11000/87 | 38 |
|  | BMICl-based | 2M ZnSO_4_/0.5M NaCl | 10 C/~260 | 2000/81.5 | 39 |
|  | pMA@AC/I | 2M ZnSO_4_/0.2M ZnI_2_ | 23.55 mA/210 | 10000/80 | 40 |
|  | C_3_H_9_IS/TZ-COFs | MPIBr-containing electrolyte | 1 A g^-1^/1296 | 1,200/86.3 | 41 |
|  | I_2_/HAC | 1M ZnSO_4_/0.1M H_2_SO_4_/0.1M KBr | 2 A g^-1^/~1050 | 150/76.2 | 42 |
| **Zn** | **I_2_@Ac** | **2M ZnSO_4_/5M ChCl/0.5M TES** | **5 A g^-1^/~263** | **12,000/~78.8** | **This work** |
|  | **I_2_@Ac** | **2M ZnSO_4_/5M ChCl/0.5M TES** | **10 A g^-1^/~163** | **42,000/~70** | **This work** |

**Table S5.** Key parameters of the soft-packaged zinc-iodine battery.

| **Parameter** | **Value** | **Notes** |
| --- | --- | --- |
| Anode dimension | 76.2 mg (Length × Width × Thickness: 5 cm × 4 cm × 10 µm) | / |
| Cathode dimension | 422 mg (Length × Width: 5 cm × 4 cm) | / |
| Areal loading (cathode) | 7.50 mg cm^-2^ | Active material mass per area |
| Areal loading (anode) | 3.82 mg cm^-2^ | Active material mass per area |
| N:P ratio | 1.12 | Negative/positive capacity ratio |
| Total capacity | 40.7 mAh | Measured at 0.2 A g^-1^ |
| Full-cell energy density | 269.9 Wh kg^-1^ | Based on the total mass of active materials in both electrodes |

**Supplementary Notes**

**Ionic conductivity:** The ionic conductivity (σ) of the electrolyte system was determined using the following equation:

σ = $\frac{l}{RA}$ (2)

where *l* is the distance between electrodes, *R* is the impedance, and *A* is the contact area between electrolyte and electrode.

**Capacitive contribution:** The ratio of capacitive contribution under different scan rates can be calculated through the following equation:[^43^](#_ENREF_43)

$i(V)$ = $k_{1}v+k_{2}v^{1/2}$ (3)

where *i*, *k*_1_*v*, and *k*_2_*v*^1/2^ correspond to the current, capacitive, and ionic diffusion contribution, respectively. Under a fixed potential, the current response can be separated into capacitive and ionic diffusion contributions. The equation (3) can be rearranged as follows:

$i(V)/v^{1/2}$ = $k_{1}v^{1/2}+k_{2}$ (4)

**Zn^2+^ transference number measurements:** Ion transference number (*t*) was determined by combination measurements of AC impedance and direct current polarization using Zn‖Zn symmetric cells in various electrolytes. The polarization currents of the cell contain initial current (*I*_0_) and steady-state current (*I*_s_), which are recorded under a direct current polarization voltage of 10 mV for 60 min. The interfacial resistances before (*R*_o_) and after (*R*_s_) polarization were measured by EIS. The *t* was then determined by the following equation:[^44^](#_ENREF_44)

*t* = $\frac{I_{s}(\Delta V-I_{o}R_{0})}{I_{0}(\Delta V-I_{s}R_{s})}$ (5)

**Corrosion of stainless-steel components by chloride ions:** In our work, Figure S80 shows the SEM images of the initial stainless-steel spacer, the soaked spacer in the optimal electrolyte for one week, and the spacer after cycling. There is no significant morphological change or corrosion observed on the stainless-steel surface after exposure to the choline chloride-based electrolyte, either after soaking or after electrochemical cycling. The relatively low corrosivity of chloride ions in choline chloride towards stainless steel in our system can be attributed to the following reasons: 1) The bulky choline cation (Ch^+^) in ChCl can adsorb on metal surfaces, forming a protective layer that partially blocks Cl^-^ access (similar to TES^-^ shielding in the ZSCCT-2 electrolyte); 2) ChCl solutions (pH≈7) are significantly less corrosive than acidic chloride salts; 3) Strong Ch⁺-Cl^-^ association reduces free Cl^-^ activity compared to fully dissociated salts like NaCl; 4) Forming stable TES-I-Cl coordination structure, further reducing free Cl^-^ activity (synergistic protection effect in our system).

**N:P ratio:** The N:P ratio is defined as the ratio of the practical capacity of the anode (mAh) to that of the cathode. 1) Anode capacity: Zinc possesses a theoretical capacity of 820 mAh g^-1^, corresponding to the Zn ↔ Zn^2+^ + 2e^-^ redox reaction. However, the practical capacity of zinc is typically lower due to factors such as polarization, dendrite formation, and side reactions. In our work, a practical capacity of ~600 mAh g^-1^ was adopted; 2) Cathode capacity: iodine has a theoretical capacity of 422 mAh g^-1^, based on the four-electron I^-^/I^0^/I^+^ redox process. The practical capacity of iodine, as determined from discharge curves, was measured to be 271.3 mAh g^-1^, which reflects kinetic limitations and the dissolution of iodine during cycling. Therefore, the N:P ratio can be calculated through the following equation:

$N:P=\frac{Practical capacity of anode (mAh)}{Practical capacity of cathode (mAh)}=\frac{600*76.2/1000}{271.3*150/1000}=1.12$ (6)

**Full-cell energy density:** The gravimetric energy density of the full cell was calculated according to the following equation:

$Energy density \left( {Wh kg}^{-1} \right)=\frac{Discharge capacity \left( \mathrm{Ah} \right)*Average discharge voltage (V)}{Total mass of active materials in both electrodes (kg)}=\frac{40.7/1000*1.5}{(76.2+150)/1000000}\left( {Wh kg}^{-1} \right)=269.9 \left( {Wh kg}^{-1} \right)$ (7)

**References**

1. T. H. Wan, M. Saccoccio, C. Chen and F. Ciucci, *Electrochim. Acta*, 2015, **184**, 483-499.

2. Y. Lu, C.-Z. Zhao, J.-Q. Huang and Q. Zhang, *Joule*, 2022, **6**, 1172-1198.

3. B. Delley, *J. Chem. Phys.*, 2000, **113**, 7756-7764.

4. L. Kong, G. Yan, K. Hu, Y. Yu, N. Conte, K. R. McKenzie Jr, M. J. Wagner, S. G. Boyes, H. Chen, C. Liu and X. Liu, *Nat. Commun.*, 2025, **16**, 806.

5. Z. Cheng, H. Pan, F. Li, C. Duan, H. Liu, H. Zhong, C. Sheng, G. Hou, P. He and H. Zhou, *Nat. Commun.*, 2022, **13**, 125.

6. Z. Su, H. Y. Ling, M. Li, S. Qian, H. Chen, C. Lai and S. Zhang, *Carbon Energy*, 2020, **2**, 265-275.

7. F. Zhu, Z. Li, Z. Wang, Y. Fu and W. Guo, *J. Am. Chem. Soc.*, 2024, **146**, 11193-11201.

8. Z. Su, Z. Wei, C. Lai, H. Deng, Z. Liu and J. Ma, *Energy Storage Mater.*, 2018, **14**, 129-135.

9. L. Qiao, C. Wang and X. S. Zhao, *ACS Appl. Energy Mater.*, 2021, **4**, 7012-7019.

10. C. Sun, X. Shi, Y. Zhang, J. Liang, J. Qu and C. Lai, *ACS Nano*, 2020, **14**, 1176-1184.

11. L. Feng, Y. Gong and J. Lin, *Chem. Eng. J.*, 2024, **493**, 152612.

12. T. Zhang, F. Wei, Y. Wu, W. Li, L. Huang, J. Fu, C. Jing, J. Cheng and S. Liu, *Adv. Sci.*, 2023, **10**, 2301918.

13. C. Guo, T. Liu, Z. Wang, Y.-X. Wang, M. Steven, Y. Luo, X. Luo and Y. Wang, *Angew. Chem., Int. Ed.*, 2025, **64**, e202415759.

14. M. Qian, Z. Xu, Z. Wang, B. Wei, H. Wang, S. Hu, L.-M. Liu and L. Guo, *Adv. Mater.*, 2020, **32**, 2004835.

15. L. Xiang, S. Yuan, F. Wang, Z. Xu, X. Li, F. Tian, L. Wu, W. Yu and Y. Mai, *J. Am. Chem. Soc.*, 2022, **144**, 15497-15508.

16. F. Wang, Z. Liu, C. Yang, H. Zhong, G. Nam, P. Zhang, R. Dong, Y. Wu, J. Cho, J. Zhang and X. Feng, *Adv. Mater.*, 2020, **32**, 1905361.

17. S. Zhao, B. Zhang, L. Li, P. Zhang, G. Li, Z. Zhu, Y. Choi, L. Dong, M. Luo and S. Guo, *J. Am. Chem. Soc.*, 2025, **147**, 669-677.

18. M. Qian, M. Tang, J. Yang, W. Wei, M. Chen, J. Chen, J. Xu, Q. Liu and H. Wang, *J. Colloid Interf. Sci.*, 2019, **551**, 177-183.

19. K. Lu, H. Zhang, F. Ye, W. Luo, H. Ma and Y. Huang, *Energy Storage Mater.*, 2019, **16**, 1-5.

20. H. Tian, T. Gao, X. Li, X. Wang, C. Luo, X. Fan, C. Yang, L. Suo, Z. Ma, W. Han and C. Wang, *Nat. Commun.*, 2017, **8**, 14083.

21. Y. Zhang, D. Tao, F. Xu and T. Li, *Chem. Eng. J.*, 2022, **427**, 131592.

22. S. Zhang, X. Tan, Z. Meng, H. Tian, F. Xu and W.-Q. Han, *J. Mater. Chem. A*, 2018, **6**, 9984-9996.

23. H. Tian, S. Zhang, Z. Meng, W. He and W.-Q. Han, *ACS Energy Lett.*, 2017, **2**, 1170-1176.

24. S. Yang, C. Li, H. Lv, X. Guo, Y. Wang, C. Han, C. Zhi and H. Li, *Small Methods*, 2021, **5**, 2100611.

25. L. Zhu, X. Guan, Y. Fu, Z. Zhang, Y. Li, Q. Mai, C. Zhang, Z. Yuan, Y. Wang, P. Li, H. Li, D. Su, B. Jia, H. Yu, Y. Sun and T. Ma, *Adv. Funct. Mater.*, 2024, **34**, 2409099.

26. X. Zhou, S. Huang, L. Gao, Z. Zhang, Q. Wang, Z. Hu, X. Lin, Y. Li, Z. Lin, Y. Zhang, Y. Tang, Z. Wen, M. Ye, X. Liu and C. C. Li, *Angew. Chem., Int. Ed.*, 2024, **63**, e202410434.

27. S.-J. Zhang, J. Hao, H. Wu, Q. Chen, C. Ye and S.-Z. Qiao, *Adv. Mater.*, 2024, **36**, 2404011.

28. J.-L. Yang, T. Xiao, T. Xiao, J. Li, Z. Yu, K. Liu, P. Yang and H. J. Fan, *Adv. Mater.*, 2024, **36**, 2313610.

29. X. Guo, H. Xu, Y. Tang, Z. Yang, F. Dou, W. Li, Q. Li and H. Pang, *Adv. Mater.*, 2024, **36**, 2408317.

30. X. Li, T. Liu, P. Li, G. Liang, Z. Huang, Z. Chen, A. Chen, Y. Su, L. Yang, D. Cao and C. Zhi, *ACS Nano*, 2025, **19**, 2900-2908.

31. C. Li, H. Li, X. Ren, L. Hu, J. Deng, J. Mo, X. Sun, G. Chen and X. Yu, *ACS Nano*, 2025, **19**, 2633-2640.

32. W. Zong, J. Li, C. Zhang, Y. Dai, Y. Ouyang, L. Zhang, J. Li, W. Zhang, R. Chen, H. Dong, X. Gao, J. Zhu, I. P. Parkin, P. R. Shearing, F. Lai, K. Amine, T. Liu and G. He, *J. Am. Chem. Soc.*, 2024, **146**, 21377-21388.

33. M. Wang, Y. Meng, M. Sajid, Z. Xie, P. Tong, Z. Ma, K. Zhang, D. Shen, R. Luo, L. Song, L. Wu, X. Zheng, X. Li and W. Chen, *Angew. Chem., Int. Ed.*, 2024, **63**, e202404784.

34. X. Li, M. Li, Z. Huang, G. Liang, Z. Chen, Q. Yang, Q. Huang and C. Zhi, *Energy Environ. Sci.*, 2021, **14**, 407-413.

35. X. Li, W. Xu, J. Feng, Z. Liu, N. Jiang, L. Ye, Y. Gao, Y. Ma, Z. Tao, Y. Duan, X. Li, Q. Yang and J. Qiu, *Nano Energy*, 2025, **138**, 110884.

36. X. Zhang, L. Su, R. Xu, J. Li, F. Xie, X. Xu, F. Lu, L. Zheng, H. Wang, C. Ouyang and X. Gao, *Adv. Energy Mater.*, 2026, **16**, e05649.

37. S.-J. Zhang, J. Hao, H. Wu, Q. Chen, Y. Hu, X. Zhao and S.-Z. Qiao, *J. Am. Chem. Soc.*, 2025, **147**, 16350-16361.

38. L. Zhang, J. Gong, H. Guo, J. Huang, S. Chen, J.-F. Gohy, Y. Zhou, J. Hofkens, T. Liu, K. Müllen and F. Lai, *Adv. Mater.*, 2026, **38**, e14117.

39. H. Wu, S.-J. Zhang, J. Vongsvivut, Y. Jiang, J. Hao and S.-Z. Qiao, *Adv. Mater.*, 2026, **38**, e11680.

40. Y. Wang, Y. Lv, S. Wei, L. Yu, B. Yuan, T. A. Shifa, M. Muhammad, R. Wang, J. Li, Y. Zhao and X. Sun, *Adv. Mater.*, 2026, **38**, e15000.

41. W. Du, Q. Huang, X. Zheng, Y. Lv, L. Miao, Z. Song, L. Gan and M. Liu, *Energy Environ. Sci.*, 2025, **18**, 6540-6547.

42. W. Ma, T. Liu, C. Xu, C. Lei, P. Jiang, X. He and X. Liang, *Nat. Commun.*, 2023, **14**, 5508.

43. S. Wang, Z. Yuan, X. Zhang, S. Bi, Z. Zhou, J. Tian, Q. Zhang and Z. Niu, *Angew. Chem., Int. Ed.*, 2021, **60**, 7056-7060.

44. S. Zugmann, M. Fleischmann, M. Amereller, R. M. Gschwind, H. D. Wiemhöfer and H. J. Gores, *Electrochim. Acta*, 2011, **56**, 3926-3933.
